# Global, Regional, and National Epidemiology of Depression in Working-Age Individuals, 1990–2019

**DOI:** 10.1155/2024/4747449

**Published:** 2024-08-24

**Authors:** Jin-shuai Yang, Lu-yu Zhang, Cheng-hao Yang, Xin-yu Li, Zhi-qiang Li

**Affiliations:** ^1^Department of Neurology, Shangqiu First People's Hospital, Shangqiu, Henan 476100, China; ^2^Department Urology, Shangqiu First People's Hospital, Shangqiu, Henan 476100, China; ^3^Department of Vascular Surgery, Shanghai Putuo People's Hospital, School of Medicine, Tongji University, Shanghai, China; ^4^Department of Plastic and Reconstructive Surgery, Shanghai Ninth People's Hospital, Shanghai Jiao Tong University, Shanghai, China

## Abstract

**Background:**

Depression is a disorder that can have a serious impact on functioning and quality of life. Understanding the global, regional, and national epidemiology of depression in working-age populations (15–49 years) is important for informing mental health policies and services. However, up-to-date data have been lacking, especially in developing regions.

**Methods:**

This study delved into the prevalence of depression among individuals in the working-age group, specifically those between 15 and 49 years, by analyzing data from the 2019 Global Burden of Disease, Injuries, and Risk Factors Study (GBD). The comprehensive analysis aimed to determine the age-standardized prevalence, incidence, and disability-adjusted life years (DALYs) associated with depression across diverse groups. It considered factors such as geographical regions, age brackets, genders, and sociodemographic indices, spanning a time frame from 1990 to 2019.

**Results:**

Globally, the estimated prevalent cases, incident cases, and DALYs for depression increased from 1990 to 2019. Regionally, certain regions like Central Latin America saw rapid increases in age-standardized prevalence and incidence rates over time. By sociodemographic regions, all tiers witnessed rises in incident cases, with high sociodemographic index (SDI) areas having the greatest burden in 2019. Nationally, countries such as India, China, and the United States had the highest total prevalence and incidence in 2019, while nations like Yemen and Angola reported exceptionally high age-standardized rates (ASRs). Peak prevalence risk occurred in the mid-to-late 40s age range. Period effects showed declining risks over time. Earlier birth cohorts, especially before the mid-1960s, faced higher risks than more recent generations. Population growth rather than epidemiological changes appeared to drive increases in disease burden.

**Conclusion:**

From 1990 to 2019, the overall trend of depression burden in working-age individuals presents regional and national variations and differs by age, sex, period, and cohort.

## 1. Introduction

Depression is one of the leading causes of disability worldwide, contributing substantially to the global burden of disease [1]. Globally, an estimated 5% of adults suffer from depression in any given year [2]. Depression can negatively impact functioning and quality of life across multiple domains, including work, relationships, and physical health [3]. The peak period of onset is commonly in the mid-late 20s, meaning depression in working-age individuals can have detrimental personal and socioeconomic effects [4].

This study focuses specifically on working-age populations. This age range covers the prime working years in most countries when people are contributing actively to the economy and society before reaching older age. Mental health conditions like depression that arise in these critical years can have outsized impacts on personal development as well as socioeconomic outcomes. For example, onset of depression during the working years can negatively impact education completion, workforce participation and productivity, and earning trajectories over the lifespan [5, 6]. There can also be deleterious impacts on family dynamics and functioning when parents or caregivers experience depression [7].

Understanding the epidemiology of depression—including prevalence, incidence, duration, and burden—is vital for informing policy, planning services, targeting interventions, and allocating health resources [8]. The Global Burden of Disease, Injuries, and Risk Factors Study (GBD) offers a unique opportunity to characterize the epidemiology of health loss globally across age groups in a standardized approach over time. The purpose of this study is to provide an up-to-date and expanded analysis of the global, regional, and national burden and trends of depression specifically in the working-age population aged 15–64 years. By focusing on the working-age demographic, this study seeks to fill a key evidence gap and draw attention to the substantial personal and socioeconomic impacts of depression during the economically productive years.

## 2. Methods

### 2.1. Data Source and Definitions

Data pertaining to the prevalence, incidence, and disability-adjusted life years (DALYs) associated with depression were sourced from the GBD 2019. This extensive dataset is accessible online at “http://ghdx.healthdata.org/gbd-results-tool,” hosted by the Institute for Health Metrics and Evaluation. The methodologies employed in the GBD 2019 study have been detailed in prior publications [9, 10]. Essentially, the GBD study employs sophisticated statistical modeling to leverage a wide array of epidemiological data, encompassing both published and unpublished sources, as well as routinely gathered data such as vital records, hospitalization statistics, and medical claims. This approach is inclusive of data from within and just beyond the targeted country, region, or state, ensuring the most accurate disease burden estimates even in areas lacking precise epidemiological data.

In our research, we examined and evaluated extensive repeated cross-sectional data collected over a period of 30 years. This comprehensive dataset focused on various aspects of depression, including its incidence, prevalence, and DALYs. The DALYs approach was used as a methodology in this research, and the incidence-based DALYs approach was measured by totaling the values corresponding to years lived with disability and years of life lost. One DALY indicates that 1 year of full health was lost to disease, disability, and early death; as DALYs increase, the gap with the ideal health level widens, which suggests an increase in disease burden [11]. A key feature of our analysis was the categorization of this data based on different demographics such as sex, age, and geographical distinctions like region and country. For each category, we provided detailed figures along with age-standardized rates (ASRs). These ASRs, essential for understanding the impact of depression across different populations, were calculated per 100,000 individuals. Each statistical measure was also accompanied by a 95% uncertainty interval (UI), ensuring a comprehensive and nuanced understanding of the data. The specific methodology for calculating the ASR involved the following formula:

(1) $$\begin{array}{c} \text{ASR}=\frac{\sum_{i=1}^{A}a_{i}w_{i}}{\sum_{i=1}^{A}w_{i}}\times 100,000, \end{array}$$

where *a*_i_ is the age-specific rate in i^th^ age group; w is the number of people in the corresponding i^th^ age group among the standard population; and *A* is the number of age groups.

To further elucidate, the age-standardized incidence rate (ASIR) quantifies the incidence of new cases of a condition per 100,000 individuals, factoring in age distribution. Similarly, the age-standardized prevalence rate (ASPR) measures the total number of existing cases per 100,000 individuals, also adjusted for age differences. On the other hand, the age-standardized DALYs' rate (ASDR) gauges the burden of living with a disability. It is expressed as the number of years lived with disability per 100,000 individuals, once again adjusted for age.

The 95% UI provides a range of values that signify the level of certainty associated with these estimates. This interval is derived from the 25th and 75th ranked values of 1,000 different simulations of the posterior distribution, offering a robust statistical perspective.

To analyze the changes over time in incidence, prevalence, and DALYs, we computed the estimated annual percentage change (EAPC) rates. The EAPC is widely used in epidemiological research to track changes in ASRs of diseases. The natural logarithm of ASR has a linear relationship with time; hence,

(2) $$\begin{array}{c} Y=\alpha +\beta x+\varepsilon , \end{array}$$

where *Y* is ASR, x is calendar year, and *ε* is the error term. In this formula, *β* indicates the positive or negative trend in ASR. EAPC was calculated as follows:

(3) $$\begin{array}{c} \text{EAPC}=100\times \left(\exp\left(\beta \right)-1\right). \end{array}$$

The formula of EAPC and its 95% confidence interval (CI) were obtained using a linear model. When the lower limit of CI and the EAPC was positive, ASR was considered to have an upward trend. Conversely, when the upper limit of the CI and the EAPC was negative, the ASR was considered to have a downward trend.

Depression includes major depressive disorder (MDD) and dysthymia in the GBD study [12]. MDD is an episodic mood disorder involving the experience of one or more major depressive episode(s). Dysthymia is a mood disorder consisting of chronic depression, with less severe but longer lasting symptoms than MDD [12]. Included in the GBD study disease model were cases meeting the diagnostic criteria for MDD and dysthymia according to the Diagnostic and Statistical Manual (DSM) and International Classification of Diseases (ICD).

### 2.2. Sociodemographic Index

In our study, we also meticulously collected data on the sociodemographic index (SDI) for each country and territory. The SDI is a composite indicator, meticulously constructed from three fundamental components: per capita income, the average level of education attained by individuals aged 15 years and older, and the total fertility rate among females under 25 years of age. This composite metric offers a multifaceted perspective on the socioeconomic and demographic attributes of a region.

For the GBD study conducted in 2019, countries and territories were categorized into five distinct SDI tiers. These are low (SDI less than 0.46), low–middle (SDI between 0.46 and 0.60), middle (SDI between 0.61 and 0.69), high–middle (SDI between 0.70 and 0.81), and high (SDI greater than 0.81). Such a stratification allows for a more detailed and comprehensive analysis of the socioeconomic and demographic factors influencing various health outcomes. By integrating the SDI into our analysis, we can gain a deeper understanding of the relationship between sociodemographic factors and the prevalence, incidence, and burden of diseases, including depression, across different global regions.

### 2.3. Age-Period-Cohort Analysis

In our study, we utilized the age–period–cohort (APC) model to meticulously analyze the underlying trends, focusing on variations by age, time period, and birth cohort [10]. The APC model is a sophisticated tool that goes beyond the scope of traditional epidemiological analysis. It is specifically engineered to dissect and understand how age-related biological factors, as well as technological and social influences, affect disease trends. This model has been previously employed in descriptive epidemiological studies of certain chronic cardiovascular diseases, demonstrating its applicability and effectiveness in such contexts [13].

The APC model operates by fitting a log-linear Poisson model across a Lexis diagram, which graphically represents observed rates. It calculates the additive effects of three critical variables: age, period, and birth cohort. One of the inherent challenges in applying the APC model is the “identification problem.” This arises due to the perfectly linear relationship among age, period, and cohort (with birth cohort being equivalent to period minus age), which makes it statistically challenging to isolate their independent effects [14, 15]. To circumvent this problem, we here produced estimable APC parameters and functions without imposing arbitrary constraints on the model [10]. The details in the methodology of the APC model have been thoroughly discussed elsewhere [16]. This approach ensures more accurate and reliable results. The construction of our APC model was carried out using open-source tools available in R version 4.2.2., and the specifics of this process have been detailed in other publications. By applying this model, we were able to gain a deeper and more nuanced understanding of the trends associated with various age groups, time periods, and generational cohorts, offering valuable insights into the evolving nature of disease patterns.

We incorporated data on depression and applied the APC model to identify underlying patterns and trends. To achieve precision in our investigation, we organized the data into 5-year age intervals. These intervals were aligned with corresponding 5-year periods, creating a comprehensive timeline from 1990 to 2019. This span was divided into six distinct segments for detailed analysis: 1990–1994, 1995–1999, 2000–2004, 2005–2009, 2010–2014, and 2015–2019. Furthermore, we constructed eight overlapping 10-year birth cohorts, which ranged from individuals born in 1965 to those born in 2009. This strategic organization of data into age intervals, periods, and birth cohorts enabled a comprehensive examination of the influence of these factors on the trends of depression. This approach provided an insightful perspective on how depression patterns have evolved over time across different generations. In our APC model, the age effects are observed through the analysis of age-specific rates across various birth cohorts, with adjustments made for period influences. Meanwhile, the period and cohort effects are interpreted as relative risks of the disease burden. This is achieved by comparing age-specific rates across different periods or cohorts against a selected reference point. It's important to note that the choice of this reference point is arbitrary and does not impact the overall interpretability of the results. To assess the statistical significance of the observed trends, we employed the Wald *χ*^2^ test. This test is a crucial component in our analysis, providing a robust statistical method to validate the significance of the trends identified in the APC model. The combination of these methodological approaches allowed us to draw meaningful conclusions about the changing patterns of depression, reflecting how demographic, temporal, and generational shifts impact its prevalence and characteristics.

### 2.4. Decomposition Analysis

Our study conducted decomposition analyses to understand the factors affecting the depression burden from 1990 to 2019. This involved examining changes in population size, age distribution, and epidemiological trends [17]. The methodologies for these analyses are detailed in a previously published article, which provides an in-depth reference for the techniques used [17].

## 3. Results

### 3.1. Global Trends

**Figure 1:**
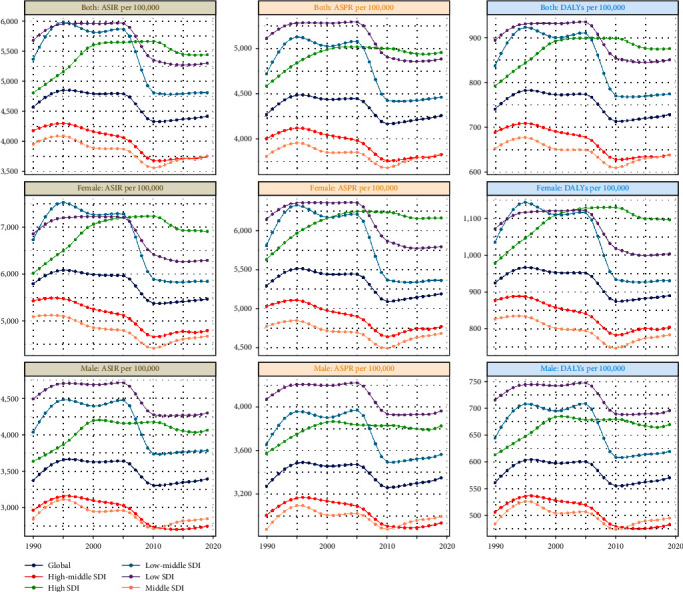
The trends in global and five SDI burdens of depression among 15–49 years from 1990 to 2019.

Overall, there were 115,820,137.2 estimated prevalent cases of depression in working-age individuals (95% UI 100,465,986.1–134,835,291.8) in 1990 and 167,691,041.9 prevalent cases (95% UI 145,875,786.5–194,382,493.8) in 2019, with an increase of 44.79% from 1990 to 2019 (Table 1 and Figure 1). ASPR dropped from 4,270.3 per 100,000 in 1990 to 4,261.4 per 100,000 in 2019 (EAPC = −0.22, 95% CI −0.3 to −0.14). The ASPR ratio of men and women shows a gradual upward trend on the whole, and in the world, the ASPR ratio of men and women reaches its maximum at the age of 30–34 (*Supplementary figure 1*). Additionally, depression accounted for 123,939,339.5 estimated incident cases (95% UI 104,436,519.8–146,787,731.8) in 1990 and 173,927,508.9 cases (95% UI 145,180,078.1–204,945,736.6) in 2019 (*Supplementary table 2* and Figure 1). The ASIR decreased between 1990 and 2019, with an EAPC of −0.4 (95% CI −0.52 to −0.28). Worldwide, the proportion of ASIR in males and females gradually increased and then gradually decreased, and the proportion of ASIR in males and females reached its maximum at 25–29 years of age (*Supplementary figure 3*). The number of DALYs increased from 200,938,72.9 (95% UI 13,739,547.3–27,905,974.8) in 1990 to 28,679,797.1 (95% UI 19,599,904.5–39,765,816.9) in 2019 (*Supplementary table 2* and Figure 1). The ASDR decreased between 1990 and 2019, with an EAPC of −0.3 (95% CI −0.4 to −0.21). The proportion of ASDR in both men and women reaches its maximum at ages 30–34 but is less than one overall (*Supplementary figure 4*).

### 3.2. Sociodemographic Index Region Level

Compared to 1990, in 2019, the prevalence, incidence, and DALYs of five SDIs have increased significantly in 2019 (Table 1 and *Supplementary table 2*). Among the five regions, only the EAPC of the high SDI region is greater than 0, and the EAPC of the other four SDI regions is less than 0. In 2019, high SDI had the highest ASPR (4,955.8 per 100,000; 95% UI: 4,355.3−5,690.9), ASIR (5,439.4 per 100,000; 95% UI: 4,637.4–6,395.7), and ASDR (875.8 per 100,000; 95% UI: 601.6–1209.1; Table 1 and *Supplementary table 2*). We found that among the five SDI regions, the ratio of male to female ASPR in the high SDI region reached its maximum at age 40–44. In the high–middle SDI, the middle SDI reaches its maximum age between 25 and 34 (*Supplementary figures 1, 3, and 4*). Similarly, we found that in 1990 and 2019, the prevalence, incidence, and DALYs of females at the same age were higher than those of males (*Supplementary figures 5, 6, and 7*).

### 3.3. Regional Trends

The GBD regional classification system, which encompasses 204 countries and territories segmented into 21 regions, reveals a significant disparity in adolescent depression prevalence and incidence across these regions in 2019. Notably, South Asia reported remarkably high numbers, with 41,900,779 cases in prevalence (95% UI: 36,648,758.1–48,574,218) and 45,222,545.2 cases in incidence (95% UI: 37,706,551.5–53,288,150.9). From 1990 to 2019, both the ASPR and the ASIR showed a declining trend, evidenced by EAPC of −0.84 (95% CI −1.06 to −0.61) and −1.23 (95% CI −1.54 to −0.91), respectively.

In contrast, Central sub-Saharan Africa and Australasia stood out with markedly higher rates across all three measures compared to other regions. This finding underscores the need for targeted interventions and resource allocation in these areas to address the disproportionate burden of disease. Notably, Central Latin America experienced the steepest rise in both ASPR (EAPC = 0.57; 95% CI 0.55–0.59) and ASIR (EAPC = 0.69; 95% CI 0.66–0.72). This rapid increase warrants urgent attention from policymakers and health authorities in the region. Implementing evidence-based strategies, improving healthcare access, and strengthening disease surveillance systems should be prioritized to curb this alarming trend. On the other hand, several regions, including East Asia, the Caribbean, Tropical Latin America, Southern Latin America, Eastern sub-Saharan Africa, Central Europe, and Western sub-Saharan Africa, saw slight decreases in ASPR (Table 1) and ASIR (*Supplementary table 2*). While this is an encouraging sign, it is crucial to maintain and intensify efforts in these regions to sustain the progress made in any potential resurgence.

The observed regional disparities in disease burden highlight the importance of tailoring global health policies to address region-specific needs. Policymakers should prioritize the development and implementation of comprehensive strategies that encompass early detection and effective management of the condition. This may involve strengthening primary healthcare systems; promoting health education; and fostering collaborations between governments, international organizations, and local communities. Furthermore, the findings emphasize the need for continued research to better understand the underlying factors contributing to the regional variations in disease burden. Identifying the social, economic, environmental, and cultural determinants of health in each region can inform the development of more targeted and effective interventions.

### 3.4. National Trends

**Figure 2:**
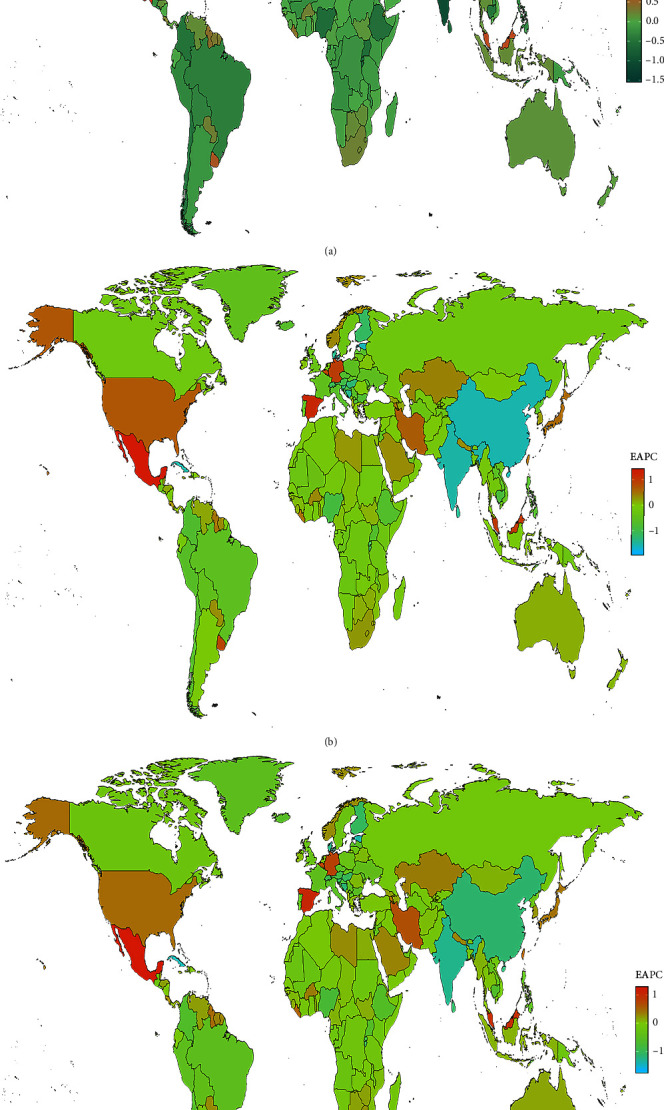
The EAPC for depression among 15–49 years prevalence (a), incidence (b), and DALYs (c) for both sexes in 204 countries and territories.

**Figure 3:**
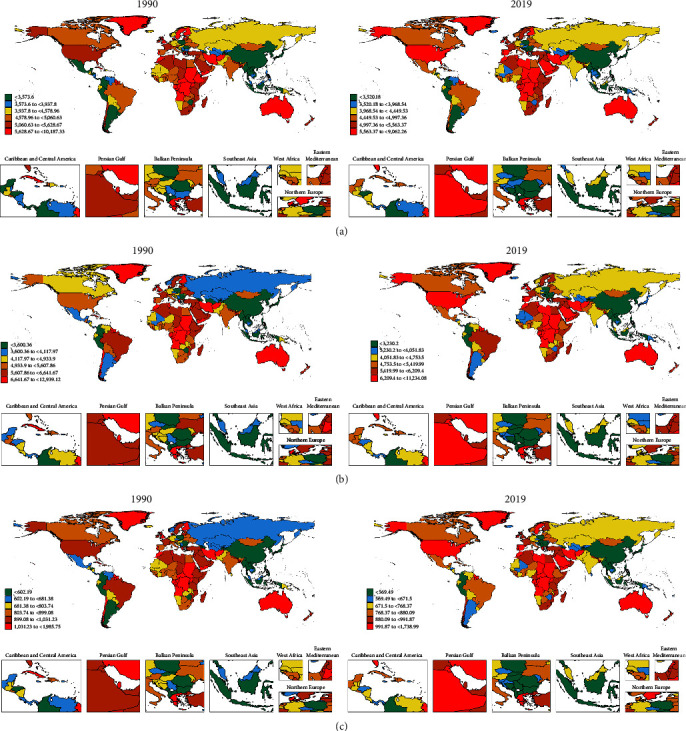
The maps showing (a) age-standardized prevalence rate, (b) age-standardized incidence rate, and (c) age-standardized DALYs' rate of depression in 204 countries and territories between 1990 and 2019.

**Figure 4:**
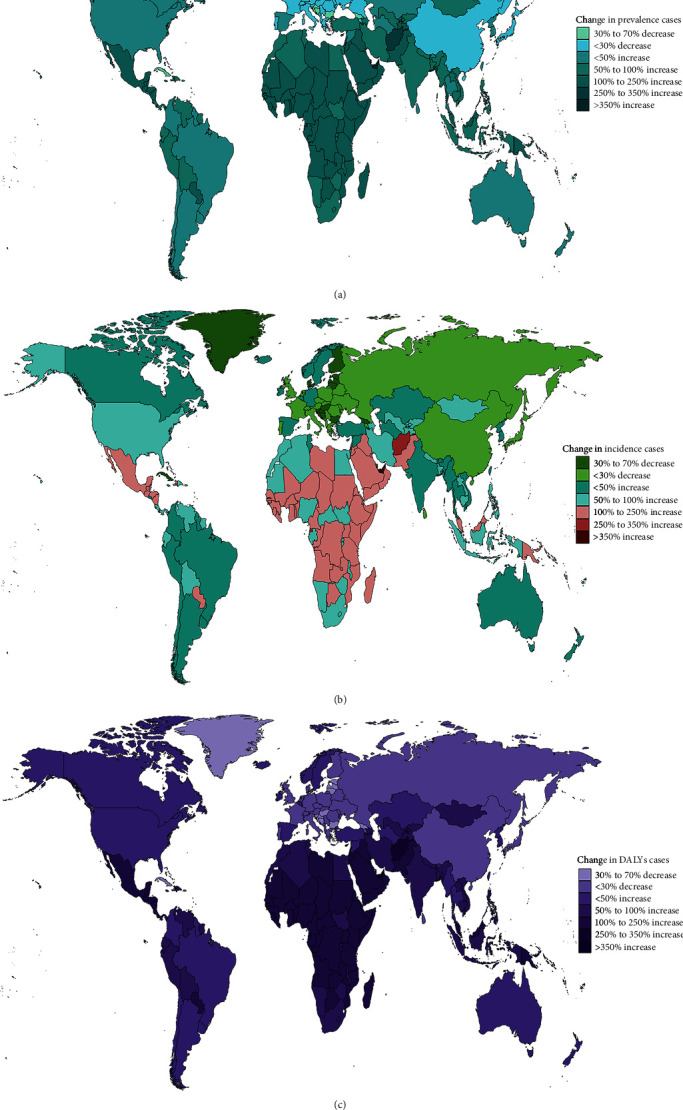
Change cases of depression for both sexes in 204 countries and territories. (a) Change in prevalence cases. (b) Change in incidence cases. (c) Change in DALYs cases.

In 2019, India, China, and the United States recorded the highest prevalence of depression cases among all countries, with India leading at 31,424,840.5 cases (95% UI: 27,559,116.4–36,289,659.5; EAPC: −1.05; and 95% CI: −1.33 to −0.76), followed by China with 22,319,553.4 cases (95% UI: 19,543,443.3–25,476,524.2; EAPC: −0.73; and 95% CI: −0.85 to −0.62), and the United States with 9,599,583.6 cases (95% UI: 8,503,099.8–10,899,310.5; EAPC: 0.26; and 95% CI: 0.08–0.43) as shown in *Supplementary table 2* and Figure 2. In terms of ASPR, Yemen reported the highest rate with 9,062.3 cases per 100,000 individuals (95% UI: 7,690.9–10,710.8), closely followed by Angola (7,792.9 per 100,000; 95% UI: 6,462.9–9,392.2), and the United States (7,436.2 per 100,000; 95% UI: 6,144.1–8,870.1; *Supplementary table 2* and Figure 3(a)). Qatar experienced the most significant increase in prevalence cases, surging by 595.03% from 16,577.3 cases in 1990 to 115,217.8 in 2019 (Figure 4). Similarly, in 2019, India, the United States, and China also had the highest incidence of adolescent depression cases. India reported 33,277,196.5 cases (95% UI: 27,928,639.3–39,352,621.6), the United States had 10,867,823.4 cases (95% UI: 9,331,179.9–12,667,233.1), and China recorded 17,943,053.6 cases (95% UI: 15,225,607.2–20,896,343), as indicated in *Supplementary table 2*. For the ASIR, Colombia had the highest rate with 11,234.1 per 100,000 individuals (95% UI: 9,072.7–13,633.6), followed by Niger (9,912.6 per 100,000; 95% UI: 7,852.3–12,444), and Palestine (9,155.7 per 100,000; 95% UI: 7,214–11,335.9; *Supplementary table 2* and Figure 3(b)). Qatar witnessed the most significant increase in incident cases, a 578.7% rise from 19,502.9 in 1990 to 132,376.5 in 2019 (Figure 4). The DALYs of depression in China has dropped from 3,961,460.3 in 1990 to 3,415,496.5 in 2019 (EAPC: −1.13; 95%CI:−1.28 to −0.97; *Supplementary table 2* and Figures 2 and 3(c)).

### 3.5. Age, Period, and Cohort Effects on the Global Trend

**Figure 5:**
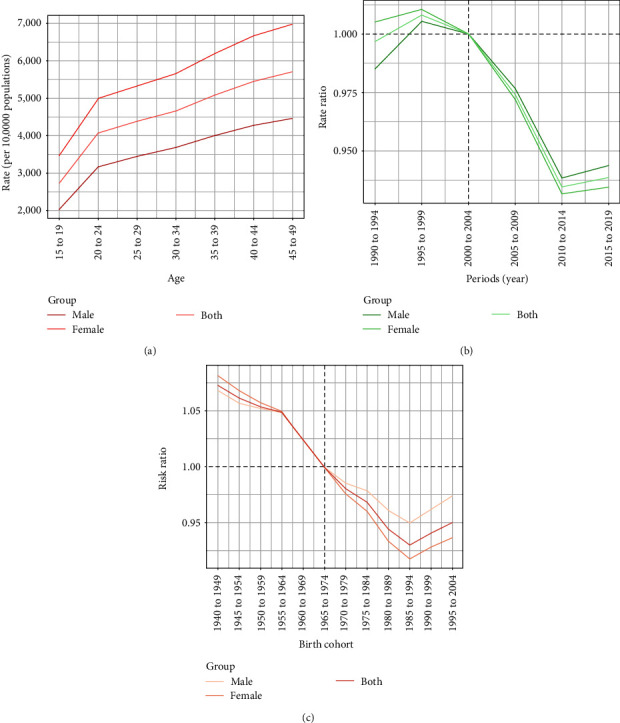
Age, period, and cohort effects on prevalence rates of depression by sex from 1990 to 2019. (a) Change in prevalence cases. (b) Change in incidence cases. (c) Change in DALYs cases.

Between 1990 and 2019, there was a decreasing prevalence risk with age overall (net drift −0.32; 95% CI −0.36 to −0.28) in females (net drift −0.36; 95% CI −0.41 to −0.32) and in males (net drift −0.25; 95% CI −0.29 to −0.22; *Supplementary table 2*). The risk of depression prevalence was highest in those aged 45–49 years for both females and males (Figure 5(a)).

This age group shows the most substantial impact concerning both the incidence of depression and the DALYs attributed to it (*Supplementary figures 8 and 9*).

Period effects generally showed a declining risk of depression incidence, prevalence, and DALYs over the period and in both sexes (Figure 5(b) and *Supplementary figures 8 and 9*). Compared with the reference period of 2000–2004, the period 1995–1999 had the highest period risk for the incidence, prevalence, and DALYs' rates regardless of gender (Figure 5(b) and *Supplementary figures 8 and 9*).

In the 12 consecutive 10-year birth cohorts from 1940 to 2004, the cohort risk for the depression incidence, prevalence, and DALYs' rates showed a decline followed by an increase and increased in the successive cohorts since 1985–1994 (Figure 5(c) and *Supplementary figures 8 and 9*). Compared with the 1965–1974 birth cohort, the 1940–1949 cohorts had the highest cohort risk for the incidence, prevalence, and DALYs' rates regardless of gender (Figure 5(c) and *Supplementary figures 8 and 9*).

### 3.6. Decomposition Analysis

Our decomposition analysis provided insights into the relative contributions of aging, population, and demographically adjusted changes of epidemiology in DALYs of depression according to five SDI regions and 21 GBD regions. We found that the increase in depression DALYs globally and in five SDI regions was mainly attributable to population factors (*Supplementary figure 10*). In addition, population growth has a significantly higher effect on the burden of depression in women than in men. Epidemiological change has been cited as a boost in high SDI areas, while it has been shown to ease the burden of depression in the remaining SDI areas.

## 4. Discussion

This comprehensive analysis of GBD data provides key insights into the global, regional, and national epidemiology of depression among working-age adults from 1990 to 2019. Our study reveals that the overall estimated number of depression cases and the associated disease burden increased. However, it is noteworthy that socioeconomic progress has contributed to a decrease in depression rates. Peak prevalence occurred in midlife, underscoring the significance of depression onset compromising individuals during their prime working years.

Although the total estimated cases and DALYs from depression increased by 44.79% and 42.72% from 1990 to 2019, respectively, ASRs measuring the burden adjusted for population aging declined slightly (ASPR EAPC = −0.22; ASIR EAPC = −0.4; ASDR EAPC = −0.3). This divergence reflects global population growth outweighing modest epidemiological improvements, as highlighted in our decomposition analysis. Still, these declining standardized rates indicate progress in mitigating depression even amidst expanding and aging populations.

The peak depression prevalence risk occurring at ages 45–49 years aligns with prior research showing typical disease onset concentrated in midlife adulthood [18]. This trajectory differs from conditions with childhood–adolescent onset, such as certain anxiety disorders. The midlife peak underscores the significant impact of depression on individuals during critical working and child-rearing years, resulting in lost productivity and functional impairments that incur heavy societal costs [19, 20].

Geographical patterns demonstrate the complex interplay of socioeconomic advancement and epidemiological factors influencing depression burden across settings. While higher SDI regions had the greatest total prevalence, incidence, and DALYs in 2019, they uniquely showed rising standardized rates over time (2022). By comparison, other SDI regions displayed declining age-adjusted rates from 1990 to 2019. Regional variations also emerged: Central Latin America experienced rapid standardized rate escalations from 1990 to 2019, whereas South Asia saw declines despite high total prevalence. National figures similarly demonstrated wide variability [21]. These divergent trends likely reflect the multifaceted nature of social development and its impact on mental health. Higher income regions generally possess greater resources for depression assessment and care. Their advanced health systems enable better detection through improved access to medical infrastructure for diagnosis along with reduced stigma barriers to help-seeking [22, 23]. Standardized disease metrics would naturally rise as more cases get identified and documented. However, increased detection alone may not fully explain the observed trends, and several other factors should be considered [23]. Firstly, rapid urbanization and modernization in higher SDI regions may contribute to increased stress levels and social isolation, which are known risk factors for depression [24, 25]. Changes in lifestyle, such as reduced physical activity and altered dietary patterns, could also play a role [26]. Additionally, the erosion of traditional social support systems and the challenges of adapting to new sociocultural norms in the face of globalization may exacerbate mental health issues [27]. Secondly, the increased prevalence of chronic diseases in higher SDI regions, such as cardiovascular disorders and diabetes, may also contribute to the rising burden of depression [28]. Comorbidities between physical and mental health conditions are well established, and the management of chronic diseases can take a toll on an individual's mental well-being [29].

Our APC analysis provides additional insights. Declining period effects over recent decades, especially among females, indicate modest optimism around temporally linked factors influencing depression patterns. However, unfavorable cohort patterns in those born after 1985 are concerning successive generations that appear more vulnerable to depression compared to predecessors at similar ages [30, 31, 32]. Various hypotheses, from digital technology exposures to thinning social connectedness among youth, may underpin these generational shifts [33, 34]. Ongoing monitoring through future GBD studies will clarify if these early-life risk elevations endure over the lifespan.

The strength of this study is providing an up-to-date analysis of global epidemiological trends in working-age adult depression based on the comprehensive GBD 2019 dataset. It includes multidimensional measures of disease burden (prevalence, incidence, and DALYs) while examining changes across global, regional, and national levels over 1990–2019. The application of APC statistical modeling also enables estimates of the independent effects of age, time periods, and birth cohorts on depression patterns. This offers a nuanced, longitudinal perspective spanning 30 years not found in most mental health data.

However, certain limitations inherent to GBD approaches should be considered regarding depression specifics. For example, diagnostic criteria aligned predominantly with DSM-IV-TR and ICD-10 classifications, but their cross-cultural consistency is uncertain. Emerging nosologies like DSM-5 also require assessing impacts on estimates. Potential data source biases and variable instrumentation affecting case ascertainment pose additional challenges measuring a complex condition like depression. Furthermore, the omission of data on depression subtypes or comorbid conditions limits the scope of the results. Excluding comorbidity details with related illnesses like cardiovascular disease also restricts understanding integrated care needs when conditions co-occur. Still, by compiling an extensive array of data sources, the GBD makes great strides in quantifying global disease burden. Ongoing refinements will further strengthen these valuable tools guiding policy and practice.

It is important to note that the publication of the DSM-5 introduced changes to the diagnostic criteria for depressive disorders, which may impact the epidemiological estimates. Studies conducted after the release of DSM-5 may yield varying results compared to those based on the previous DSM-IV-TR criteria. As more data become available using the updated diagnostic framework, it will be crucial to assess the potential implications on the burden estimates for depression. Ongoing efforts to incorporate the latest diagnostic criteria and data sources will be essential for ensuring the accuracy and relevance of the GBD estimates.

## 5. Conclusions

This far-reaching analysis of GBD 2019 data offers a rigorous profile of depression patterns among working-age adults worldwide over the past three decades. Encouraging declines in age-standardized indicators likely reflect strengthening economic progression and social modernization enabling healthier mental health trajectories across populations. However, the sheer rise in raw cases amidst population pressures remains concerning, especially surrounding the identified midlife peak. Moreover, generational increases among youth cohorts demand urgent attention. Redoubling targeted prevention interventions in cost-efficient platforms aligned to different age-risk profiles and sociodemographic factors will prove key to sustaining gains made while bending projected trends. As the field continues rallying around the depression burden, these findings combined with ongoing surveillance highlight actionable priorities on the road ahead.

## Figures and Tables

**Table 1:** Prevalence of depression in working-age individuals between 1990 and 2019.

Location	1990		2019		
	Number	ASPR	Number	ASPR	EAPC
Global	115,820,137.2 (100,465,986.1–134,835,291.8)	4,270.3 (3,704.2–4,971.4)	167,691,041.9 (145,875,786.5–194,382,493.8)	4,261.4 (3,707–4,939.7)	−0.22 (−0.3 to 0.14)
High SDI	19,727,951.9 (17,444,593.4–22,481,210.6)	4,587.1 (4,056.2–5,227.3)	23,288,458.4 (20,466,307.1–26,742,604.7)	4,955.8 (4,355.3–5,690.9)	0.18 (0.1–0.26)
High–middle SDI	24,237,291.9 (21,118,314–28,116,823.7)	4,004.4 (3,489.1–4,645.3)	27,912,359.5 (24,366,866.6–32,038,685.4)	3,831.4 (3,344.7–4,397.8)	−0.34 (−0.41 to 0.27)
Middle SDI	34,401,178.8 (29,856,533.5–39,964,513.6)	3,812.2 (3,308.6–4,428.7)	48,354,986.4 (42,086,613.7–55,945,506.4)	3,835.7 (3,338.5–4,437.8)	−0.15 (−0.21 to 0.09)
Low–middle SDI	25,503,297.4 (21,948,997.1–29,879,342.8)	4,718.6 (4,061–5,528.3)	41,645,963.4 (36,052,432.2–48,427,405)	4,461.8 (3,862.6–5,188.4)	−0.56 (−0.71 to 0.4)
Low SDI	11,885,686 (10,076,458.5–13,979,250.8)	5,111.2 (4,333.2–6,011.5)	26,388,974.8 (22,394,393.7–30,944,140.1)	4,883.6 (4,144.4–5,726.6)	−0.34 (−0.43 to 0.25)
Andean Latin America	633,005 (536,882.1–760,945.8)	3,399.1 (2,882.9–4,086.1)	1,095,017.7 (922,086.8–1,317,058)	3,305.5 (2,783.5–3,975.8)	−0.16 (−0.2 to 0.13)
Australasia	651,132.6 (569,288.8–754,492.2)	6,033.8 (5,275.4–6,991.6)	846,109.1 (722,145.6–999,821.8)	6,256.5 (5,339.9–7,393.1)	0.13 (0.02–0.25)
Caribbean	905,539.1 (762,774.7–1,071,978.4)	4,964.7 (4,181.9–5,877.2)	1,072,026.4 (899,036.4–1,278,526)	4,483.2 (3,759.8–5,346.8)	−0.44 (−0.49 to 0.39)
Central Asia	1,216,297.6 (1,025,650.5–1,469,781)	3,645.5 (3,074.1–4,405.3)	1,823,019 (1,531,556.8–2,208,446.9)	3733.5 (3136.6–4522.8)	0.08 (0.05–0.11)
Central Europe	2,120,838.3 (1,820,535.8–2,512,589.1)	3,476.5 (2984.2–4,118.6)	1,755,900 (1,498,041.9–2,091,832.5)	3,330.9 (2,841.7–3,968.1)	−0.3 (−0.36 to 0.23)
Central Latin America	2,826,722.2 (2,419,855.9–3,298,796.2)	3,466.3 (2,967.4–4,045.2)	5281,105.4 (4,559,572.3–6,141,188.2)	4,009.7 (3,461.9–4,662.7)	0.57 (0.55–0.59)
Central sub-Saharan Africa	1,739,493.6 (1,436,568.8–2,092,471.2)	7,125.8 (5,884.9–8,571.8)	4,264,805.6 (3,532,750.7–5,120,677)	6,860.1 (5,682.6–8,236.8)	−0.18 (−0.2 to 0.15)
East Asia	24,215,585.9 (21,140,151.7–27,910,067)	3,507.5 (3,062.1–4,042.7)	23,115,852.7 (20,223,451.5–26,458,221)	3,097.3 (2,709.7–3,545.1)	−0.71 (−0.82 to 0.59)
Eastern Europe	4,751,977.2 (4,099,891.3–5,545,972)	4,307.4 (3,716.3–5,027.1)	4,223,990.6 (3,649,780.7–4,914,058.6)	4,307.4 (3,721.9–5,011.1)	−0.15 (−0.22 to 0.07)
Eastern sub-Saharan Africa	4,650,125.6 (3,937,638.6–5,479,187.1)	5,603.7 (4,745.1–6,602.7)	10,580,090.5 (8,976,730–12,512,610.4)	5,319 (4,512.9–6,290.5)	−0.3 (−0.34 to 0.25)
High-income Asia Pacific	2,539,862.8 (2,241,554.2–2,902,712.9)	2,734.3 (2,413.1–3,124.9)	2,284,204.8 (2,021,263.7–2,603,229.2)	2,815 (2,491–3,208.2)	0.19 (0.1–0.29)
High-income North America	8,071,242.1 (7,140,407.2–9,210,278.4)	5,430.1 (4,803.9–6,196.4)	10,403,945.7 (9,200,565.5–11,825,464.7)	6,239 (5,517.4–7,091.5)	0.23 (0.06–0.39)
North Africa and Middle East	8,993,482.4 (7,653,224–10,604,514.4)	5,537.5 (4,712.3–6,529.4)	19,053,609.4 (15,971,645.2–22,708,843.6)	5,710.5 (4,786.8–6,806)	0.15 (0.11–0.19)
Oceania	122,694.9 (102,030.2–147,693.5)	3,875.2 (3,222.5–4,664.7)	260,782.8 (216,576.7–313,742.9)	3,833.7 (3,183.8–4,612.2)	−0.07 (−0.08 to 0.06)
South Asia	25,000,029.5 (21,630,168.6–29,002,122.8)	4,723.1 (4,086.4–5,479.2)	41,900,779 (36,648,758.1–48,574,218)	4,301.3 (3,762.1–4,986.3)	−0.84 (−1.06 to 0.61)
Southeast Asia	7,367,765.4 (6,313,849.5–8,651,315)	3,116.7 (2,670.9–3,659.7)	11,503,675.9 (9,867,971.3–13,519,082.9)	3,177.2 (2,725.5–3,733.9)	0.04 (0.01–0.08)
Southern Latin America	985,283.6 (857,652.7–1,137,076.7)	4,022.5 (3,501.5–4,642.2)	1,278,307.8 (1,115,094.5–1,485,929.8)	3,755.7 (3,276.1–4,365.6)	−0.3 (−0.37 to 0.23)
Southern sub-Saharan Africa	1,257,031 (1,084,885.4–1,455,653.6)	4,818.4 (4,158.5–5,579.7)	2,119,144.2 (1,836,574.2–2,470,960.9)	5,009.3 (4341.3–5,840.9)	0.23 (0.13–0.34)
Tropical Latin America	3,885,756.9 (3,365,548.2–4,480,025)	4,947.7 (4,285.4–5,704.4)	5,574,431.9 (4,956,796.6–6,314,613.5)	4,676.3 (4,158.1–5,297.2)	−0.35 (−0.65 to 0.05)
Western Europe	9,828,944.3 (8,691,281.3–11,161,895)	5,081.6 (4,493.4–5,770.7)	9,654,696.2 (8,416,019.3–11,252,553.6)	5,062.6 (4,413.1–5,900.5)	0 (−0.02 to 0.01)
Western sub-Saharan Africa	4,057,327.2 (3,439,012.4–4,766,192.7)	4,764 (4,038–5,596.4)	9,599,547.1 (8,148,264.6–11,317,785.3)	4,465 (3,790–5,264.2)	−0.26 (−0.43 to 0.09)

## Data Availability

GBD study 2019 data resources are available online from the Global Health Data Exchange (GHDx) query tool (http://ghdx.healthdata.org/gbd-results-tool).

## Supplementary Materials

Supplementary 1 Figure 1: ratio of male to female prevalence of depression in different age subgroups.

Supplementary 2 Table 1: incidence of depression in working-age individuals between 1990 and 2019. Table 2: DALYs of depression in working-age individuals between 1990 and 2019. Table 3: prevalence of depression in working-age individuals between 1990 and 2019 in 204 countries. Table 4: incidence of depression in working-age individuals between 1990 and 2019 in 204 countries. Table 5: DALYs of depression in working-age individuals between 1990 and 2019 in 204 countries. Table 6: annual percentage change for depression prevalence overall (net drift) by sex from 1990 to 2019.

Supplementary 3 Figure 2: ratio of male to female incidence of depression in different age subgroups.

Supplementary 4 Figure 3: ratio of male to female DALYs of depression in different age subgroups.

Supplementary 5 Figure 4: prevalence of depression in five SDI regions by age group.

Supplementary 6 Figure 5: incidence of depression in five SDI regions by age group.

Supplementary 7 Figure 6: DALYs of depression in five SDI regions by age group.

Supplementary 8 Figure 7: age, period, and cohort effects on incidence rates of depression by sex from 1990 and 2019

Supplementary 9 Figure 8: age, period, and cohort effects on DALYs' rates of depression by sex from 1990 and 2019

Supplementary 10 Figure 9: decomposition analysis of depression aged 10–24 years for change in DALYs.
