# Corrigendum to “Global, Regional, and National Epidemiology of Depression in Working-Age Individuals, 1990–2019”

**DOI:** 10.1155/da/9761652

**Published:** 2025-05-29

J.-s. Yang, L.-y. Zhang, C.-h. Yang, X.-y. Li, and Z.-q. Li, “Global, Regional, and National Epidemiology of Depression in Working-Age Individuals, 1990–2019,” *Depression and Anxiety* 2024, no. 1 (2024): 1–14, https://doi.org/10.1155/2024/4747449.

In the article titled “Global, Regional, and National Epidemiology of Depression in Working-Age Individuals, 1990–2019,” the contact details for Zhi-qiang Li were incorrect. The correct contact information is shown below:

Correspondence should be addressed to Zhi-qiang Li; 361960023@qq.com

We apologize for this error.

