# Supplementary figures and images for: Global, Regional, and National Epidemiology of Depression in Working-Age Individuals, 1990–2019
Source: Depress Anxiety. 2024 Aug 24;2024:4747449. doi: 10.1155/2024/4747449 (PMC11919199; doi:10.1155/2024/4747449)

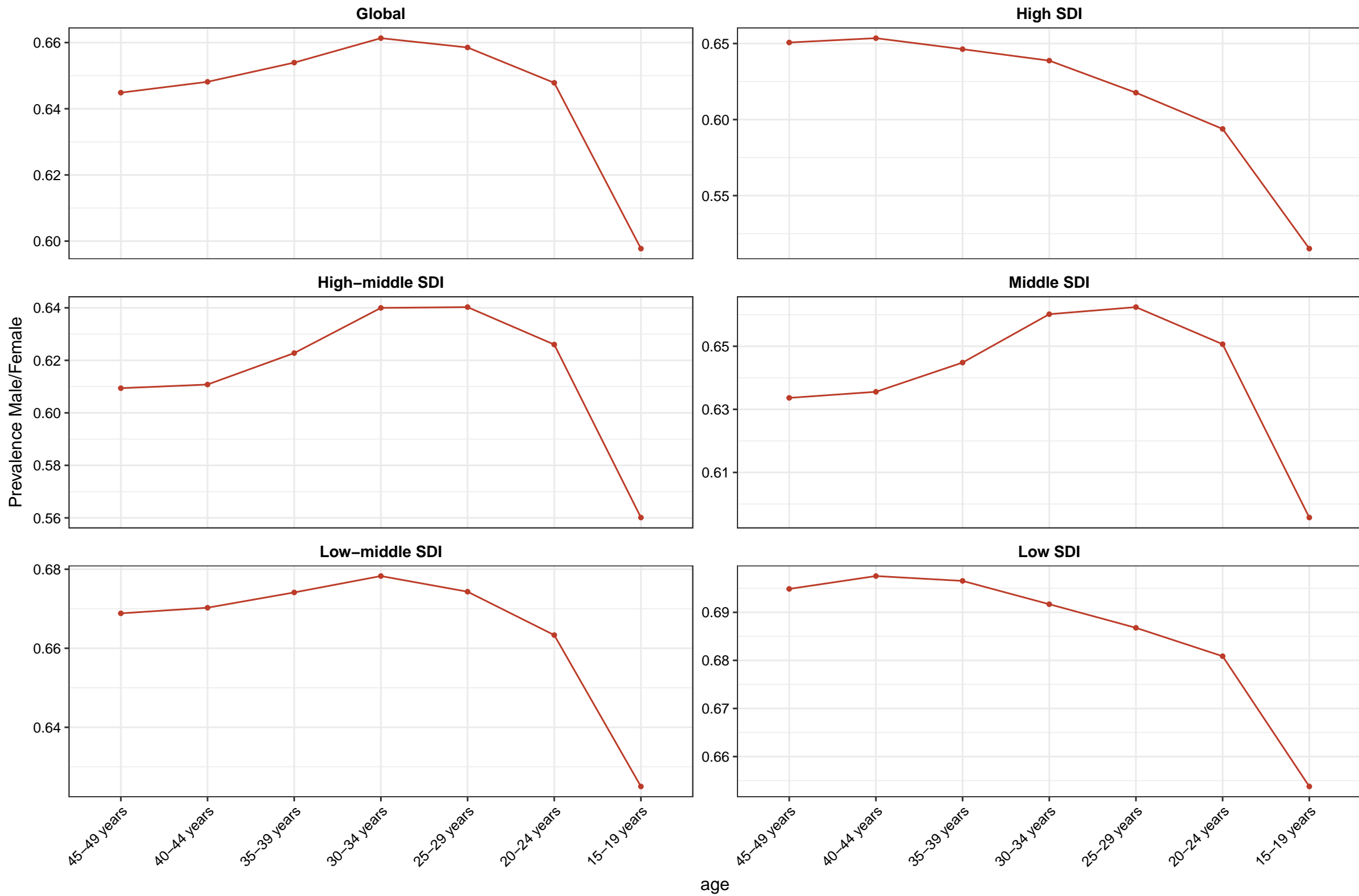

Supplement: Supplementary 1 — Figure 1: ratio of male to female prevalence of depression in different age subgroups. [file 4747449.f1.pdf]

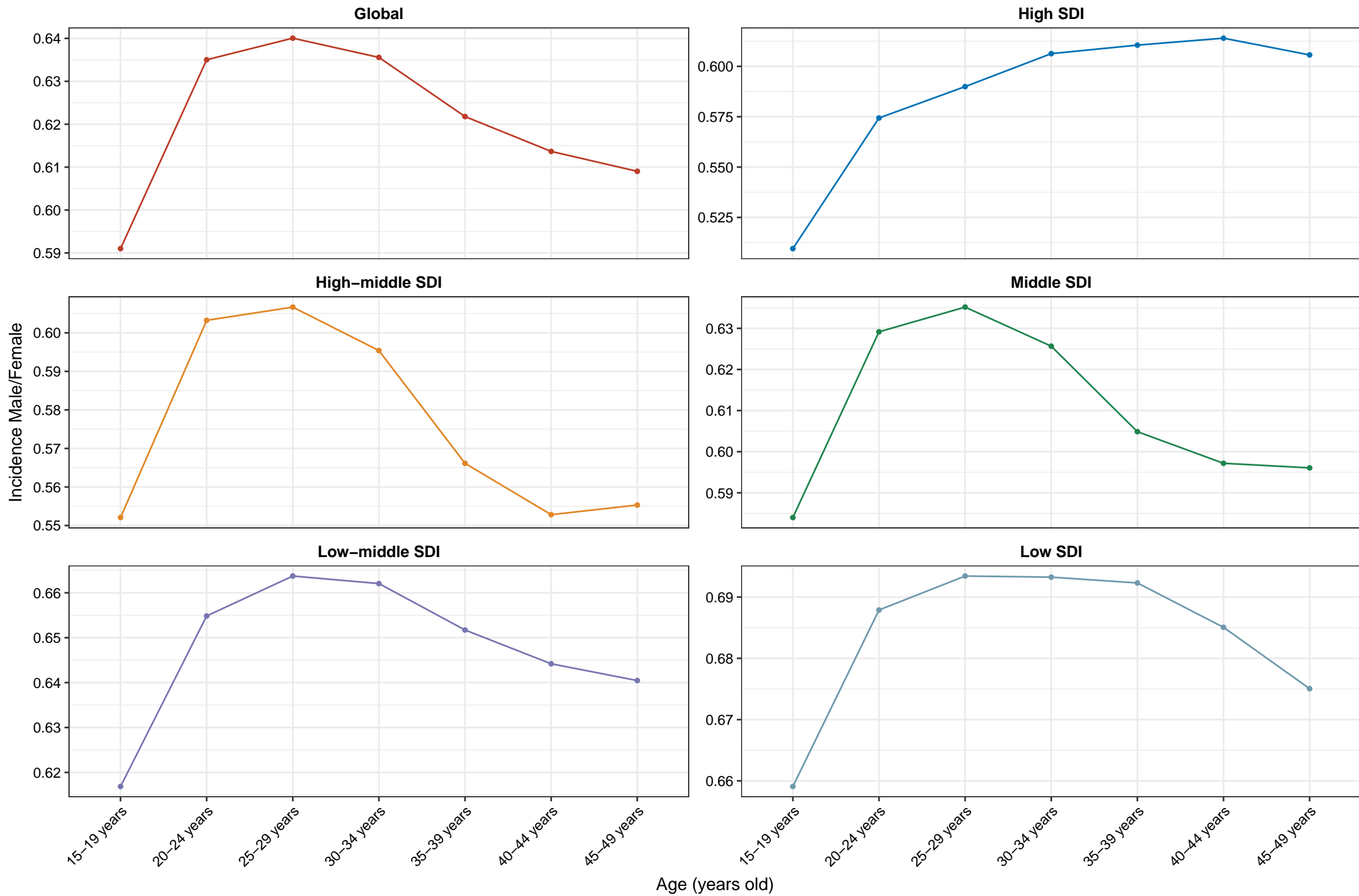

Supplement: Supplementary 3 — Figure 2: ratio of male to female incidence of depression in different age subgroups. [file 4747449.f3.pdf]

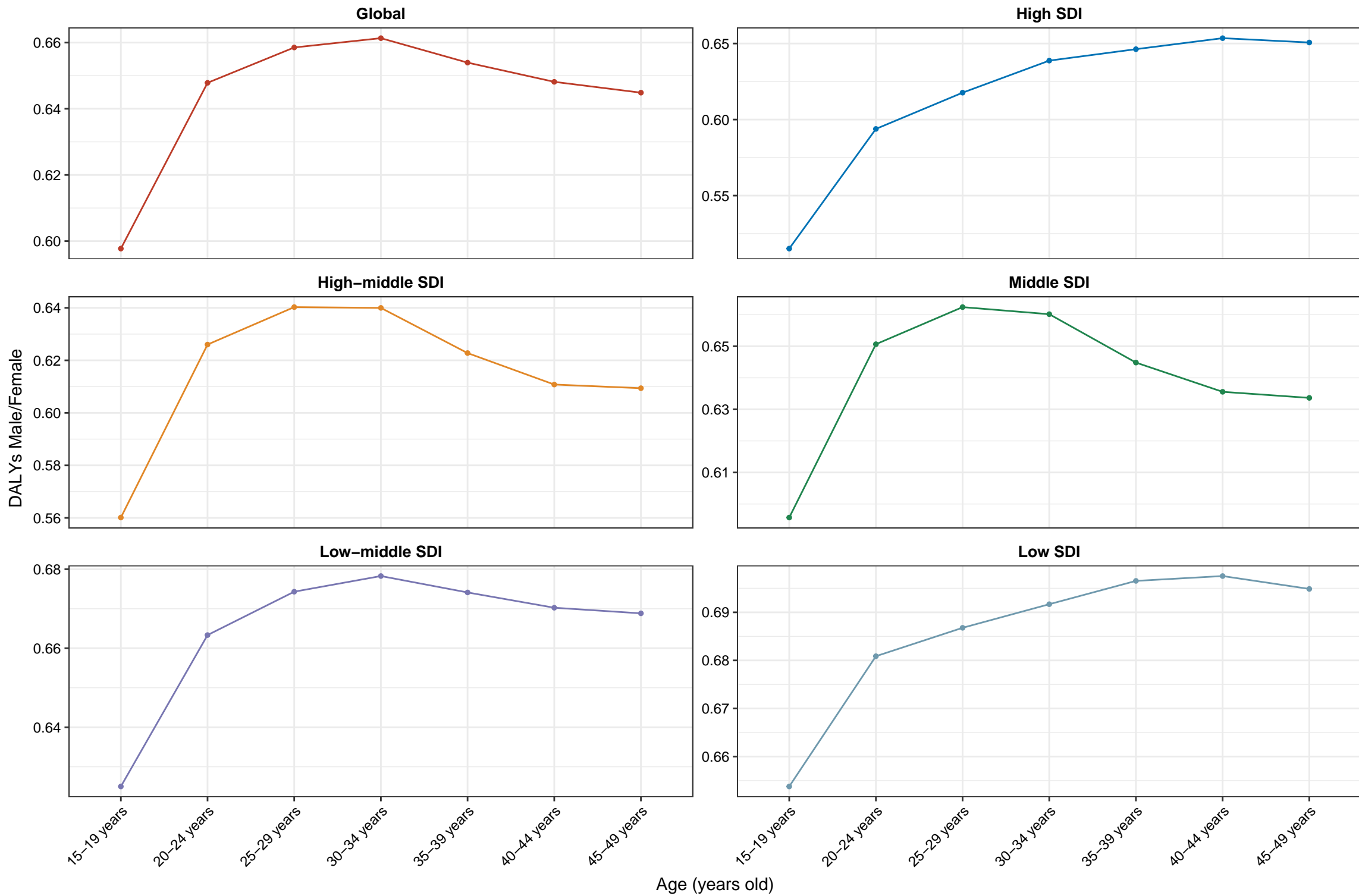

Supplement: Supplementary 4 — Figure 3: ratio of male to female DALYs of depression in different age subgroups. [file 4747449.f4.pdf]

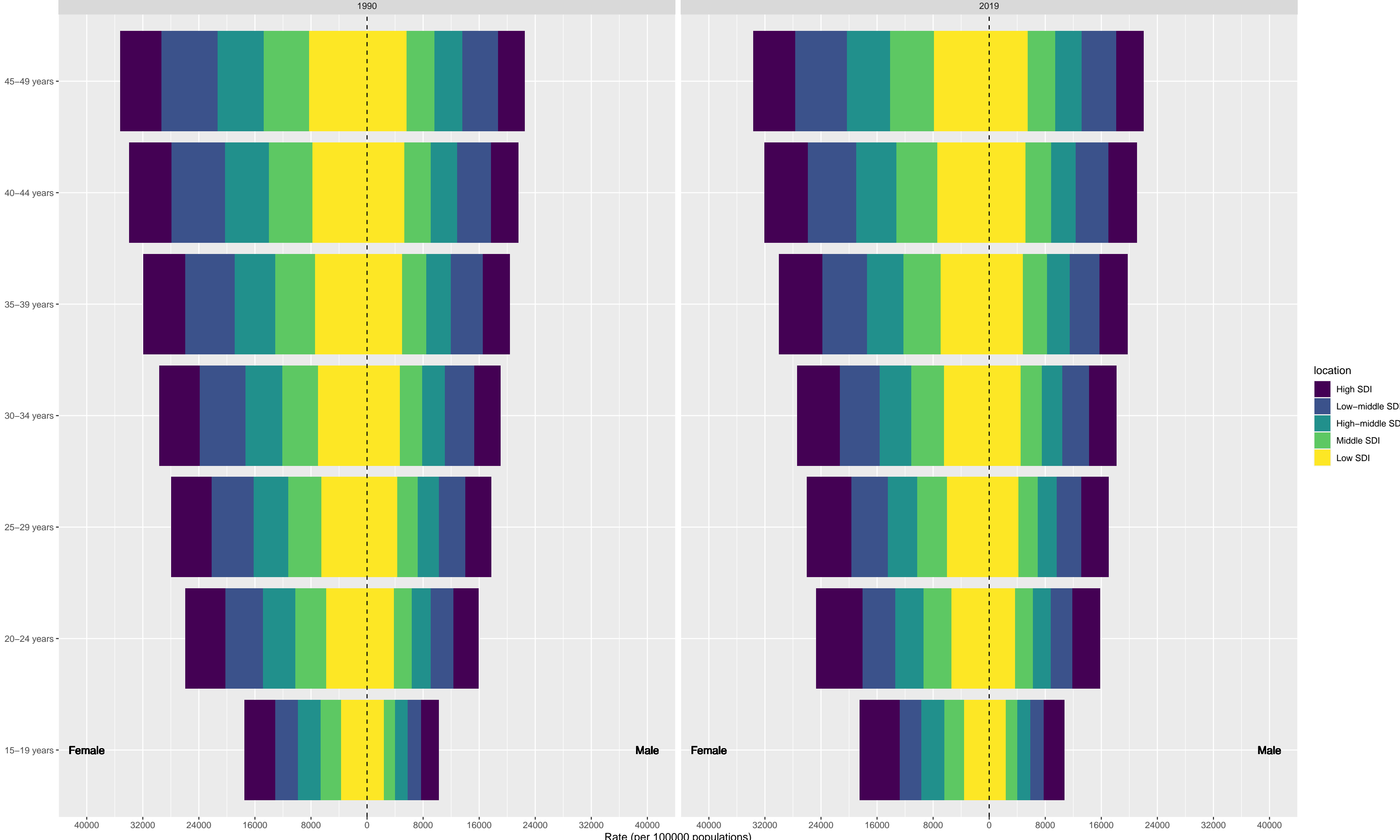

Supplement: Supplementary 5 — Figure 4: prevalence of depression in five SDI regions by age group. [file 4747449.f5.pdf]

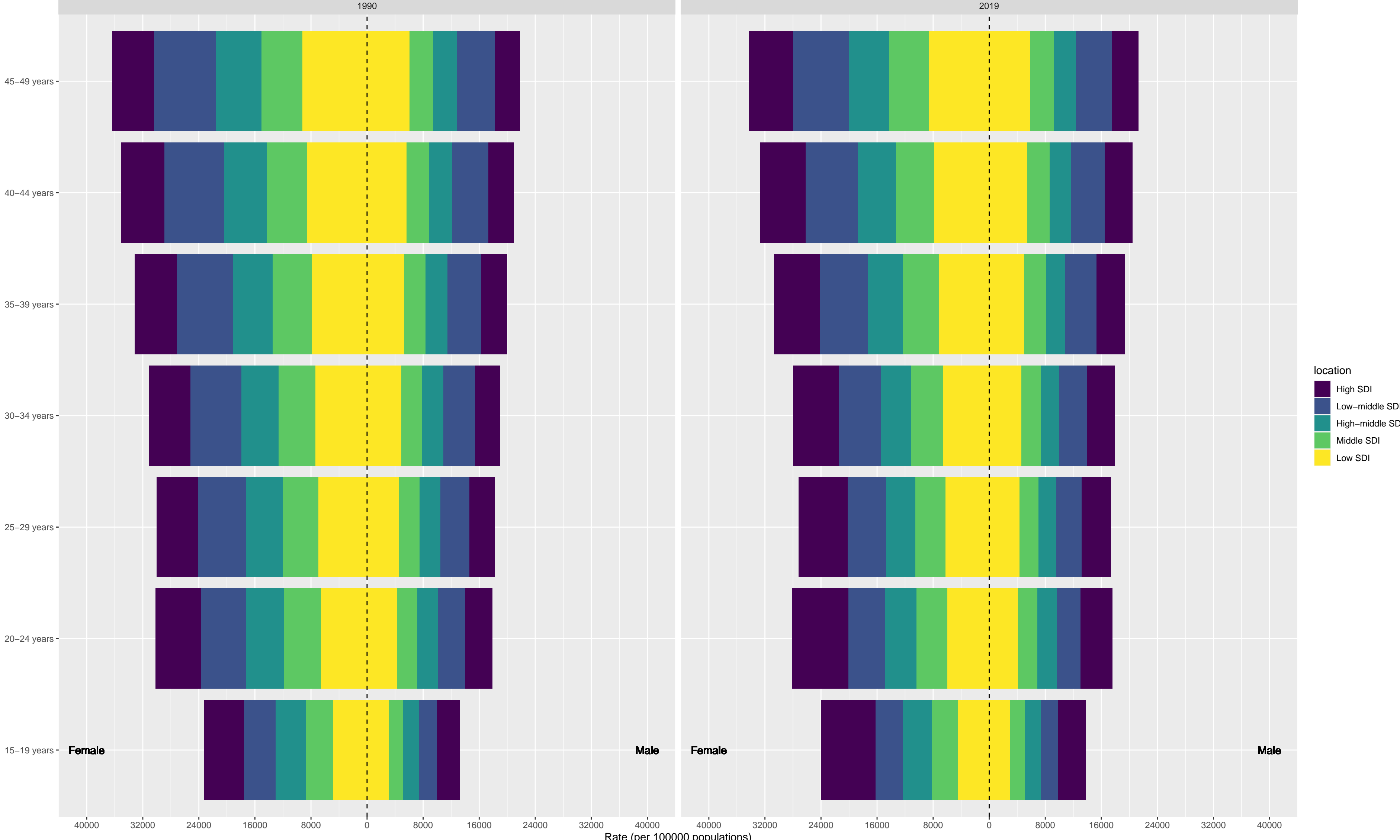

Supplement: Supplementary 6 — Figure 5: incidence of depression in five SDI regions by age group. [file 4747449.f6.pdf]

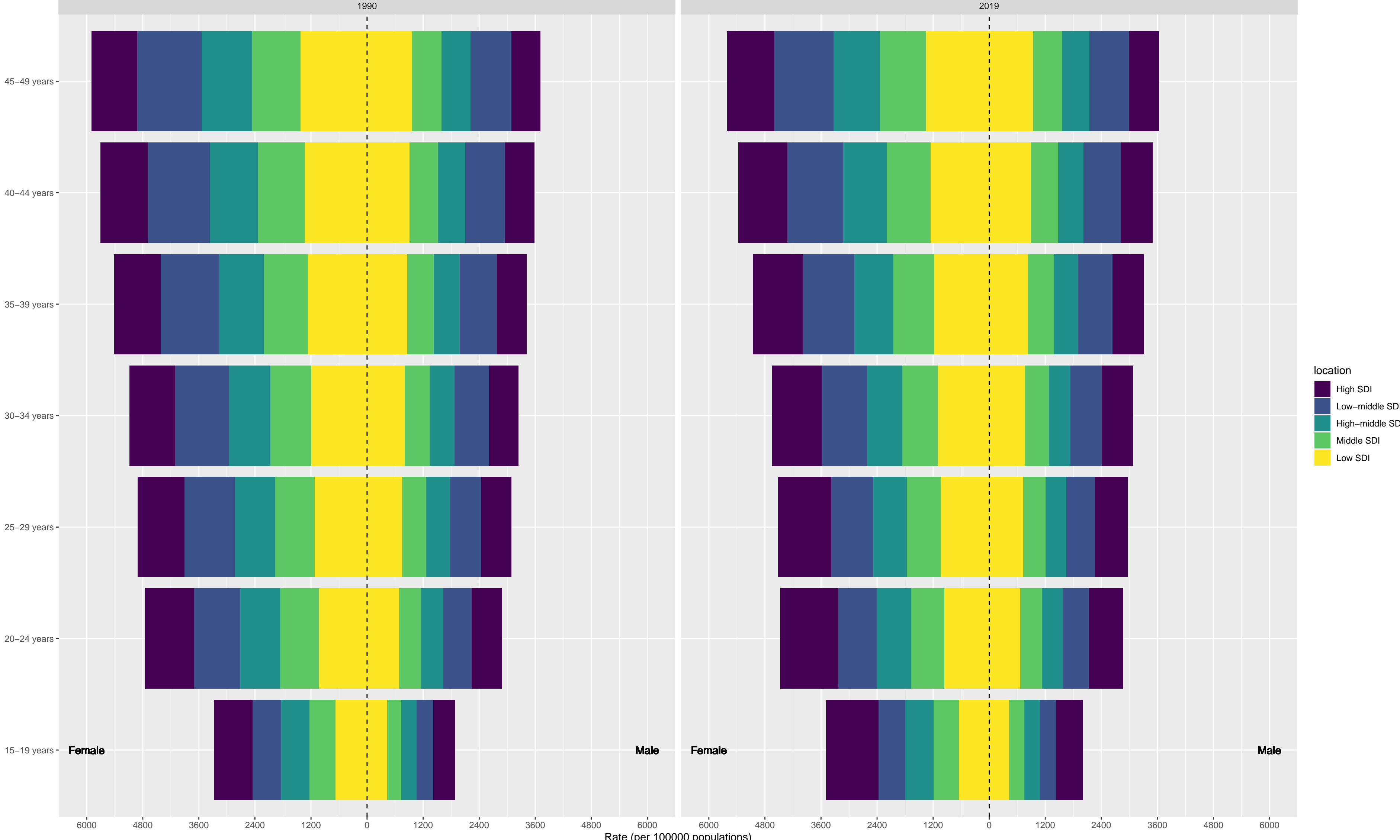

Supplement: Supplementary 7 — Figure 6: DALYs of depression in five SDI regions by age group. [file 4747449.f7.pdf]

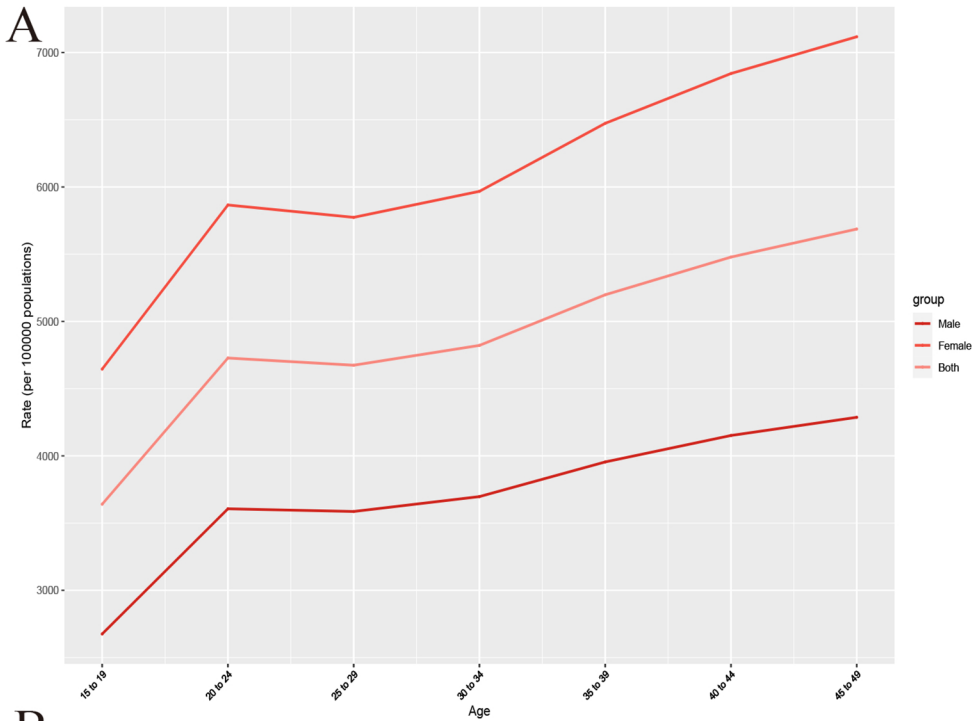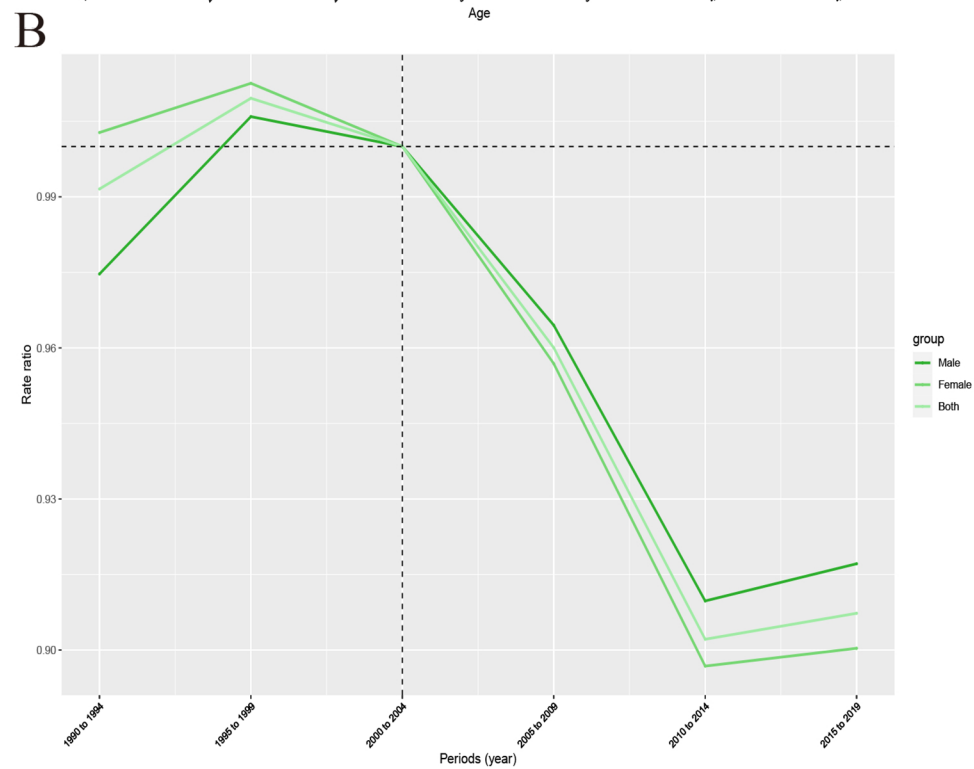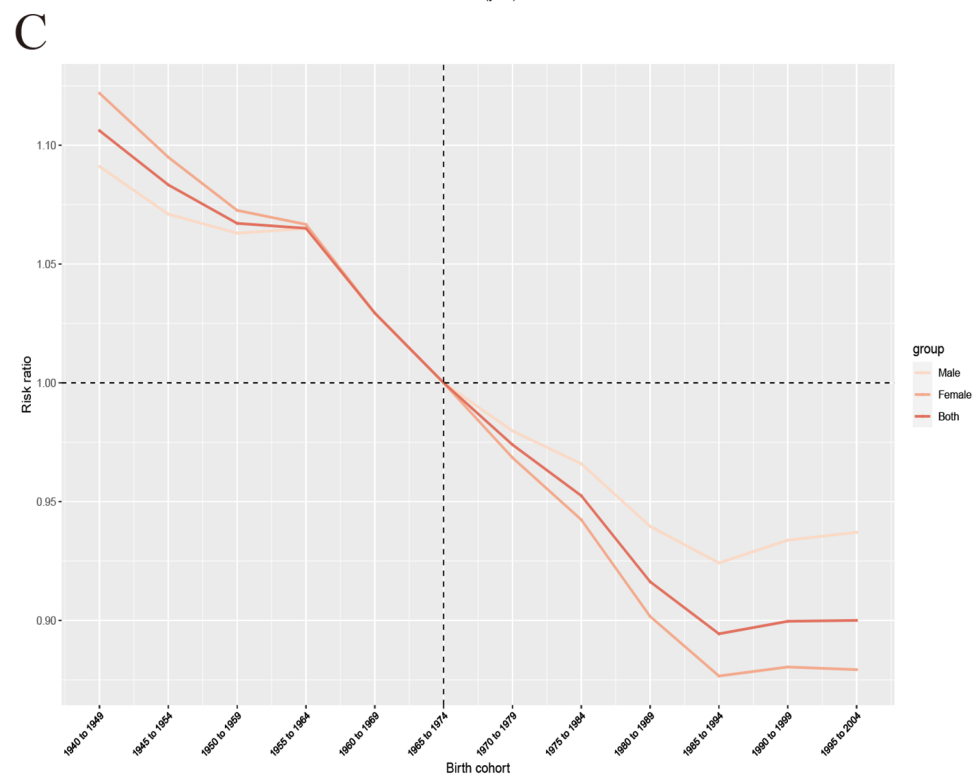

Supplement: Supplementary 8 — Figure 7: age, period, and cohort effects on incidence rates of depression by sex from 1990 and 2019 [file 4747449.f8.pdf]

A

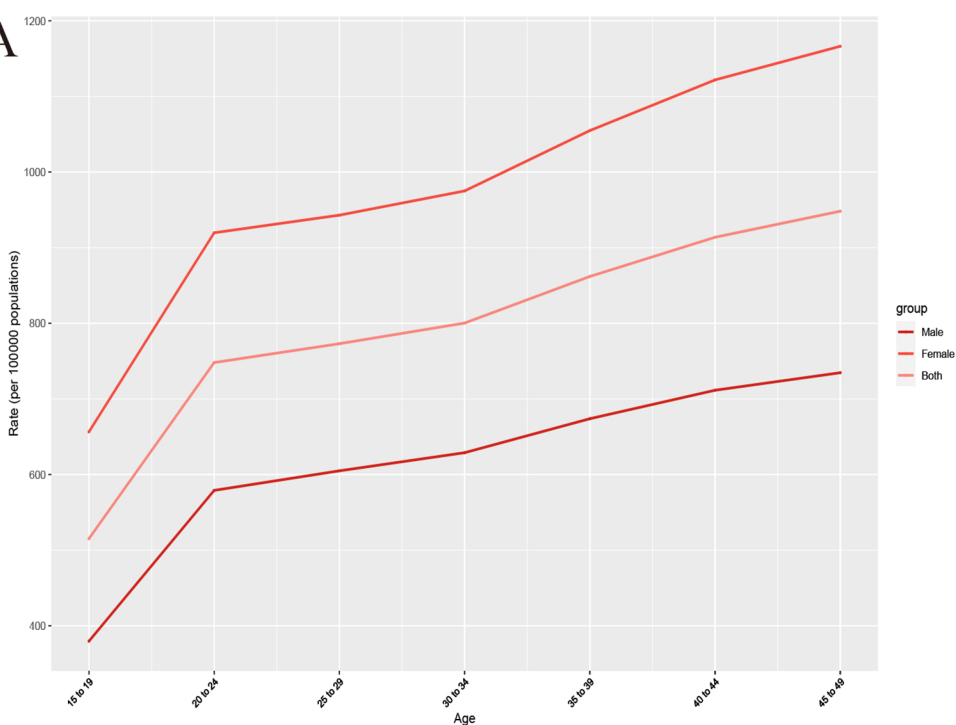

B

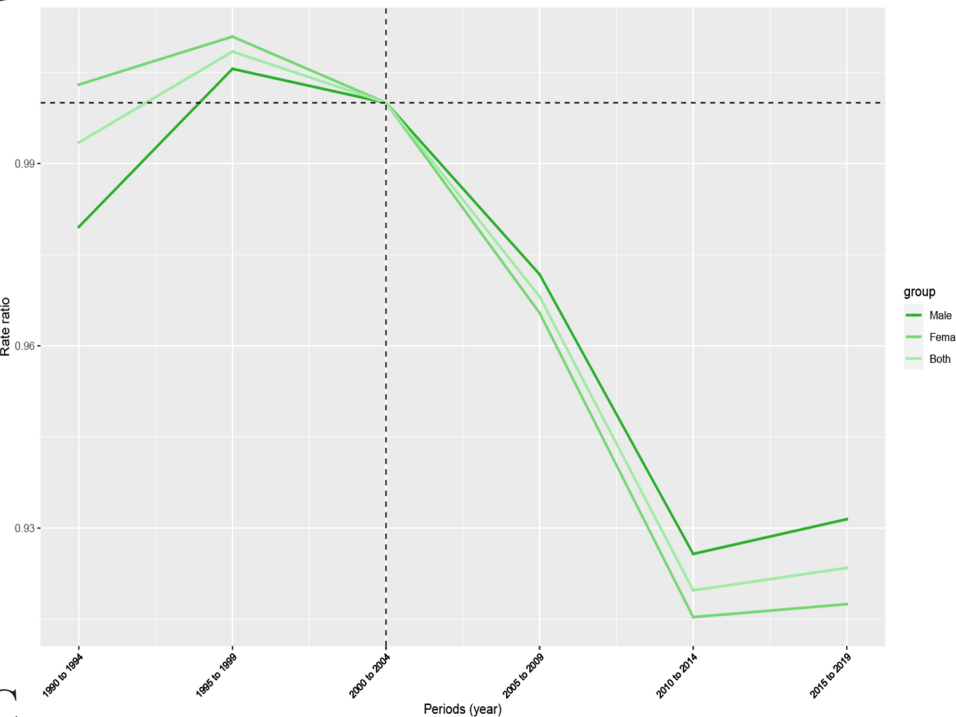

C

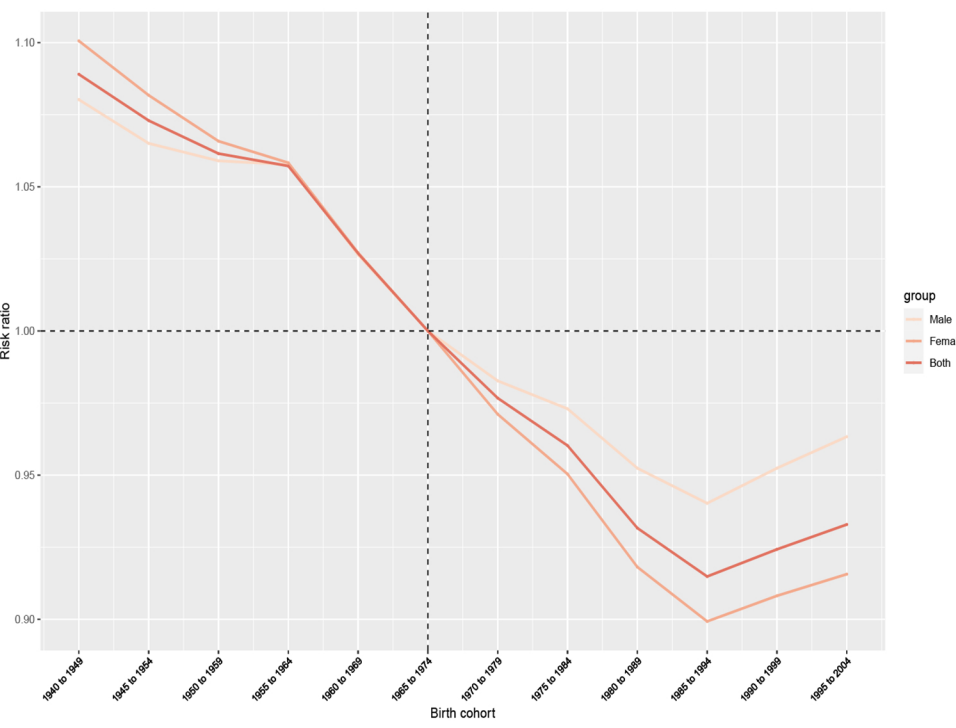

Supplement: Supplementary 9 — Figure 8: age, period, and cohort effects on DALYs' rates of depression by sex from 1990 and 2019 [file 4747449.f9.pdf]

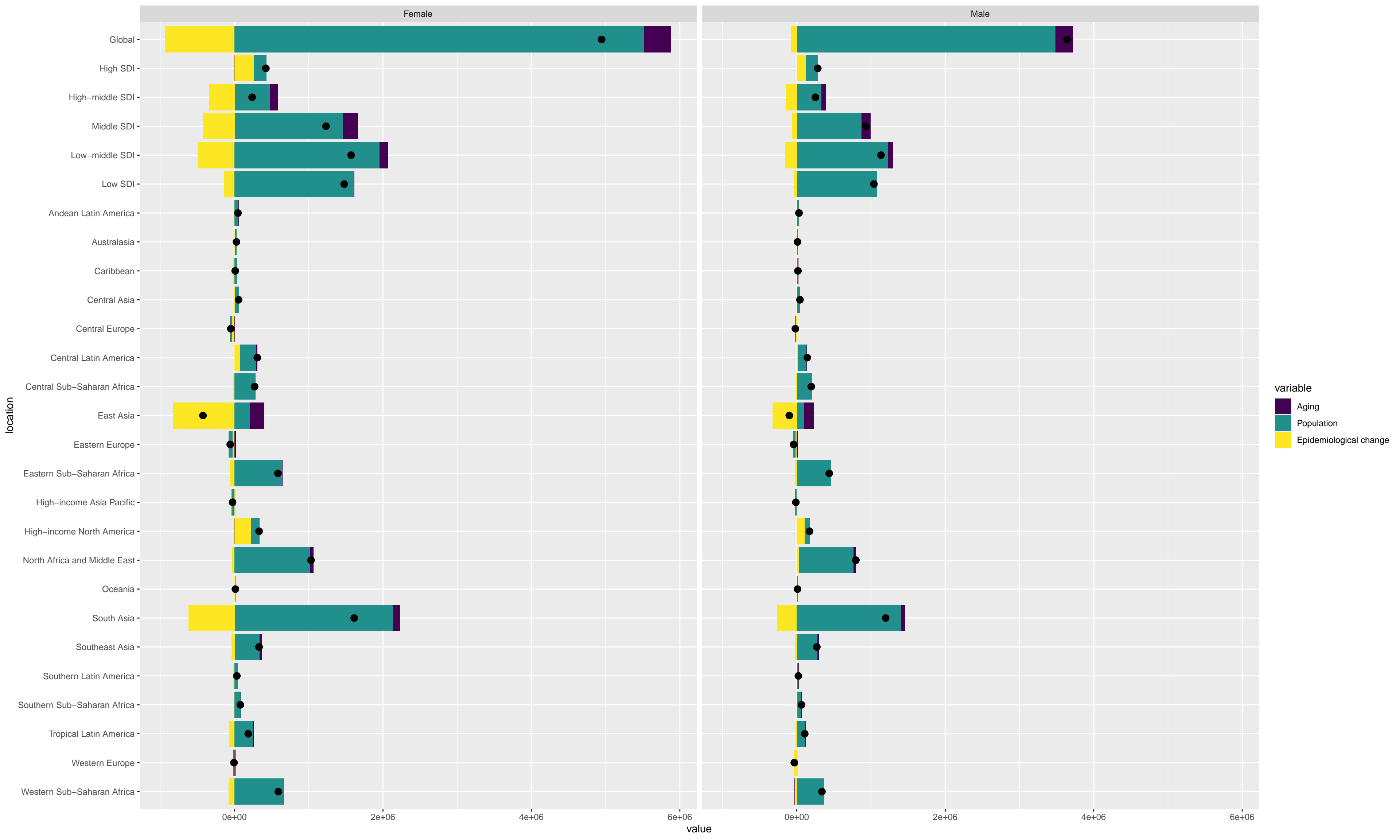

Supplement: Supplementary 10 — Figure 9: decomposition analysis of depression aged 10–24 years for change in DALYs. [file 4747449.f10.pdf]
