# Supplementary material for: Global, Regional, and National Epidemiology of Depression in Working-Age Individuals, 1990–2019
Source: Depress Anxiety. 2024 Aug 24;2024:4747449. doi: 10.1155/2024/4747449 (PMC11919199; doi:10.1155/2024/4747449)
Supplement: Supplementary 2 — Table 1: incidence of depression in working-age individuals between 1990 and 2019. Table 2: DALYs of depression in working-age individuals between 1990 and 2019. Table 3: prevalence of depression in working-age individuals between 1990 and 2019 in 204 countries. Table 4: incidence of depression in working-age individuals between 1990 and 2019 in 204 countries. Table 5: DALYs of depression in working-age individuals between 1990 and 2019 in 204 countries. Table 6: annual percentage change for depression prevalence overall (net drift) by sex from 1990 to 2019. [file 4747449.f2.docx]

| **TableS1. Incidence of depression in working-age individuals between 1990 and 2019** | | | | | |
| --- | --- | --- | --- | --- | --- |
| **Location** | **1990** | | **2019** | |  |
|  | **Number** | **ASIR** | **Number** | **ASIR** | **EAPC** |
| Global | 123939339.5 (104436519.8-146787731.8) | 4569.7 (3850.6-5412.1) | 173927508.9 (145180078.1-204945736.6) | 4419.9 (3689.4-5208.1) | -0.4 (-0.52--0.28) |
| High SDI | 20679662.5 (17859583.7-23978345.7) | 4808.4 (4152.7-5575.4) | 25560935.5 (21792110.1-30054662.9) | 5439.4 (4637.4-6395.7) | 0.35 (0.19-0.51) |
| High-middle SDI | 25312950.1 (21328950.2-29667180.8) | 4182.1 (3523.9-4901.5) | 27310347.9 (22938233.7-31928803.2) | 3748.7 (3148.6-4382.7) | -0.64 (-0.73--0.54) |
| Middle SDI | 35671196.8 (29781944.7-42335092.2) | 3953 (3300.3-4691.4) | 47397129.6 (39591118.5-55870377.1) | 3759.7 (3140.5-4431.8) | -0.41 (-0.51--0.31) |
| Low-middle SDI | 28996609.1 (23942344.9-34662180.6) | 5365 (4429.8-6413.2) | 44912548 (37259666.6-53300551.6) | 4811.8 (3991.9-5710.5) | -0.87 (-1.09--0.65) |
| Low SDI | 13206661.8 (10797847-16020752.7) | 5679.2 (4643.4-6889.4) | 28639066.4 (23331183.2-34549587.9) | 5300 (4317.7-6393.9) | -0.49 (-0.62--0.36) |
| Andean Latin America | 675489.7 (551882-832002.4) | 3627.2 (2963.5-4467.7) | 1122611.6 (910113.4-1360495.1) | 3388.8 (2747.3-4106.9) | -0.33 (-0.39--0.28) |
| Australasia | 763516.3 (649561.1-888923.9) | 7075.2 (6019.2-8237.3) | 995904.8 (828491.6-1197895.1) | 7364.2 (6126.2-8857.8) | 0.15 (0-0.3) |
| Caribbean | 1090212.9 (888316-1317607.7) | 5977.1 (4870.2-7223.8) | 1233823.9 (994786.9-1499667.2) | 5159.9 (4160.2-6271.7) | -0.6 (-0.67--0.54) |
| Central Asia | 1211809.7 (994921.1-1484088.6) | 3632.1 (2982-4448.2) | 1769402.7 (1453676.9-2145935.1) | 3623.7 (2977.1-4394.8) | 0.03 (-0.01-0.06) |
| Central Europe | 1974896 (1651584.4-2329169.7) | 3237.3 (2707.3-3818) | 1526563.3 (1261360.4-1825563.2) | 2895.8 (2392.8-3463) | -0.65 (-0.73--0.56) |
| Central Latin America | 3143148.6 (2593264.7-3766160.9) | 3854.3 (3180-4618.3) | 6019361.1 (5007315.5-7147405.1) | 4570.2 (3801.8-5426.7) | 0.69 (0.66-0.72) |
| Central Sub-Saharan Africa | 2123346.8 (1685194.5-2662700.3) | 8698.2 (6903.4-10907.7) | 5139773.8 (4062921.4-6404575.3) | 8267.5 (6535.4-10302) | -0.23 (-0.25--0.2) |
| East Asia | 23741502.3 (19954439.4-28010613.1) | 3438.9 (2890.3-4057.2) | 18566556.7 (15734606.8-21650394.3) | 2487.7 (2108.3-2900.9) | -1.53 (-1.72--1.34) |
| Eastern Europe | 5010900.1 (4120190.7-6036472.7) | 4542.1 (3734.7-5471.8) | 4310156.5 (3542968.6-5206526.9) | 4395.3 (3613-5309.4) | -0.32 (-0.42--0.22) |
| Eastern Sub-Saharan Africa | 5060293.8 (4126373.5-6125884.4) | 6097.9 (4972.5-7382) | 11254977 (9163121.1-13569125.7) | 5658.3 (4606.6-6821.7) | -0.43 (-0.49--0.36) |
| High-income Asia Pacific | 2707694.1 (2325554.7-3137589.4) | 2915 (2503.6-3377.8) | 2490490.7 (2134314.2-2886871.6) | 3069.2 (2630.3-3557.7) | 0.38 (0.26-0.49) |
| High-income North America | 7961429 (6836571.8-9325989.2) | 5356.2 (4599.5-6274.3) | 11683568.8 (10012429.1-13636129.3) | 7006.4 (6004.3-8177.3) | 0.62 (0.29-0.96) |
| North Africa and Middle East | 10672648.1 (8669550.4-12956934.8) | 6571.4 (5338-7977.9) | 22258305.9 (17916691.2-27089634.1) | 6670.9 (5369.7-8118.9) | 0.1 (0.06-0.15) |
| Oceania | 124670.3 (100041.2-155011.9) | 3937.6 (3159.7-4895.9) | 254930.4 (205346.5-315957.4) | 3747.6 (3018.7-4644.8) | -0.21 (-0.23--0.2) |
| South Asia | 28674002.1 (23847952-34244030.7) | 5417.2 (4505.4-6469.5) | 45222545.2 (37706551.5-53288150.9) | 4642.3 (3870.7-5470.2) | -1.23 (-1.54--0.91) |
| Southeast Asia | 6531117.8 (5373004.4-7808702.9) | 2762.8 (2272.9-3303.3) | 9412618.5 (7830134.9-11167005.7) | 2599.7 (2162.6-3084.3) | -0.27 (-0.34--0.19) |
| Southern Latin America | 1166246 (994783.2-1365299.6) | 4761.3 (4061.3-5574) | 1507142.7 (1282479.8-1763645.5) | 4428 (3767.9-5181.6) | -0.3 (-0.37--0.23) |
| Southern Sub-Saharan Africa | 1337076.1 (1119219.5-1590195.8) | 5125.2 (4290.1-6095.5) | 2218424.1 (1847969.3-2633040.9) | 5243.9 (4368.3-6224) | 0.24 (0.1-0.38) |
| Tropical Latin America | 4728831.2 (3964452.5-5626059.3) | 6021.2 (5047.9-7163.7) | 6488738.7 (5576635.5-7496441.4) | 5443.3 (4678.1-6288.6) | -0.55 (-0.94--0.16) |
| Western Europe | 10956431.9 (9490808.1-12534838.9) | 5664.5 (4906.8-6480.5) | 10672509.9 (9046205.2-12539619.2) | 5596.3 (4743.5-6575.4) | -0.03 (-0.05-0) |
| Western Sub-Saharan Africa | 4284076.7 (3486797.9-5193210) | 5030.3 (4094.1-6097.8) | 9779102.6 (7981817.8-11844458.4) | 4548.5 (3712.5-5509.1) | -0.41 (-0.66--0.16) |

| **TableS2. DALYs of depression in working-age individuals between 1990 and 2019** | | | | | |
| --- | --- | --- | --- | --- | --- |
| **Location** | **1990** | | **2019** | |  |
|  | **Number** | **ASDR** | **Number** | **ASDR** | **EAPC** |
| Global | 20093872.9 (13739547.3-27905974.8) | 740.9 (506.6-1028.9) | 28679797.1 (19599904.5-39765816.9) | 728.8 (498.1-1010.5) | -0.3 (-0.4--0.21) |
| High SDI | 3409914.2 (2334360.2-4640395.6) | 792.9 (542.8-1079) | 4115712.6 (2827065.2-5681872.6) | 875.8 (601.6-1209.1) | 0.26 (0.14-0.38) |
| High-middle SDI | 4172657.5 (2856986.8-5791236.8) | 689.4 (472-956.8) | 4661684.2 (3199364.5-6451725.4) | 639.9 (439.2-885.6) | -0.48 (-0.56--0.4) |
| Middle SDI | 5893676.3 (3992874-8198313) | 653.1 (442.5-908.5) | 8056096.5 (5498323.1-11097779.9) | 639 (436.1-880.3) | -0.28 (-0.36--0.2) |
| Low-middle SDI | 4526176.2 (3057137.5-6356879.5) | 837.4 (565.6-1176.2) | 7231797.8 (4928637.4-10047058.9) | 774.8 (528-1076.4) | -0.7 (-0.88--0.51) |
| Low SDI | 2079908.5 (1384231.7-2940564) | 894.4 (595.3-1264.5) | 4597018.2 (3058339.7-6497903) | 850.7 (566-1202.5) | -0.39 (-0.49--0.28) |
| Andean Latin America | 109923.6 (72626-156851.5) | 590.3 (390-842.3) | 186860 (124956-263139.1) | 564.1 (377.2-794.3) | -0.24 (-0.28--0.19) |
| Australasia | 119235.6 (80864.9-163592.4) | 1104.9 (749.3-1515.9) | 155632.8 (105645-219249.3) | 1150.8 (781.2-1621.2) | 0.16 (0.02-0.29) |
| Caribbean | 168312.8 (112400.1-240725) | 922.8 (616.2-1319.8) | 193526.3 (127881.9-275872.3) | 809.3 (534.8-1153.7) | -0.55 (-0.61--0.49) |
| Central Asia | 204770.6 (135464.3-284999.7) | 613.7 (406-854.2) | 303476.2 (203956.3-423846) | 621.5 (417.7-868) | 0.07 (0.04-0.1) |
| Central Europe | 344276.4 (234625.3-476315.7) | 564.3 (384.6-780.8) | 276337.1 (187628.7-386450.9) | 524.2 (355.9-733.1) | -0.45 (-0.52--0.38) |
| Central Latin America | 500416.4 (336255.5-700098.4) | 613.6 (412.3-858.5) | 948979.6 (644116.8-1319513) | 720.5 (489-1001.8) | 0.63 (0.61-0.66) |
| Central Sub-Saharan Africa | 320276.3 (209148.1-459817.9) | 1312 (856.8-1883.6) | 786052.2 (512093.8-1133767.1) | 1264.4 (823.7-1823.7) | -0.17 (-0.2--0.15) |
| East Asia | 4061436.3 (2777711.5-5684933.7) | 588.3 (402.3-823.4) | 3536184.2 (2438302.3-4902192.7) | 473.8 (326.7-656.8) | -1.09 (-1.24--0.94) |
| Eastern Europe | 816340.5 (556532.5-1144264.1) | 740 (504.5-1037.2) | 716847.4 (486239.6-1008603) | 731 (495.8-1028.5) | -0.21 (-0.29--0.12) |
| Eastern Sub-Saharan Africa | 808760.5 (538424.2-1138572.5) | 974.6 (648.8-1372) | 1831370.8 (1219555-2588308.2) | 920.7 (613.1-1301.2) | -0.33 (-0.39--0.28) |
| High-income Asia Pacific | 442826.2 (303931.4-609316.2) | 476.7 (327.2-656) | 404524.2 (276901.3-553452.3) | 498.5 (341.2-682.1) | 0.3 (0.2-0.4) |
| High-income North America | 1356193.9 (935067-1847336.2) | 912.4 (629.1-1242.8) | 1859492.7 (1279268.4-2556123.1) | 1115.1 (767.2-1532.9) | 0.42 (0.17-0.67) |
| North Africa and Middle East | 1656593.6 (1106340.9-2320419.5) | 1020 (681.2-1428.7) | 3483388.5 (2322867.4-4913723) | 1044 (696.2-1472.7) | 0.13 (0.09-0.17) |
| Oceania | 20810.7 (13723.9-29386.2) | 657.3 (433.5-928.1) | 43394.1 (28993.5-62518.3) | 637.9 (426.2-919.1) | -0.14 (-0.15--0.12) |
| South Asia | 4427682.9 (3017503.4-6103807.1) | 836.5 (570.1-1153.2) | 7236926.3 (4940003-10090877.8) | 742.9 (507.1-1035.9) | -1.02 (-1.29--0.75) |
| Southeast Asia | 1172843.3 (789105.3-1631110.9) | 496.1 (333.8-690) | 1773975.2 (1201443.6-2468595.5) | 490 (331.8-681.8) | -0.07 (-0.13--0.02) |
| Southern Latin America | 181191.4 (122513.5-250101.8) | 739.7 (500.2-1021.1) | 235092.3 (158649.1-323484.4) | 690.7 (466.1-950.4) | -0.29 (-0.36--0.22) |
| Southern Sub-Saharan Africa | 216099 (147117.4-300433.3) | 828.3 (563.9-1151.6) | 359698.9 (247006.7-496498.3) | 850.3 (583.9-1173.6) | 0.23 (0.09-0.36) |
| Tropical Latin America | 718959.1 (485818.6-992543.5) | 915.5 (618.6-1263.8) | 1011900.9 (694872-1390111.3) | 848.9 (582.9-1166.1) | -0.44 (-0.78--0.09) |
| Western Europe | 1750417 (1198268.5-2396058.7) | 905 (619.5-1238.8) | 1710497.7 (1166357.5-2375170.1) | 896.9 (611.6-1245.5) | -0.02 (-0.04-0) |
| Western Sub-Saharan Africa | 696506.9 (464880.5-978962) | 817.8 (545.9-1149.5) | 1625639.6 (1091736.9-2279679.2) | 756.1 (507.8-1060.3) | -0.31 (-0.52--0.1) |

| **TableS3. Prevalence of depression in working-age individuals between 1990 and 2019 in 204 countries** | | | | | | | | | |
| --- | --- | --- | --- | --- | --- | --- | --- | --- | --- |
| **Location** | **1990** | | | | **2019** | | | |  |
|  | **Number** | | | **ASPR** | **Number** | | **ASPR** | | **EAPC** |
| Mexico | | 1482405.1 (1288993.2-1706016.2) | 3487.1 (3032.1-4013.1) | | 2996250.7 (2608849.4-3455573.6) | 4520.1 (3935.7-5213.1) | | 1.14 (1.02-1.26) | |
| Haiti | | 136096.9 (112848.8-164663) | 4645 (3851.5-5619.9) | | 296187 (245712.4-358674.3) | 4520 (3749.7-5473.6) | | -0.11 (-0.13--0.08) | |
| Viet Nam | | 971655.7 (805950.2-1179210.5) | 2961.6 (2456.5-3594.2) | | 1598256.8 (1322290.1-1992509.2) | 3014.3 (2493.8-3757.9) | | 0.02 (-0.01-0.05) | |
| Bhutan | | 13275.8 (11025.4-15968.3) | 4379.6 (3637.2-5267.8) | | 19547.8 (16320.2-23569.3) | 4487.3 (3746.3-5410.4) | | -0.07 (-0.15-0.01) | |
| Jamaica | | 41868.1 (34313.7-51163.2) | 3597 (2948-4395.6) | | 57899.7 (48035.1-69971.6) | 3781.7 (3137.4-4570.1) | | 0.11 (0.05-0.16) | |
| Nicaragua | | 67772.4 (56281.2-82076.3) | 3931.2 (3264.7-4761) | | 140855.3 (118238.5-169763.8) | 4019.5 (3374.1-4844.4) | | 0.01 (-0.06-0.08) | |
| Kyrgyzstan | | 82343.3 (68883.6-100094.1) | 3929.8 (3287.4-4776.9) | | 130913.5 (109668.2-157053.6) | 3897.5 (3265-4675.8) | | -0.07 (-0.09--0.05) | |
| Georgia | | 96861.9 (80387.3-117760.1) | 3611.4 (2997.1-4390.5) | | 63133.7 (52502-77259.6) | 3816.9 (3174.1-4670.9) | | 0.18 (0.16-0.21) | |
| Lebanon | | 84826.5 (71114.9-101822.7) | 5629 (4719.1-6756.9) | | 163631 (134501.1-197170.9) | 6123.6 (5033.5-7378.8) | | 0.29 (0.17-0.42) | |
| Kazakhstan | | 324054.8 (273726-390490.2) | 3941.1 (3329-4749) | | 389266.7 (329936.2-471725.2) | 4193.5 (3554.3-5081.8) | | 0.32 (0.25-0.38) | |
| Namibia | | 27643.1 (23013.4-33639.9) | 4200 (3496.5-5111.1) | | 53735.4 (44559.4-65065.8) | 4281 (3549.9-5183.6) | | -0.06 (-0.15-0.04) | |
| Republic of Korea | | 670620.1 (571973.4-793428.3) | 2591.2 (2210-3065.7) | | 710348.6 (613694-837144.5) | 2727.2 (2356.2-3214.1) | | 0.21 (0.1-0.32) | |
| Timor-Leste | | 14320 (11896.1-17109.3) | 3742.4 (3108.9-4471.4) | | 21050.2 (17541.8-25304.9) | 3225.1 (2687.6-3877) | | -0.65 (-0.72--0.57) | |
| China | | 23572651.5 (20586088.3-27176403.5) | 3526.4 (3079.6-4065.5) | | 22319553.4 (19543443.3-25476524.2) | 3096.8 (2711.6-3534.8) | | -0.73 (-0.85--0.62) | |
| Eritrea | | 74603.8 (61808.6-89778) | 5472.8 (4534.2-6586) | | 184264.3 (152848.2-222634.4) | 5334.1 (4424.7-6444.8) | | -0.1 (-0.13--0.07) | |
| Iceland | | 5590 (4737.1-6650.3) | 4220.6 (3576.6-5021.2) | | 6249.1 (5293.2-7550.8) | 3828.7 (3243.1-4626.2) | | -0.45 (-0.51--0.39) | |
| Panama | | 42720.6 (35716.1-51637.7) | 3470.5 (2901.5-4194.9) | | 74890.6 (62973.9-89877.9) | 3563.3 (2996.3-4276.4) | | 0.1 (0.07-0.13) | |
| Serbia | | 172186.8 (145929.8-208078.4) | 3748.6 (3176.9-4529.9) | | 138050.2 (115961.9-168008.9) | 3449 (2897.2-4197.5) | | -0.37 (-0.41--0.32) | |
| India | | 19517697.7 (16940914.2-22541788.8) | 4646.4 (4033-5366.3) | | 31424840.5 (27559116.4-36289659.5) | 4137.1 (3628.2-4777.5) | | -1.05 (-1.33--0.76) | |
| Libya | | 103334 (85643.8-124329.9) | 5340.1 (4425.9-6425.2) | | 241088.6 (202071-288885.4) | 5868.7 (4918.9-7032.2) | | 0.33 (0.32-0.35) | |
| South Africa | | 969709.9 (843016.1-1122610.6) | 5092.6 (4427.2-5895.5) | | 1613404.9 (1412854.3-1853027.3) | 5298.2 (4639.7-6085.1) | | 0.25 (0.12-0.38) | |
| Democratic People's Republic of Korea | | 339081.2 (282495.3-409650.5) | 3177.9 (2647.6-3839.3) | | 425645.7 (350457.2-526588.2) | 3058.8 (2518.5-3784.2) | | -0.17 (-0.19--0.15) | |
| Uruguay | | 50159.5 (42308-59641.9) | 3375.9 (2847.5-4014.2) | | 62865.4 (53100.4-74477.2) | 3814.2 (3221.8-4518.7) | | 0.65 (0.57-0.73) | |
| Japan | | 1796030.7 (1588386.1-2030167.8) | 2764.8 (2445.2-3125.2) | | 1487760.1 (1313491.8-1681684.8) | 2874.6 (2537.9-3249.3) | | 0.28 (0.1-0.45) | |
| Poland | | 565292.3 (489631.1-657994.4) | 2978.7 (2580-3467.2) | | 558854.3 (483921.3-651092.2) | 3070.6 (2658.9-3577.4) | | -0.21 (-0.32--0.1) | |
| Saint Vincent and the Grenadines | | 2055.4 (1697.7-2457.9) | 3853.3 (3182.6-4607.9) | | 2431.6 (2037.6-2873.1) | 4252.2 (3563.2-5024.4) | | 0.37 (0.34-0.41) | |
| Australia | | 555289.9 (483992.5-642693.8) | 6179.1 (5385.7-7151.7) | | 736483.1 (622621.7-875598.8) | 6382.8 (5396-7588.5) | | 0.13 (-0.02-0.27) | |
| Cook Islands | | 363.6 (288.5-450.5) | 3840.9 (3047.6-4758.7) | | 331 (267.4-407.3) | 4054.1 (3275.2-4988.1) | | 0.19 (0.17-0.21) | |
| Liberia | | 43487.8 (36280.8-52506.2) | 5321.5 (4439.6-6425.1) | | 127613.5 (106280.4-152123.3) | 5159.4 (4296.9-6150.3) | | 0.41 (0.25-0.57) | |
| Greenland | | 3407.4 (2897.5-4037.8) | 10187.3 (8662.8-12072) | | 2409.1 (2044.5-2847.4) | 9062.3 (7690.9-10710.8) | | -0.42 (-0.46--0.39) | |
| Tajikistan | | 78713 (65347.8-96144) | 3263.9 (2709.7-3986.7) | | 159622.1 (132923.5-195717) | 3212.4 (2675.1-3938.8) | | -0.13 (-0.19--0.08) | |
| Fiji | | 14008 (11755.1-16932.7) | 3543.2 (2973.4-4283) | | 16999.5 (14170.1-20575.3) | 3646.7 (3039.7-4413.7) | | 0.06 (0.05-0.08) | |
| Bermuda | | 1539.1 (1295.4-1829.6) | 4547.5 (3827.6-5405.9) | | 1122.7 (932.9-1340.7) | 4047.5 (3363.3-4833.4) | | -0.49 (-0.53--0.45) | |
| Israel | | 129828.3 (109008.8-153785.2) | 5347.2 (4489.7-6334) | | 225331.4 (190896.8-267682.2) | 5148.7 (4361.9-6116.4) | | -0.41 (-0.58--0.23) | |
| United States Virgin Islands | | 2235.4 (1875.2-2649.7) | 4073.4 (3417.2-4828.5) | | 1841.4 (1535.3-2213.7) | 4239.7 (3534.8-5096.8) | | 0.16 (0.14-0.18) | |
| Pakistan | | 2174140.7 (1843660-2557629.3) | 4382.8 (3716.6-5155.9) | | 4879738.3 (4152187.3-5769159.8) | 4365.7 (3714.8-5161.5) | | -0.06 (-0.1--0.01) | |
| Guam | | 3140 (2641.9-3747.6) | 4062.8 (3418.4-4849.1) | | 3369.6 (2831.8-4025.3) | 4154.3 (3491.3-4962.7) | | 0.08 (0.06-0.1) | |
| Mauritania | | 38532.6 (31993.8-46621.8) | 4224.9 (3507.9-5111.8) | | 78805.5 (65066.6-96833) | 4068 (3358.8-4998.6) | | -0.19 (-0.24--0.14) | |
| Cambodia | | 179630.7 (148628.3-214173) | 3910.5 (3235.6-4662.5) | | 310582.8 (260848.6-373968.5) | 3534.5 (2968.6-4255.9) | | -0.49 (-0.54--0.43) | |
| Singapore | | 70193.3 (61796.1-81099.1) | 3691.5 (3249.9-4265) | | 80593.2 (69411.8-95512.8) | 2620.2 (2256.7-3105.3) | | -1.54 (-1.73--1.34) | |
| Nepal | | 450399.6 (379472.6-539300.6) | 5062.9 (4265.7-6062.3) | | 856891.9 (726638.7-1015135) | 5295.1 (4490.2-6272.9) | | 0.26 (0.16-0.37) | |
| South Sudan | | 140087.2 (115732.7-169105.3) | 5122.5 (4231.9-6183.5) | | 222891 (185210.3-267084.8) | 5286.3 (4392.6-6334.4) | | 0.12 (0.11-0.13) | |
| Slovakia | | 94213.3 (79406.5-114286.8) | 3525.8 (2971.7-4277) | | 89363.5 (73849.5-109752.1) | 3396.3 (2806.7-4171.2) | | -0.23 (-0.35--0.11) | |
| Mongolia | | 47862.4 (39944.1-57969.3) | 4664.9 (3893.1-5649.9) | | 88224.7 (73671.9-105434.4) | 4880.6 (4075.5-5832.6) | | 0.14 (0.12-0.16) | |
| Italy | | 1345953.8 (1152173.1-1562517.7) | 4686 (4011.3-5440) | | 1181640.3 (1020092.3-1371646.2) | 4659.6 (4022.5-5408.8) | | -0.43 (-0.57--0.28) | |
| Kiribati | | 1427.4 (1178.2-1728.1) | 3892.5 (3213.1-4712.6) | | 2211.9 (1833-2666.4) | 3660.5 (3033.5-4412.6) | | -0.25 (-0.29--0.2) | |
| Ireland | | 94219.2 (80925.4-109704.9) | 5288.7 (4542.5-6157.9) | | 131690.9 (112859.9-154309.9) | 5647.9 (4840.3-6618) | | 0.1 (0.03-0.17) | |
| Andorra | | 1546.2 (1305.5-1848.2) | 4770.2 (4027.6-5701.8) | | 1978.4 (1652-2373.2) | 4830.3 (4033.5-5794.3) | | 0.01 (0-0.03) | |
| Kenya | | 537685.2 (466246.7-620605.7) | 5297.5 (4593.7-6114.5) | | 1353078.7 (1173741.2-1558709.9) | 5194.6 (4506.1-5984) | | -0.13 (-0.19--0.06) | |
| Mali | | 150371.7 (125897.6-182283.3) | 4161.1 (3483.9-5044.2) | | 381017.8 (313760.7-463373.1) | 3945.8 (3249.3-4798.7) | | -0.24 (-0.32--0.17) | |
| Morocco | | 804217 (672300.7-965234.1) | 6459.8 (5400.2-7753.2) | | 1251447.9 (1049868.4-1492280.5) | 6528.2 (5476.7-7784.6) | | 0.02 (0-0.03) | |
| Romania | | 371747.4 (310785.5-453308.6) | 3277.7 (2740.2-3996.8) | | 291740.2 (242190.3-359267) | 3369.4 (2797.1-4149.3) | | 0.11 (0.09-0.14) | |
| Zimbabwe | | 163105.3 (133949.4-197689) | 3542.6 (2909.4-4293.8) | | 280033.4 (228751.3-342245.9) | 3722.8 (3041.1-4549.9) | | 0.17 (0.1-0.24) | |
| Eswatini | | 16696.6 (13803.6-20214.9) | 4689.8 (3877.3-5678.1) | | 31259.5 (26129.9-37663.7) | 5161.2 (4314.3-6218.6) | | 0.34 (0.3-0.39) | |
| United States of America | | 7354304.1 (6498516.7-8395257.2) | 5494.2 (4854.9-6271.9) | | 9599583.6 (8503099.8-10899310.5) | 6376.1 (5647.8-7239.4) | | 0.26 (0.08-0.43) | |
| Turkmenistan | | 62928 (52662.5-76052.7) | 3574.5 (2991.4-4320) | | 92807.4 (76998-113658.3) | 3522 (2922-4313.3) | | -0.1 (-0.13--0.06) | |
| Venezuela (Bolivarian Republic of) | | 351718.8 (293094.6-424651.6) | 3665.3 (3054.3-4425.3) | | 568271.6 (475442.9-682061.5) | 3948.2 (3303.3-4738.8) | | 0.19 (0.16-0.22) | |
| Marshall Islands | | 719.7 (597.8-872.3) | 3575.1 (2969.8-4333.1) | | 1088.9 (909.1-1321.8) | 3596 (3002.3-4365.3) | | 0.01 (0-0.02) | |
| Trinidad and Tobago | | 31882.3 (26930.9-38454.8) | 5150.6 (4350.7-6212.4) | | 34711.5 (29195.6-41231) | 4997.3 (4203.2-5935.9) | | -0.14 (-0.19--0.1) | |
| Taiwan (Province of China) | | 303853.3 (250302.6-375001.5) | 2699.5 (2223.8-3331.7) | | 370653.6 (305062.6-458472.8) | 3171.9 (2610.6-3923.4) | | 0.57 (0.51-0.63) | |
| Angola | | 352012.1 (291714.1-424909) | 7527.5 (6238.1-9086.4) | | 967579 (796842.7-1165432.6) | 7151.8 (5889.8-8614.2) | | -0.22 (-0.25--0.19) | |
| Palestine | | 67949.5 (56125.2-82286.8) | 7694.4 (6355.5-9318) | | 197665.5 (163931.2-238232.6) | 7792.9 (6462.9-9392.2) | | 0.16 (-0.07-0.38) | |
| Suriname | | 11049.1 (9287.6-13261.8) | 5619.6 (4723.7-6745) | | 17408.8 (14548.9-20617.7) | 6040.3 (5048-7153.7) | | 0.33 (0.27-0.39) | |
| Saint Lucia | | 2609.9 (2172.1-3155.8) | 3922.3 (3264.4-4742.7) | | 3938.2 (3292.5-4715.2) | 4206.8 (3517.1-5036.8) | | 0.24 (0.21-0.27) | |
| Niger | | 157649 (130710.4-189397.9) | 4713.1 (3907.7-5662.3) | | 432754.4 (358962.6-518901.7) | 4567 (3788.2-5476.1) | | -0.11 (-0.13--0.09) | |
| Bahamas | | 5304.9 (4421.9-6408.7) | 3724.8 (3104.8-4499.8) | | 7842.1 (6544.1-9405.8) | 3830.6 (3196.6-4594.5) | | 0.04 (0-0.08) | |
| Ethiopia | | 1264083 (1080629.9-1477944.1) | 5714 (4884.8-6680.8) | | 2659269.1 (2269439-3107762.7) | 5065 (4322.5-5919.3) | | -0.54 (-0.66--0.41) | |
| Micronesia (Federated States of) | | 1723.1 (1427.9-2071.1) | 3641.5 (3017.7-4376.9) | | 1895.7 (1594.6-2279.3) | 3539.4 (2977.1-4255.4) | | -0.15 (-0.16--0.13) | |
| Lao People's Democratic Republic | | 66345.3 (55237.9-79538.3) | 3569.3 (2971.7-4279) | | 131013.9 (109762.9-158352) | 3362.5 (2817.1-4064.1) | | -0.3 (-0.4--0.19) | |
| Belarus | | 235452.6 (197446.5-282257.4) | 4661 (3908.6-5587.5) | | 210676.9 (175639.6-253183.5) | 4835.2 (4031-5810.7) | | 0.08 (0-0.16) | |
| Malta | | 7811.2 (6584.1-9436.4) | 4078.7 (3438-4927.4) | | 8022.4 (6737.4-9660.9) | 4072.2 (3419.9-4903.9) | | -0.1 (-0.17--0.04) | |
| Samoa | | 2767.9 (2300.3-3365.2) | 3543.3 (2944.8-4307.9) | | 3511.8 (2951.5-4240.1) | 3377.5 (2838.6-4077.9) | | -0.22 (-0.25--0.2) | |
| Brazil | | 3803841.6 (3297860.4-4383701.4) | 4962.9 (4302.7-5719.4) | | 5400952.6 (4805175.6-6105940.1) | 4676.6 (4160.7-5287) | | -0.37 (-0.68--0.05) | |
| Dominica | | 1270.5 (1055.1-1522) | 3599.5 (2989.4-4312.1) | | 1303.4 (1082.3-1569.5) | 3837.5 (3186.6-4620.9) | | 0.22 (0.19-0.25) | |
| Latvia | | 66297.5 (55955.8-78494.1) | 5157.3 (4352.8-6106.1) | | 38494.1 (32823-45589.5) | 4699.1 (4006.8-5565.2) | | -0.44 (-0.51--0.37) | |
| Uzbekistan | | 357018.1 (299545-434075.1) | 3648.6 (3061.2-4436.1) | | 666430.2 (555766.6-814559.6) | 3686 (3073.9-4505.3) | | 0 (-0.02-0.02) | |
| Philippines | | 1116455.4 (954120.9-1303059.9) | 3582.9 (3061.9-4181.7) | | 1919966.1 (1652209.7-2215975.6) | 3284.3 (2826.2-3790.6) | | -0.34 (-0.48--0.2) | |
| Luxembourg | | 10098.9 (8550.1-12038.5) | 5076.3 (4297.8-6051.2) | | 12998.3 (11244.4-15361.4) | 4235.7 (3664.2-5005.8) | | -0.77 (-0.83--0.71) | |
| Mauritius | | 32078.2 (26870-38566.3) | 5274.9 (4418.5-6341.8) | | 31694.5 (26568.7-37567.1) | 4875.5 (4087-5778.9) | | -0.34 (-0.44--0.25) | |
| Paraguay | | 81915.3 (68131.5-98268) | 4334.4 (3605.1-5199.7) | | 173479.3 (145875.5-208503.2) | 4665.8 (3923.4-5607.8) | | 0.26 (0.17-0.35) | |
| Benin | | 98049.3 (81545.5-119070.7) | 4896.9 (4072.6-5946.7) | | 284905.2 (237044.1-344901.3) | 4892 (4070.2-5922.2) | | -0.01 (-0.02-0) | |
| Malaysia | | 335323.7 (282327.9-404175.2) | 3723.8 (3135.3-4488.4) | | 770013.4 (655897-906270.9) | 4411.1 (3757.3-5191.6) | | 0.77 (0.62-0.92) | |
| Ecuador | | 200005.9 (168936.3-240296.5) | 4039.9 (3412.3-4853.7) | | 371637.7 (311556.8-446318.7) | 4042.7 (3389.1-4855.1) | | -0.02 (-0.04-0.01) | |
| Monaco | | 744.4 (602.1-942.9) | 5405.4 (4372-6846.8) | | 764.7 (612.4-959.3) | 5365.5 (4296.6-6730.3) | | -0.01 (-0.02-0) | |
| Qatar | | 16577.4 (13804.5-20123.2) | 5677.4 (4727.8-6891.8) | | 115217.8 (95388.5-139508.5) | 5335.9 (4417.6-6460.8) | | -0.42 (-0.53--0.32) | |
| El Salvador | | 118010.9 (99673.1-142419.8) | 4723.6 (3989.6-5700.7) | | 141158.5 (118356.8-170495) | 4302.4 (3607.4-5196.6) | | -0.42 (-0.46--0.38) | |
| Armenia | | 54366.1 (44767.8-66548.6) | 3183.4 (2621.4-3896.7) | | 51882.3 (42991.6-63440.8) | 3511.1 (2909.4-4293.3) | | 0.38 (0.35-0.41) | |
| Iran (Islamic Republic of) | | 1636356.6 (1353093.3-1981343.7) | 6189.6 (5118.1-7494.5) | | 3208465.1 (2688780.2-3860077.4) | 6787 (5687.6-8165.3) | | 0.63 (0.47-0.79) | |
| Cuba | | 374528.1 (313205.2-445248.6) | 6098.9 (5100.3-7250.6) | | 244319 (205339.8-290138.5) | 4603.5 (3869-5466.8) | | -1.17 (-1.29--1.06) | |
| Nigeria | | 1947107.8 (1661450.4-2284667) | 4751.7 (4054.6-5575.4) | | 4155007.9 (3563059-4851144.6) | 4118.8 (3532-4808.9) | | -0.58 (-0.95--0.21) | |
| Myanmar | | 495930.3 (405711-616454.7) | 2458.7 (2011.4-3056.3) | | 753697.1 (610260.3-952071.2) | 2607 (2110.8-3293.1) | | 0.17 (0.12-0.22) | |
| Malawi | | 204203.3 (169153.8-247171.6) | 4782.5 (3961.7-5788.9) | | 406683.4 (339395.3-493670) | 4519 (3771.3-5485.5) | | -0.27 (-0.32--0.23) | |
| Oman | | 48119.2 (39980.6-58385.9) | 5015.5 (4167.2-6085.6) | | 159913.7 (132129.2-195516.9) | 5110 (4222.2-6247.7) | | 0.02 (0-0.05) | |
| Congo | | 81243.2 (67469.9-98623.6) | 7342.2 (6097.5-8912.9) | | 188317.3 (156565.4-224723.7) | 7037.6 (5851-8398.2) | | -0.19 (-0.28--0.11) | |
| Madagascar | | 284118.3 (233924.3-343582.1) | 5361.6 (4414.4-6483.7) | | 695070.3 (578380.1-839366.7) | 5263.4 (4379.8-6356.1) | | -0.09 (-0.1--0.08) | |
| Papua New Guinea | | 79448 (65940.6-95647.5) | 3997.6 (3317.9-4812.7) | | 197450.3 (163215.7-237527.5) | 3883.2 (3209.9-4671.4) | | -0.14 (-0.15--0.13) | |
| Indonesia | | 2662018.3 (2293094.8-3096425.6) | 2815.6 (2425.4-3275.1) | | 4257926.7 (3679895.9-4915255) | 2981.8 (2577-3442.2) | | 0.19 (0.17-0.22) | |
| New Zealand | | 95842.7 (80533.3-113598.5) | 5310.3 (4462.1-6294.1) | | 109626.1 (95541.5-126670.7) | 5522.3 (4812.8-6380.9) | | 0.1 (0.06-0.14) | |
| Bolivia (Plurinational State of) | | 128284.2 (107454.4-153142.7) | 4296.6 (3598.9-5129.2) | | 243032.6 (205402.8-292503.7) | 3978.7 (3362.7-4788.6) | | -0.32 (-0.36--0.28) | |
| Sao Tome and Principe | | 2114.8 (1728.4-2569.5) | 4227.2 (3454.8-5136.1) | | 4682.3 (3841.8-5674.6) | 4374.2 (3589-5301.2) | | 0.12 (0.09-0.15) | |
| Antigua and Barbuda | | 1143.4 (940.1-1396.7) | 3638.6 (2991.5-4444.7) | | 1813.7 (1506.5-2178) | 3798.1 (3154.7-4560.9) | | 0.07 (0.02-0.13) | |
| Belgium | | 209540.2 (185206.2-241603.5) | 4225.6 (3734.9-4872.2) | | 238406.9 (202932.5-279997.7) | 4759 (4050.9-5589.2) | | 0.65 (0.53-0.77) | |
| Nauru | | 191 (151.5-238.4) | 3844.8 (3050.2-4798.7) | | 216.8 (173.9-269.6) | 3855.8 (3093.3-4795.6) | | 0 (-0.01-0.01) | |
| Burkina Faso | | 188446.6 (156530-226605.1) | 4904.9 (4074.1-5898.1) | | 489820.2 (406151.7-588348.8) | 4783.4 (3966.3-5745.6) | | 0.25 (0.15-0.36) | |
| Bosnia and Herzegovina | | 98989.1 (83785.1-118810.8) | 4076.9 (3450.7-4893.2) | | 52214.6 (43243.2-63794.8) | 3439.7 (2848.7-4202.5) | | -0.86 (-0.95--0.77) | |
| Bulgaria | | 149591.2 (125639.1-180366.6) | 3598.3 (3022.1-4338.6) | | 101749.3 (84911-125251) | 3357.9 (2802.2-4133.4) | | -0.37 (-0.42--0.31) | |
| Democratic Republic of the Congo | | 1165735.5 (956824.9-1415056.5) | 6958.2 (5711.2-8446.3) | | 2801910.7 (2317097.9-3359613.9) | 6714.5 (5552.7-8051) | | -0.17 (-0.19--0.14) | |
| Norway | | 86835.6 (74650.6-100577.5) | 4008.2 (3445.7-4642.5) | | 111468.3 (95999.7-129953.8) | 4474.7 (3853.8-5216.8) | | 0.21 (0.09-0.33) | |
| Algeria | | 622015.7 (515407.4-754764.9) | 5279.6 (4374.7-6406.4) | | 1215039.6 (1010071.7-1468903.5) | 5418.2 (4504.2-6550.3) | | 0.04 (-0.01-0.09) | |
| Slovenia | | 46600.6 (39543-55474.9) | 4595.3 (3899.3-5470.4) | | 34633.9 (29116.2-41914.2) | 3865 (3249.2-4677.4) | | -0.77 (-0.83--0.71) | |
| Portugal | | 306560.6 (258805-365558.2) | 6131.1 (5176-7311) | | 261835.9 (219934.2-311081.2) | 5581.7 (4688.5-6631.5) | | -0.4 (-0.54--0.25) | |
| Chile | | 399956.6 (356694.4-449734.6) | 5628.6 (5019.8-6329.1) | | 455421.8 (382535-542524.1) | 4952.3 (4159.7-5899.5) | | -0.54 (-0.61--0.47) | |
| Solomon Islands | | 5679.6 (4716.1-6838.3) | 3745.7 (3110.3-4509.9) | | 12184 (10164.5-14732.9) | 3711.3 (3096.1-4487.7) | | -0.05 (-0.05--0.04) | |
| Cabo Verde | | 7445.5 (6120.7-8975.5) | 5059.5 (4159.2-6099.1) | | 17444.1 (14607-20823.8) | 5667.9 (4746.1-6766) | | 0.36 (0.29-0.43) | |
| Czechia | | 208747.5 (177608.1-249095.9) | 4003.3 (3406.1-4777) | | 178549.9 (148428.5-217457.3) | 3717.9 (3090.7-4528.1) | | -0.36 (-0.4--0.32) | |
| Netherlands | | 384341.9 (343259.9-434700.6) | 4741.6 (4234.7-5362.8) | | 349050.6 (292588.2-413968.7) | 4650.4 (3898.1-5515.3) | | -0.13 (-0.2--0.07) | |
| Senegal | | 143175.6 (119459.9-172039.1) | 4386.8 (3660.1-5271.1) | | 322658.5 (269548.4-388534.8) | 4394.7 (3671.3-5291.9) | | -0.02 (-0.09-0.04) | |
| Northern Mariana Islands | | 973.4 (806.9-1189.2) | 3351.4 (2778.3-4094.4) | | 710.2 (591-861.2) | 3467.8 (2885.4-4204.9) | | 0.21 (0.13-0.28) | |
| Tunisia | | 251512.6 (208145.6-302767.1) | 6130.2 (5073.2-7379.5) | | 384120.9 (317325.6-459247.3) | 6360.1 (5254.2-7604) | | 0.09 (0.04-0.13) | |
| Hungary | | 217862.9 (184429.4-259268.7) | 4279.3 (3622.6-5092.6) | | 157261.2 (131167.9-190981.5) | 3542.1 (2954.3-4301.6) | | -0.75 (-0.82--0.67) | |
| Sierra Leone | | 78565.1 (65438-95117.8) | 4780.2 (3981.5-5787.3) | | 203450.5 (168741.5-244093.9) | 4897.1 (4061.7-5875.4) | | 0.05 (0.01-0.09) | |
| Guyana | | 23260.1 (19369.8-28057.5) | 5775.7 (4809.7-6967) | | 26388.6 (21908.8-31545.1) | 6365.1 (5284.6-7608.9) | | 0.42 (0.3-0.54) | |
| Central African Republic | | 94993.1 (77289.1-114073.4) | 7596.6 (6180.8-9122.5) | | 190508.3 (157406.1-227243.5) | 7436.2 (6144.1-8870.1) | | -0.11 (-0.13--0.09) | |
| Germany | | 1621281.1 (1394891.9-1912202.6) | 4065.1 (3497.5-4794.6) | | 1582054 (1344284.9-1866605) | 4407.5 (3745.1-5200.3) | | 0.62 (0.48-0.76) | |
| Kuwait | | 54458.7 (44933.6-66530.2) | 5169.2 (4265.1-6315) | | 162409.3 (134650.3-196410.3) | 5578.7 (4625.2-6746.6) | | 0.2 (0.18-0.23) | |
| Mozambique | | 305340.6 (253516.5-366072.5) | 5451.4 (4526.1-6535.6) | | 729113.2 (603760.3-875982.9) | 5472.3 (4531.5-6574.6) | | 0.02 (0-0.03) | |
| Grenada | | 1501.6 (1250.7-1805.3) | 3841.1 (3199.3-4617.9) | | 2184.6 (1821.4-2604.3) | 4033.2 (3362.6-4808.2) | | 0.17 (0.14-0.21) | |
| Saudi Arabia | | 413651 (342890.3-501495) | 5116.2 (4241-6202.7) | | 1366429 (1131933.3-1633723.6) | 5609.8 (4647.1-6707.1) | | 0.34 (0.31-0.36) | |
| Colombia | | 490095 (411034.8-592148.3) | 2894.8 (2427.8-3497.6) | | 677654.3 (575432-803856.1) | 2721.3 (2310.8-3228.1) | | -0.5 (-0.74--0.26) | |
| Russian Federation | | 2970542.3 (2557651.6-3467451.6) | 4000.5 (3444.5-4669.7) | | 2797651.6 (2411272.2-3254888.8) | 4085.8 (3521.5-4753.6) | | -0.05 (-0.16-0.06) | |
| Cameroon | | 226789.3 (187649.1-272846.4) | 5010 (4145.3-6027.4) | | 731009.2 (609803.1-879737.2) | 5084.5 (4241.5-6119) | | 0.04 (0.03-0.06) | |
| Syrian Arab Republic | | 289095.2 (237413.5-350748.8) | 5142.1 (4222.8-6238.7) | | 394840.9 (328484.6-472278.4) | 5317.1 (4423.5-6359.9) | | 0.08 (0.02-0.15) | |
| Lithuania | | 94266.2 (79697.8-112159.7) | 5149.4 (4353.6-6126.9) | | 60423.2 (51178-71711.9) | 4987.5 (4224.4-5919.3) | | -0.24 (-0.33--0.15) | |
| Albania | | 49696.7 (41111.6-61105.9) | 2907.8 (2405.4-3575.3) | | 39992.3 (33241.7-49221) | 3092.2 (2570.2-3805.7) | | 0.17 (0.11-0.22) | |
| Chad | | 138968.8 (115747.1-168950.8) | 5578.6 (4646.4-6782.2) | | 381963.1 (315735.2-462875.6) | 5586 (4617.5-6769.4) | | 0.01 (-0.03-0.06) | |
| Austria | | 193643.7 (163640.1-229780.4) | 4811.6 (4066.1-5709.6) | | 165509.3 (139123.3-199272.6) | 4101.3 (3447.5-4938) | | -0.68 (-0.76--0.6) | |
| Rwanda | | 196368.3 (163361.6-236201.4) | 6248 (5197.8-7515.4) | | 363005 (298189.8-436390) | 5611.9 (4609.9-6746.4) | | -0.69 (-0.8--0.58) | |
| Belize | | 3254.1 (2691.7-3918) | 3819.9 (3159.6-4599.2) | | 9129.8 (7643.4-10936.2) | 4068 (3405.7-4872.9) | | 0.22 (0.16-0.28) | |
| Finland | | 177535.3 (150862.2-212407.2) | 6875.7 (5842.7-8226.2) | | 130152.6 (112115.9-154531.2) | 5575.1 (4802.5-6619.4) | | -0.85 (-0.96--0.74) | |
| Egypt | | 1275191.4 (1052918.5-1544447.5) | 4751.3 (3923.2-5754.6) | | 2538199.9 (2118559.2-3054498.5) | 4935.5 (4119.5-5939.4) | | 0.09 (-0.01-0.18) | |
| Vanuatu | | 2694.9 (2243.5-3228.7) | 3841.1 (3197.7-4602) | | 5362.9 (4463.6-6479.5) | 3690.4 (3071.6-4458.9) | | -0.18 (-0.19--0.16) | |
| Thailand | | 1075730.3 (900075.9-1305721.7) | 3401.8 (2846.3-4129.1) | | 1277053.1 (1060063.6-1560126.6) | 3578.8 (2970.7-4372.1) | | 0.16 (0.1-0.22) | |
| Togo | | 77978 (64748.5-94534.6) | 4849.8 (4027-5879.5) | | 198044.5 (164084.8-237735.9) | 5039.5 (4175.4-6049.5) | | 0.14 (0.12-0.15) | |
| Spain | | 959255.8 (848621.7-1092667.6) | 4938.9 (4369.3-5625.8) | | 1229350.6 (1077391.3-1404814.1) | 5949.6 (5214.2-6798.8) | | 0.9 (0.65-1.16) | |
| Peru | | 304714.9 (253787.6-370349.2) | 2851.5 (2374.9-3465.7) | | 480347.3 (402201.7-578271.7) | 2694.7 (2256.3-3244) | | -0.31 (-0.37--0.25) | |
| Niue | | 39.8 (31.9-48.8) | 3867.4 (3103-4743.7) | | 30.7 (24.6-37.7) | 3988.2 (3193.5-4907.1) | | 0.11 (0.1-0.12) | |
| Turkey | | 1563190.6 (1377885.6-1807391.5) | 5220.7 (4601.8-6036.3) | | 2373513.7 (2000062.2-2832525.9) | 5198.2 (4380.3-6203.5) | | 0.05 (-0.07-0.18) | |
| Tonga | | 1407.2 (1175.1-1704.2) | 3180.3 (2655.8-3851.5) | | 1590.4 (1317.8-1922.5) | 3253.5 (2695.9-3933) | | 0.06 (0.05-0.08) | |
| Gambia | | 27154 (22264.4-32587) | 6119.2 (5017.3-7343.5) | | 66255.6 (54704.8-80551) | 5945.5 (4908.9-7228.2) | | -0.24 (-0.31--0.18) | |
| Sweden | | 240152.3 (211768.2-274588.2) | 5715.4 (5039.9-6535) | | 249003.2 (219806.8-283710.9) | 5561 (4909-6336.1) | | -0.21 (-0.25--0.17) | |
| Ukraine | | 1248561.6 (1067302.9-1466230.4) | 5006 (4279.3-5878.7) | | 1019000.8 (875733.9-1196506.9) | 4902.9 (4213.6-5757) | | -0.32 (-0.43--0.2) | |
| Estonia | | 42205.2 (35739.1-50198.2) | 5558 (4706.5-6610.6) | | 25653.4 (21477.7-31103.7) | 4409.7 (3691.9-5346.6) | | -1.12 (-1.24--1) | |
| Cyprus | | 15984.3 (13282.3-19231.2) | 3961.5 (3291.8-4766.2) | | 27997 (23369.3-34078.2) | 4059.9 (3388.8-4941.7) | | 0.01 (-0.05-0.07) | |
| Saint Kitts and Nevis | | 907.5 (710.5-1154.7) | 4562.7 (3572.1-5805.3) | | 1527.4 (1220.9-1920.6) | 4859.3 (3884.1-6110.1) | | 0.2 (0.17-0.23) | |
| Palau | | 331.6 (263.5-414.4) | 3861.3 (3067.7-4825) | | 376 (300.7-462.2) | 4053.8 (3242-4982.2) | | 0.11 (0.08-0.14) | |
| Azerbaijan | | 112150.1 (92493.9-137367) | 3053.4 (2518.2-3739.9) | | 180738.3 (149840-223321.1) | 3248.3 (2693-4013.7) | | 0.18 (0.15-0.22) | |
| United Arab Emirates | | 60278.7 (50419.5-73033.2) | 5103.6 (4268.8-6183.5) | | 349677 (281161.4-430071.4) | 5019.7 (4036.1-6173.7) | | -0.25 (-0.37--0.14) | |
| Equatorial Guinea | | 13761.9 (11377.7-16676.2) | 7667.8 (6339.4-9291.6) | | 51967.8 (42785.3-63949.3) | 6944 (5717-8545) | | -0.4 (-0.46--0.35) | |
| Maldives | | 4093.8 (3407.3-4865.5) | 4298.3 (3577.5-5108.5) | | 11242.9 (9415.1-13704.5) | 3529.6 (2955.8-4302.4) | | -0.85 (-0.91--0.79) | |
| Canada | | 713346.1 (618725.1-836101.6) | 4837.3 (4195.6-5669.7) | | 801787.7 (688787.8-950600.6) | 4958.3 (4259.5-5878.5) | | -0.15 (-0.27--0.03) | |
| Montenegro | | 10932.9 (9170.1-13289.1) | 3432.4 (2878.9-4172.1) | | 10332.3 (8581.8-12499.6) | 3491.9 (2900.3-4224.3) | | 0.05 (0.03-0.07) | |
| C么te d'Ivoire | | 244842 (203136.1-300040.5) | 4399.2 (3649.9-5391) | | 579067.8 (480902.2-705126.4) | 4433.6 (3682-5398.8) | | 0 (-0.05-0.04) | |
| United Republic of Tanzania | | 596941.6 (495920.2-720714.8) | 5280.4 (4386.8-6375.3) | | 1376886.2 (1142363.7-1674013.7) | 5164 (4284.4-6278.4) | | -0.14 (-0.17--0.11) | |
| Somalia | | 168692.3 (140795.7-202714.3) | 5430.8 (4532.7-6526.1) | | 499855.1 (413548.2-602623.3) | 5280.6 (4368.8-6366.2) | | -0.08 (-0.1--0.06) | |
| Croatia | | 101191.3 (85827-121264.2) | 4145.5 (3516.1-4967.8) | | 68468.5 (57600-83126) | 3673.8 (3090.6-4460.3) | | -0.56 (-0.62--0.51) | |
| Bahrain | | 20244.9 (16892.5-24273.8) | 6697.8 (5588.7-8030.7) | | 56905.7 (47218.4-68448.5) | 6157.4 (5109.2-7406.3) | | -0.48 (-0.56--0.39) | |
| Puerto Rico | | 68210.1 (57836.3-81381.8) | 3698.7 (3136.2-4412.9) | | 57814.4 (48676.4-69159.6) | 3648 (3071.4-4363.8) | | -0.19 (-0.24--0.13) | |
| Jordan | | 99106.4 (82423.9-119377.8) | 5540.7 (4608-6674) | | 336502.9 (279076.7-402890.9) | 5310.9 (4404.6-6358.7) | | -0.31 (-0.37--0.26) | |
| Ghana | | 337090 (277368.2-409148.3) | 4923.6 (4051.3-5976.1) | | 818208.4 (678802.6-981916.7) | 4926.3 (4087-5912) | | -0.06 (-0.1--0.03) | |
| Greece | | 310821.5 (257440.8-372716.7) | 6155.4 (5098.3-7381.1) | | 290570 (240488.8-349869.8) | 6498.9 (5378.8-7825.2) | | 0.08 (-0.23-0.4) | |
| Yemen | | 345421.7 (283075.8-423941.8) | 6337.5 (5193.6-7778.1) | | 991574.8 (816190.5-1189304.8) | 6339.5 (5218.2-7603.6) | | -0.01 (-0.02-0.01) | |
| Guatemala | | 148648.4 (123534-177497.9) | 4284.1 (3560.3-5115.5) | | 401671 (330657.2-481469) | 4197.3 (3455.2-5031.1) | | -0.24 (-0.35--0.13) | |
| Seychelles | | 1202.2 (1008.8-1457.8) | 3245 (2723.1-3935.1) | | 1788.1 (1481.2-2195.7) | 3300.1 (2733.8-4052.4) | | 0.02 (0-0.04) | |
| Guinea-Bissau | | 21625.2 (17983.9-26047.2) | 4888.8 (4065.6-5888.5) | | 47289.4 (39129.1-57009.5) | 4997.5 (4135.1-6024.7) | | 0.08 (0.06-0.1) | |
| Costa Rica | | 56678.1 (47551.8-67907.3) | 3678.4 (3086.1-4407.2) | | 100097 (84626.6-120766.4) | 4011 (3391-4839.2) | | 0.31 (0.3-0.33) | |
| Zambia | | 163564.8 (135955.7-199818.2) | 4630.8 (3849.2-5657.2) | | 423868.1 (354580.2-517828.6) | 4695.2 (3927.7-5736.1) | | -0.03 (-0.06-0.01) | |
| United Kingdom | | 1725697.8 (1476494.4-2011646.3) | 6059.7 (5184.6-7063.7) | | 1692355.2 (1457693.7-1970569.1) | 5552.1 (4782.2-6464.8) | | -0.29 (-0.41--0.17) | |
| Denmark | | 153796.6 (131551.7-179508.7) | 5754.9 (4922.6-6717.1) | | 114469.2 (96644.2-135396.4) | 4465.4 (3770.1-5281.8) | | -1.01 (-1.12--0.9) | |
| Switzerland | | 213530.8 (181915.3-248638.7) | 5885.6 (5014.2-6853.3) | | 195836.1 (168306.2-229078) | 4903.3 (4214-5735.6) | | -0.77 (-1.04--0.49) | |
| Republic of Moldova | | 94651.8 (79717.2-113974.4) | 4304.9 (3625.7-5183.7) | | 72090.7 (60462.8-87571.9) | 3924.1 (3291.2-4766.8) | | -0.41 (-0.49--0.34) | |
| France | | 1625388.6 (1467822.1-1822422.5) | 5571.7 (5031.5-6247.1) | | 1438728.1 (1218442.8-1704240.6) | 5060 (4285.3-5993.8) | | -0.36 (-0.44--0.28) | |
| Gabon | | 31747.8 (26273.4-38400.1) | 7134.8 (5904.5-8629.9) | | 64522.5 (53306.7-77613.2) | 6985.2 (5771-8402.4) | | -0.13 (-0.17--0.09) | |
| Djibouti | | 11304.3 (9373.5-13637.8) | 4837.7 (4011.4-5836.3) | | 33546.1 (27919-40796.1) | 5223.9 (4347.6-6352.9) | | 0.24 (0.23-0.26) | |
| Brunei Darussalam | | 3018.7 (2498.8-3674.8) | 2063.7 (1708.3-2512.3) | | 5503 (4577.6-6655.8) | 2068.1 (1720.4-2501.4) | | -0.06 (-0.12--0.01) | |
| American Samoa | | 784.6 (654-955.2) | 3220.2 (2684.4-3920.4) | | 877.9 (733.8-1064.9) | 3197.5 (2672.6-3878.6) | | -0.05 (-0.07--0.03) | |
| Sri Lanka | | 403188.1 (341915.1-480373.4) | 4386.5 (3719.9-5226.2) | | 404319.3 (341721.8-487650.3) | 3708.1 (3134-4472.3) | | -0.77 (-0.9--0.64) | |
| Burundi | | 149407 (124463.6-179771.6) | 6164.5 (5135.4-7417.4) | | 286716.3 (239089.4-345510.7) | 5231.2 (4362.3-6304) | | -0.76 (-0.84--0.68) | |
| Iraq | | 392951.6 (329971.1-471944.3) | 5024.2 (4219-6034.2) | | 1187599.3 (992270.6-1408371.5) | 5137.7 (4292.6-6092.7) | | 0.01 (-0.3-0.33) | |
| Dominican Republic | | 161514.6 (133365.8-195560.3) | 4439.5 (3665.8-5375.3) | | 262239.5 (218297-314777.2) | 4545 (3783.4-5455.6) | | 0.01 (-0.03-0.05) | |
| Guinea | | 127798.2 (106511.4-153764.8) | 4868.7 (4057.8-5858) | | 279415.2 (230738.1-337967.7) | 4883.2 (4032.5-5906.5) | | 0.02 (0.01-0.03) | |
| Afghanistan | | 301636.9 (251357.6-361517.1) | 6331.2 (5275.9-7588.1) | | 1142334.3 (950169.9-1368891.6) | 6276.9 (5221-7521.8) | | -0.01 (-0.05-0.04) | |
| North Macedonia | | 33786.4 (28376.8-41356.5) | 3228.1 (2711.2-3951.4) | | 34689.9 (29006.6-42748.5) | 3200.3 (2676-3943.7) | | -0.12 (-0.16--0.08) | |
| Honduras | | 68672.8 (56780-83026.5) | 3354.4 (2773.4-4055.5) | | 180256.4 (149758.1-217350.3) | 3483.4 (2894-4200.2) | | 0.17 (0.15-0.19) | |
| Bangladesh | | 2844515.8 (2369360.5-3401800.7) | 5638.3 (4696.5-6742.9) | | 4719760.5 (3969527.9-5660281.7) | 5477.6 (4606.9-6569.1) | | -0.11 (-0.16--0.07) | |
| Tokelau | | 26.6 (21.3-33.1) | 3840.1 (3070.7-4768.6) | | 25.4 (20.3-31.2) | 3932.9 (3139.6-4841.3) | | 0.05 (0.02-0.07) | |
| Lesotho | | 51454.5 (42926.7-61579.4) | 6231.2 (5198.5-7457.3) | | 73111.2 (60594.9-86994.9) | 6373.5 (5282.4-7583.8) | | 0.29 (0.2-0.37) | |
| Uganda | | 540390.6 (448645.2-646090.6) | 7268.5 (6034.5-8690.2) | | 1319375 (1092779.7-1582909.6) | 6987.9 (5787.8-8383.7) | | -0.56 (-0.9--0.23) | |
| Argentina | | 535127.5 (448341.6-634776.9) | 3365.3 (2819.5-3991.9) | | 759956.3 (672506.4-865202.9) | 3277 (2899.9-3730.8) | | -0.16 (-0.24--0.09) | |
| Tuvalu | | 178.8 (142.1-223.1) | 3965.8 (3151.3-4948) | | 232.8 (186.4-287.4) | 3857.1 (3089.8-4763.4) | | -0.14 (-0.15--0.13) | |
| Barbados | | 5142.4 (4294.6-6179.7) | 3816.8 (3187.6-4586.8) | | 5608.3 (4701.8-6727.6) | 4000.6 (3354-4799.1) | | 0.18 (0.15-0.2) | |
| San Marino | | 625.4 (503.7-782.8) | 5208.9 (4195.4-6519.8) | | 817.1 (654.4-1024.4) | 5398.2 (4323.7-6768.2) | | 0.14 (0.12-0.16) | |
| Comoros | | 9922.9 (8239.7-12008.2) | 4847.1 (4024.8-5865.7) | | 18010.6 (14963.8-21837.3) | 4821 (4005.5-5845.3) | | -0.08 (-0.11--0.04) | |
| Botswana | | 28421.5 (23513.5-34427.7) | 4717.3 (3902.7-5714.2) | | 67599.8 (56314.8-81464.7) | 5110.4 (4257.2-6158.5) | | 0.2 (0.15-0.24) | |
| Sudan | | 537297.9 (445265.5-653418.3) | 5901.6 (4890.7-7177.1) | | 1197674.5 (1003527.6-1433637) | 5746.4 (4814.9-6878.5) | | -0.14 (-0.16--0.11) | |

| **TableS4. Incidence of depression in working-age individuals between 1990 and 2019 in 204 countries** | | | | | |
| --- | --- | --- | --- | --- | --- |
| **Location** | **1990** | | **2019** | |  |
|  | **Number** | **ASIR** | **Number** | **ASIR** | **EAPC** |
| Mexico | 1648247.9 (1374588.7-1939203.6) | 3877.2 (3233.5-4561.6) | 3520979.3 (2948672.5-4151559.5) | 5311.7 (4448.4-6263) | 1.42 (1.26-1.58) |
| Haiti | 161449.3 (126981.4-201465.8) | 5510.2 (4333.9-6876) | 344782.5 (272057-430272.3) | 5261.6 (4151.8-6566.2) | -0.18 (-0.2--0.15) |
| Viet Nam | 846730 (683785.5-1043545.9) | 2580.8 (2084.1-3180.7) | 1202194 (977007.3-1448868.7) | 2267.3 (1842.6-2732.6) | -0.48 (-0.51--0.44) |
| Bhutan | 14944.3 (12075.7-18399.5) | 4930 (3983.7-6069.9) | 21450.1 (16970.3-26668) | 4923.9 (3895.6-6121.7) | -0.21 (-0.3--0.11) |
| Jamaica | 45905.8 (35816.8-58112.9) | 3943.9 (3077.1-4992.6) | 62907.3 (49454.5-77770.1) | 4108.7 (3230.1-5079.5) | 0.08 (0-0.16) |
| Nicaragua | 78979.4 (62470.9-97696.3) | 4581.3 (3623.7-5667) | 160936.9 (128993.8-196878.5) | 4592.5 (3681-5618.2) | -0.08 (-0.17-0.02) |
| Kyrgyzstan | 85506.7 (68142-106048.1) | 4080.7 (3252-5061.1) | 131572.3 (106957.3-160325.1) | 3917.2 (3184.3-4773.2) | -0.17 (-0.19--0.14) |
| Georgia | 93073.9 (75142.5-114742.4) | 3470.1 (2801.6-4278) | 60626.8 (49102.1-73777.5) | 3665.3 (2968.6-4460.4) | 0.2 (0.17-0.24) |
| Lebanon | 99990.8 (79988.1-122018.9) | 6635.3 (5308-8097.1) | 192545.1 (152451.9-237351.8) | 7205.6 (5705.2-8882.5) | 0.31 (0.14-0.47) |
| Kazakhstan | 330368.4 (269374.5-401738.7) | 4017.9 (3276.1-4885.9) | 394692.5 (324398.6-479902.2) | 4252 (3494.7-5169.9) | 0.37 (0.28-0.46) |
| Namibia | 27466.5 (21971.8-34052.3) | 4173.1 (3338.3-5173.7) | 52164.3 (41855.8-64274.2) | 4155.8 (3334.5-5120.6) | -0.19 (-0.32--0.06) |
| Republic of Korea | 685368.6 (578191.4-806114.3) | 2648.2 (2234-3114.7) | 723887.9 (619465.7-837362.8) | 2779.2 (2378.3-3214.9) | 0.27 (0.12-0.42) |
| Timor-Leste | 14017.8 (11273.3-17108.2) | 3663.4 (2946.2-4471.1) | 19463.2 (15450.1-24122.5) | 2982 (2367.1-3695.8) | -0.87 (-0.98--0.76) |
| China | 23206347.5 (19507637.1-27341422.8) | 3471.6 (2918.3-4090.2) | 17943053.6 (15225607.2-20896343) | 2489.6 (2112.5-2899.3) | -1.57 (-1.77--1.37) |
| Eritrea | 80247.7 (63926.4-99379.8) | 5886.8 (4689.5-7290.3) | 194633.6 (153271.8-239203.4) | 5634.3 (4436.9-6924.5) | -0.17 (-0.2--0.13) |
| Iceland | 5800.2 (4796.3-6998.1) | 4379.3 (3621.3-5283.8) | 6103.9 (5014.7-7383) | 3739.7 (3072.4-4523.5) | -0.69 (-0.78--0.61) |
| Panama | 47199.7 (37630.6-58041.7) | 3834.3 (3057-4715.1) | 81919.2 (65666-99574.7) | 3897.7 (3124.4-4737.8) | 0.06 (0.03-0.1) |
| Serbia | 166801.9 (137206.6-200844.4) | 3631.3 (2987-4372.4) | 125228.2 (101713.6-151657.1) | 3128.7 (2541.2-3789) | -0.65 (-0.71--0.59) |
| India | 22274565.2 (18539395.9-26486767.3) | 5302.7 (4413.5-6305.5) | 33277196.5 (27928639.3-39352621.6) | 4380.9 (3676.8-5180.8) | -1.52 (-1.92--1.13) |
| Libya | 122333.3 (96957.4-152320.3) | 6322 (5010.6-7871.7) | 281612.2 (225034.6-345039.9) | 6855.2 (5477.9-8399.2) | 0.26 (0.25-0.28) |
| South Africa | 1054804.3 (886551-1250511.9) | 5539.4 (4655.8-6567.2) | 1724868.3 (1451632.9-2032643.6) | 5664.3 (4767-6675) | 0.26 (0.09-0.43) |
| Democratic People's Republic of Korea | 297356.1 (239218.1-358660) | 2786.9 (2242-3361.4) | 338918.4 (276901.5-416312.8) | 2435.6 (1989.9-2991.7) | -0.56 (-0.61--0.52) |
| Uruguay | 56239.4 (46188.1-68030) | 3785.1 (3108.7-4578.7) | 72810.7 (59629.8-88284.8) | 4417.6 (3617.9-5356.5) | 0.84 (0.73-0.94) |
| Japan | 1935676.9 (1666823.6-2231041.3) | 2979.8 (2565.9-3434.5) | 1675454.5 (1439994-1945709.2) | 3237.2 (2782.3-3759.4) | 0.56 (0.35-0.77) |
| Poland | 472227.3 (387345-572232) | 2488.3 (2041-3015.3) | 456409.9 (375685-551741.9) | 2507.8 (2064.2-3031.6) | -0.55 (-0.72--0.38) |
| Saint Vincent and the Grenadines | 2327.1 (1838.6-2858.9) | 4362.6 (3446.9-5359.6) | 2742.5 (2207.2-3337.2) | 4795.9 (3859.8-5835.9) | 0.37 (0.33-0.42) |
| Australia | 656521.7 (556442.4-767712.4) | 7305.5 (6191.9-8542.8) | 872971.6 (716992.9-1061979.9) | 7565.7 (6213.9-9203.8) | 0.14 (-0.03-0.32) |
| Cook Islands | 363.5 (267.8-479.4) | 3840.2 (2828.9-5064.4) | 315.6 (236.5-410.4) | 3865.4 (2896.4-5026.6) | -0.01 (-0.03-0.01) |
| Liberia | 47381.2 (37370.9-58924.6) | 5797.9 (4573-7210.5) | 137311.7 (109354.2-168127.2) | 5551.5 (4421.2-6797.4) | 0.52 (0.31-0.74) |
| Greenland | 4327.9 (3532.2-5322.1) | 12939.1 (10560.5-15911.5) | 2986.5 (2411.9-3624.4) | 11234.1 (9072.7-13633.6) | -0.48 (-0.52--0.44) |
| Tajikistan | 75785.9 (60359.6-93799.2) | 3142.5 (2502.9-3889.5) | 145401.8 (116418.2-179558.8) | 2926.2 (2342.9-3613.6) | -0.33 (-0.39--0.26) |
| Fiji | 13253.9 (10714.2-16207.3) | 3352.5 (2710.1-4099.5) | 15591.7 (12578.5-19132.5) | 3344.7 (2698.3-4104.2) | -0.07 (-0.1--0.05) |
| Bermuda | 1751.8 (1409.2-2143.6) | 5176.1 (4163.9-6333.8) | 1214.1 (972.4-1488.9) | 4376.7 (3505.6-5367.4) | -0.69 (-0.74--0.63) |
| Israel | 148329.2 (120889.7-180571.3) | 6109.2 (4979.1-7437.2) | 252203.5 (204015.1-302118.2) | 5762.7 (4661.6-6903.2) | -0.56 (-0.79--0.34) |
| United States Virgin Islands | 2485.5 (1991.8-3053) | 4529.2 (3629.6-5563.4) | 2054 (1636.2-2522.1) | 4729.2 (3767.1-5806.7) | 0.18 (0.15-0.22) |
| Pakistan | 2434784.3 (1954415.4-2973928.2) | 4908.2 (3939.9-5995.1) | 5394557.8 (4289510.4-6589997.5) | 4826.3 (3837.7-5895.9) | -0.13 (-0.19--0.06) |
| Guam | 3163.9 (2556.4-3879.7) | 4093.8 (3307.8-5019.9) | 3317.9 (2710.1-4032) | 4090.6 (3341.2-4971) | 0.05 (-0.03-0.13) |
| Mauritania | 37968.8 (30277.9-46309.8) | 4163.1 (3319.8-5077.6) | 75840.8 (60230.6-93770.4) | 3914.9 (3109.1-4840.5) | -0.31 (-0.4--0.22) |
| Cambodia | 181623.4 (143487.2-226697.6) | 3953.9 (3123.7-4935.2) | 284295.6 (231247.7-350160.9) | 3235.4 (2631.7-3985) | -0.86 (-0.92--0.79) |
| Singapore | 83810.6 (72097.7-98028.9) | 4407.6 (3791.7-5155.4) | 86032.3 (71927.8-101856.1) | 2797.1 (2338.5-3311.5) | -1.97 (-2.19--1.75) |
| Nepal | 521660.6 (422004.8-633101.1) | 5864 (4743.8-7116.7) | 1001479.6 (813559-1206443.1) | 6188.5 (5027.3-7455.1) | 0.33 (0.19-0.47) |
| South Sudan | 147732.1 (116662.9-182626.9) | 5402 (4265.9-6678) | 236174.5 (188867.7-290014.8) | 5601.3 (4479.4-6878.2) | 0.11 (0.09-0.13) |
| Slovakia | 88698.5 (72658.9-107814.2) | 3319.4 (2719.2-4034.8) | 78257.6 (62561.2-94429.9) | 2974.2 (2377.7-3588.8) | -0.56 (-0.73--0.39) |
| Mongolia | 53909.1 (42954.6-67232.3) | 5254.2 (4186.5-6552.8) | 95382.7 (76952.1-116262.6) | 5276.6 (4257-6431.6) | 0 (-0.03-0.03) |
| Italy | 1478109.3 (1204067.6-1782867.7) | 5146.1 (4192-6207.1) | 1270912.5 (1039757.2-1522854.3) | 5011.6 (4100.1-6005.1) | -0.66 (-0.86--0.46) |
| Kiribati | 1433.4 (1130.4-1811.6) | 3909 (3082.6-4940.1) | 2094.8 (1684.5-2597.2) | 3466.7 (2787.8-4298.1) | -0.43 (-0.49--0.37) |
| Ireland | 106231 (89350.1-127001.6) | 5962.9 (5015.4-7128.8) | 148689.1 (123407.1-177329.9) | 6376.9 (5292.6-7605.2) | 0.05 (-0.05-0.14) |
| Andorra | 1670.1 (1357.4-2054) | 5152.5 (4187.7-6336.6) | 2118.9 (1700.3-2627.1) | 5173.3 (4151.4-6414) | -0.02 (-0.06-0.01) |
| Kenya | 585894.4 (485626.7-694289.3) | 5772.5 (4784.6-6840.5) | 1431559.6 (1194491-1687576.3) | 5495.9 (4585.8-6478.8) | -0.26 (-0.36--0.17) |
| Mali | 145812.2 (116944.1-178015) | 4035 (3236.1-4926.1) | 366525 (290336.9-453603.5) | 3795.7 (3006.7-4697.5) | -0.32 (-0.43--0.2) |
| Morocco | 990057.4 (783766.6-1223312) | 7952.5 (6295.5-9826.1) | 1516919.8 (1214171-1878873) | 7913.1 (6333.8-9801.2) | -0.04 (-0.06--0.03) |
| Romania | 337352.3 (273406.4-405009.6) | 2974.4 (2410.6-3570.9) | 255215.9 (207693.2-310217.3) | 2947.6 (2398.7-3582.8) | -0.04 (-0.08--0.01) |
| Zimbabwe | 147588.7 (116656.2-182780.4) | 3205.6 (2533.8-3970) | 252946.1 (201593-312115.1) | 3362.7 (2680-4149.3) | 0.15 (0.05-0.25) |
| Eswatini | 17517.5 (13820.6-21816) | 4920.4 (3882-6127.8) | 33497.6 (27000.8-41185.6) | 5530.7 (4458.1-6800.1) | 0.43 (0.38-0.48) |
| United States of America | 7236205.9 (6213595.3-8490644.6) | 5406 (4642-6343.2) | 10867823.4 (9331179.9-12667233.1) | 7218.5 (6197.8-8413.7) | 0.69 (0.33-1.05) |
| Turkmenistan | 62843.3 (50560.5-77804.9) | 3569.7 (2872-4419.5) | 87677.2 (70881-107139.1) | 3327.3 (2689.9-4065.9) | -0.28 (-0.33--0.23) |
| Venezuela (Bolivarian Republic of) | 396129.6 (316351.6-491569.6) | 4128.1 (3296.7-5122.7) | 638513.4 (512009.6-775488.1) | 4436.2 (3557.3-5387.9) | 0.17 (0.13-0.2) |
| Marshall Islands | 717.4 (566.5-899.3) | 3564 (2814.3-4467.7) | 1007.4 (802.5-1244.8) | 3326.9 (2650.3-4110.8) | -0.27 (-0.3--0.25) |
| Trinidad and Tobago | 38412.7 (31135.8-48367.9) | 6205.6 (5030-7813.9) | 40604 (32800-49082.7) | 5845.6 (4722.1-7066.3) | -0.27 (-0.32--0.22) |
| Taiwan (Province of China) | 237798.7 (194245.3-285402.3) | 2112.7 (1725.8-2535.6) | 284584.8 (235592.8-342472.2) | 2435.3 (2016.1-2930.7) | 0.57 (0.51-0.64) |
| Angola | 434859.8 (342011.2-544221.7) | 9299.2 (7313.7-11637.8) | 1177137.5 (911676.2-1482837.4) | 8700.7 (6738.6-10960.3) | -0.29 (-0.32--0.26) |
| Palestine | 87297.9 (69432.8-109011.8) | 9885.4 (7862.4-12344.2) | 251431.7 (199174.1-315641.6) | 9912.6 (7852.3-12444) | 0.15 (-0.12-0.43) |
| Suriname | 13688.6 (11139.3-16890.1) | 6962.1 (5665.5-8590.4) | 21562.1 (17435.8-26251.4) | 7481.4 (6049.7-9108.4) | 0.35 (0.28-0.42) |
| Saint Lucia | 2959.7 (2351-3627.8) | 4448 (3533.2-5452.1) | 4398.9 (3541.8-5384.4) | 4699 (3783.4-5751.7) | 0.19 (0.15-0.23) |
| Niger | 164356.7 (131255.6-203358.5) | 4913.6 (3924-6079.6) | 453350.6 (362955.6-555208.4) | 4784.4 (3830.4-5859.3) | -0.11 (-0.13--0.09) |
| Bahamas | 5821.7 (4646.9-7169.1) | 4087.7 (3262.8-5033.8) | 8476.3 (6721.6-10423.5) | 4140.5 (3283.3-5091.6) | -0.02 (-0.08-0.04) |
| Ethiopia | 1382137.3 (1118927.9-1686353.9) | 6247.7 (5057.9-7622.8) | 2807674.8 (2261317.7-3422371.9) | 5347.7 (4307.1-6518.5) | -0.71 (-0.86--0.56) |
| Micronesia (Federated States of) | 1722.2 (1375.7-2148.4) | 3639.6 (2907.3-4540.3) | 1785 (1444.1-2208.7) | 3332.7 (2696.2-4123.6) | -0.38 (-0.41--0.34) |
| Lao People's Democratic Republic | 64072.7 (51293.1-79312.7) | 3447 (2759.5-4266.9) | 117197.7 (95527.1-143444) | 3007.9 (2451.7-3681.5) | -0.6 (-0.73--0.46) |
| Belarus | 254993.4 (205474.1-310142.8) | 5047.8 (4067.5-6139.5) | 225550.3 (180875.2-275101.4) | 5176.5 (4151.2-6313.8) | 0.03 (-0.09-0.15) |
| Malta | 7859.5 (6333-9605.9) | 4104 (3306.9-5015.9) | 7979.3 (6422.2-9615.1) | 4050.3 (3259.9-4880.7) | -0.19 (-0.28--0.11) |
| Samoa | 2793 (2203.2-3499.9) | 3575.5 (2820.4-4480.3) | 3271.4 (2649.4-4052.9) | 3146.3 (2548.1-3897.9) | -0.56 (-0.62--0.5) |
| Brazil | 4634712.3 (3887561.4-5504629.1) | 6046.9 (5072.1-7181.9) | 6287672.6 (5413601.8-7242406.4) | 5444.4 (4687.6-6271.1) | -0.57 (-0.96--0.17) |
| Dominica | 1394.4 (1105.9-1712.9) | 3950.6 (3133.2-4853.2) | 1424.8 (1133.4-1757.5) | 4194.9 (3336.9-5174.7) | 0.22 (0.17-0.27) |
| Latvia | 74412.8 (60379.3-92638) | 5788.6 (4697-7206.4) | 40810.4 (33936.4-48714.7) | 4981.9 (4142.7-5946.8) | -0.68 (-0.76--0.6) |
| Uzbekistan | 360906.9 (290754.6-445587.5) | 3688.3 (2971.4-4553.7) | 648497.7 (526559.3-797485.3) | 3586.8 (2912.4-4410.9) | -0.12 (-0.15--0.09) |
| Philippines | 1103767.3 (890610-1341112.5) | 3542.1 (2858.1-4303.8) | 1714352.7 (1392123.2-2082902.3) | 2932.5 (2381.3-3563) | -0.72 (-0.96--0.47) |
| Luxembourg | 11128.5 (9200.7-13552.7) | 5593.8 (4624.8-6812.4) | 13204.9 (11216.9-15469) | 4303.1 (3655.3-5040.9) | -1.09 (-1.17--1.02) |
| Mauritius | 35377.6 (28809.2-44146.3) | 5817.4 (4737.3-7259.3) | 32523.6 (26053.8-39536.9) | 5003.1 (4007.8-6081.9) | -0.62 (-0.74--0.5) |
| Paraguay | 94119 (74694.6-117580.2) | 4980.2 (3952.4-6221.6) | 201066.1 (160721.4-245978.4) | 5407.8 (4322.7-6615.7) | 0.3 (0.19-0.41) |
| Benin | 103240.5 (82178.9-127516.8) | 5156.1 (4104.2-6368.5) | 302170.7 (241877.6-370998.2) | 5188.5 (4153.2-6370.3) | 0.01 (0-0.03) |
| Malaysia | 324655.6 (258886.1-396737.3) | 3605.3 (2875-4405.8) | 770633.6 (637034.3-922577.6) | 4414.6 (3649.3-5285) | 0.99 (0.76-1.23) |
| Ecuador | 226154.1 (183078.3-280733.3) | 4568.1 (3698-5670.5) | 413340.8 (331156.8-505773.6) | 4496.4 (3602.4-5501.9) | -0.08 (-0.12--0.05) |
| Monaco | 831.6 (630.7-1095) | 6038.5 (4579.9-7951.4) | 857.1 (648.3-1113.4) | 6013.2 (4548.7-7811.8) | 0.01 (-0.01-0.02) |
| Qatar | 19502.9 (15648-24200) | 6679.4 (5359.1-8288) | 132376.5 (104526.3-165900.5) | 6130.6 (4840.8-7683.1) | -0.55 (-0.67--0.42) |
| El Salvador | 143725.6 (116454-179048.4) | 5752.9 (4661.3-7166.8) | 164493 (131868.3-203324.8) | 5013.7 (4019.3-6197.2) | -0.58 (-0.63--0.53) |
| Armenia | 48862 (39129-59900) | 2861.1 (2291.2-3507.4) | 47312.5 (38063.7-58317.4) | 3201.8 (2575.9-3946.6) | 0.52 (0.47-0.57) |
| Iran (Islamic Republic of) | 2025126.6 (1581705.1-2528988.2) | 7660.1 (5982.8-9565.9) | 3892758.1 (3092144.3-4828898.8) | 8234.5 (6540.9-10214.7) | 0.65 (0.46-0.83) |
| Cuba | 471246 (384523.2-578113.2) | 7673.9 (6261.7-9414.2) | 280241.4 (229076.7-338119.4) | 5280.3 (4316.3-6370.9) | -1.53 (-1.68--1.38) |
| Nigeria | 2076702.3 (1669979.3-2530752.4) | 5067.9 (4075.4-6176) | 4088184.8 (3316722-4960889.4) | 4052.6 (3287.8-4917.7) | -0.91 (-1.43--0.39) |
| Myanmar | 358605.8 (289489.1-436495.8) | 1777.9 (1435.2-2164.1) | 510152.9 (410796.3-620963.4) | 1764.6 (1420.9-2147.9) | -0.11 (-0.25-0.04) |
| Malawi | 205065.8 (164167.2-251495.5) | 4802.7 (3844.9-5890.1) | 398520.8 (319904.1-490384.1) | 4428.3 (3554.7-5449) | -0.42 (-0.5--0.34) |
| Oman | 55196 (43878.2-68553.9) | 5753.1 (4573.4-7145.4) | 180904.7 (141730.5-226805.5) | 5780.8 (4529-7247.5) | -0.05 (-0.08--0.02) |
| Congo | 100540.9 (78986.4-127179.9) | 9086.2 (7138.3-11493.7) | 224661.8 (180004.7-279156.6) | 8395.9 (6727-10432.4) | -0.35 (-0.45--0.24) |
| Madagascar | 302390 (239038.7-378372.9) | 5706.4 (4510.9-7140.3) | 727396.5 (576179.6-890956.2) | 5508.2 (4363.1-6746.8) | -0.16 (-0.18--0.14) |
| Papua New Guinea | 82092.6 (65416-102735.3) | 4130.7 (3291.5-5169.3) | 194870.8 (156258.3-243180.3) | 3832.5 (3073.1-4782.6) | -0.3 (-0.32--0.29) |
| Indonesia | 2191392.9 (1780202.1-2654999.1) | 2317.8 (1882.9-2808.2) | 3309970.5 (2714693.4-3987976.5) | 2318 (1901.1-2792.8) | -0.08 (-0.14--0.02) |
| New Zealand | 106994.6 (84779.5-131678.6) | 5928.2 (4697.3-7295.8) | 122933.2 (101859.2-146160.7) | 6192.6 (5131.1-7362.7) | 0.1 (0.06-0.14) |
| Bolivia (Plurinational State of) | 148931.2 (118717.8-183531.4) | 4988.1 (3976.2-6147) | 270788.2 (217099.1-332122.8) | 4433.1 (3554.1-5437.2) | -0.48 (-0.52--0.43) |
| Sao Tome and Principe | 2123.4 (1650.8-2650.5) | 4244.5 (3299.7-5297.9) | 4640 (3653-5779.5) | 4334.8 (3412.7-5399.3) | 0.04 (0.01-0.08) |
| Antigua and Barbuda | 1237.4 (955.3-1552) | 3937.7 (3040.1-4938.8) | 1947.8 (1521.7-2408.9) | 4078.7 (3186.5-5044.4) | 0.03 (-0.05-0.11) |
| Belgium | 224155.5 (199955.8-251038.1) | 4520.3 (4032.3-5062.5) | 265025.7 (216964.1-317584.8) | 5290.3 (4331-6339.5) | 0.85 (0.69-1.02) |
| Nauru | 189.2 (140.2-252.1) | 3808.2 (2821.6-5075.2) | 214.6 (159.9-283.5) | 3817.5 (2844.8-5043.4) | 0 (-0.01-0.02) |
| Burkina Faso | 199573.6 (159591.8-243388.8) | 5194.5 (4153.8-6334.9) | 511732.5 (407715.8-624485.1) | 4997.4 (3981.6-6098.5) | 0.35 (0.21-0.5) |
| Bosnia and Herzegovina | 100976.1 (82723.5-122344.6) | 4158.7 (3407-5038.8) | 46850 (37379.9-57593.4) | 3086.3 (2462.4-3794) | -1.42 (-1.56--1.28) |
| Bulgaria | 140595 (116262-169638.2) | 3381.9 (2796.6-4080.5) | 87968.2 (72008-108235.8) | 2903.1 (2376.4-3571.9) | -0.77 (-0.85--0.68) |
| Democratic Republic of the Congo | 1414677.2 (1118698.6-1783168.4) | 8444.1 (6677.4-10643.6) | 3362854.2 (2667181.1-4236857.1) | 8058.7 (6391.6-10153.2) | -0.21 (-0.24--0.18) |
| Norway | 87400.7 (71716.3-105086.7) | 4034.2 (3310.3-4850.6) | 117204.8 (95445.1-141286.9) | 4705 (3831.5-5671.7) | 0.36 (0.19-0.52) |
| Algeria | 730522.6 (573283.7-912887.5) | 6200.6 (4866-7748.5) | 1382862.3 (1090100.1-1711710.4) | 6166.6 (4861.1-7633.1) | -0.09 (-0.14--0.04) |
| Slovenia | 49567.6 (40777.5-60310.6) | 4887.9 (4021.1-5947.2) | 32859.4 (26653.5-39547.4) | 3666.9 (2974.4-4413.3) | -1.25 (-1.35--1.15) |
| Portugal | 362266.4 (295913-443268.4) | 7245.2 (5918.2-8865.2) | 297457 (238610.3-365547) | 6341.1 (5086.6-7792.6) | -0.57 (-0.75--0.39) |
| Chile | 509259.5 (449853.5-578230.3) | 7166.8 (6330.8-8137.5) | 557070.9 (454173.3-680929.2) | 6057.7 (4938.8-7404.5) | -0.68 (-0.76--0.6) |
| Solomon Islands | 5865.4 (4642.9-7323.3) | 3868.2 (3062-4829.7) | 11787.2 (9381.5-14609.2) | 3590.4 (2857.6-4450) | -0.33 (-0.35--0.3) |
| Cabo Verde | 8103.6 (6453.6-9938.8) | 5506.6 (4385.4-6753.7) | 19111.5 (15383-23528.7) | 6209.7 (4998.2-7644.9) | 0.4 (0.28-0.51) |
| Czechia | 208703.2 (174280.6-249931.3) | 4002.4 (3342.3-4793.1) | 164312.6 (133069.8-199142.7) | 3421.4 (2770.9-4146.7) | -0.74 (-0.79--0.68) |
| Netherlands | 428444.3 (382946.2-479242.2) | 5285.6 (4724.3-5912.3) | 380203.1 (310232.7-457692.6) | 5065.4 (4133.2-6097.8) | -0.24 (-0.33--0.16) |
| Senegal | 144758 (116041.2-177284.9) | 4435.2 (3555.4-5431.8) | 324757.1 (255783.9-396025.3) | 4423.2 (3483.8-5393.9) | -0.07 (-0.16-0.03) |
| Northern Mariana Islands | 858.2 (698.2-1057.4) | 2955 (2403.8-3640.5) | 637.5 (516.8-774.4) | 3112.8 (2523.4-3781) | 0.19 (0.13-0.25) |
| Tunisia | 304840.6 (241882.4-378928.2) | 7430 (5895.5-9235.8) | 455662 (361952.7-568087) | 7544.7 (5993.1-9406.2) | -0.01 (-0.05-0.04) |
| Hungary | 224409.4 (186058.3-272200.5) | 4407.9 (3654.6-5346.6) | 142219.6 (116278-171046.1) | 3203.3 (2619-3852.5) | -1.28 (-1.38--1.18) |
| Sierra Leone | 81408.1 (64397.6-100800.4) | 4953.2 (3918.2-6133.1) | 215545.8 (171148.6-266318.1) | 5188.3 (4119.6-6410.4) | 0.14 (0.08-0.2) |
| Guyana | 29172 (23590.8-36335.9) | 7243.7 (5857.9-9022.6) | 33308 (26796.9-40841.1) | 8034.2 (6463.6-9851.2) | 0.48 (0.35-0.62) |
| Central African Republic | 117458.4 (90898.5-147096.2) | 9393.2 (7269.2-11763.4) | 234558.6 (184815.9-290412.9) | 9155.7 (7214-11335.9) | -0.14 (-0.16--0.11) |
| Germany | 1663524.3 (1445037.7-1933289.9) | 4171 (3623.2-4847.4) | 1702358 (1397670.6-2039825.4) | 4742.7 (3893.8-5682.8) | 0.93 (0.7-1.17) |
| Kuwait | 62157.2 (49487.1-77438.4) | 5899.9 (4697.3-7350.4) | 183153.8 (145436.7-228030.1) | 6291.2 (4995.7-7832.7) | 0.15 (0.12-0.18) |
| Mozambique | 322524.9 (257411.1-394373.9) | 5758.2 (4595.7-7040.9) | 780269.1 (617524.1-957369.8) | 5856.3 (4634.8-7185.5) | 0.05 (0.03-0.07) |
| Grenada | 1687.6 (1351.6-2086.4) | 4316.9 (3457.3-5337) | 2431.9 (1964.1-2951) | 4489.8 (3626.1-5448.1) | 0.14 (0.09-0.19) |
| Saudi Arabia | 472514.3 (374308.8-585091.3) | 5844.2 (4629.6-7236.6) | 1549107.7 (1233924.6-1905781.8) | 6359.7 (5065.8-7824) | 0.32 (0.3-0.35) |
| Colombia | 512242 (409986.7-631169.3) | 3025.6 (2421.6-3728.1) | 669601.9 (560028-788162.1) | 2689 (2249-3165.1) | -0.75 (-1.07--0.42) |
| Russian Federation | 3021304.8 (2455057.9-3695686.1) | 4068.9 (3306.3-4977.1) | 2776064.6 (2262726.1-3371956.4) | 4054.3 (3304.6-4924.6) | -0.19 (-0.33--0.06) |
| Cameroon | 242881.1 (192164.7-301219.4) | 5365.5 (4245.1-6654.2) | 785725.7 (625434.8-964537.8) | 5465.1 (4350.2-6708.8) | 0.05 (0.02-0.08) |
| Syrian Arab Republic | 338321.9 (264978.8-421619.1) | 6017.7 (4713.1-7499.3) | 456935.1 (358980.2-565017.6) | 6153.3 (4834.2-7608.8) | 0 (-0.09-0.08) |
| Lithuania | 106362.4 (87369.1-127918.2) | 5810.2 (4772.7-6987.7) | 66105.6 (54012.6-80892.3) | 5456.6 (4458.4-6677.1) | -0.37 (-0.48--0.25) |
| Albania | 42682.6 (34370.4-52604.4) | 2497.4 (2011-3077.9) | 34413.5 (28049.8-41765.7) | 2660.8 (2168.8-3229.3) | 0.2 (0.12-0.27) |
| Chad | 155168.8 (123121.2-193594.8) | 6228.9 (4942.4-7771.5) | 430990.7 (343306.9-532429.3) | 6303.1 (5020.7-7786.5) | 0.05 (-0.01-0.12) |
| Austria | 210259.1 (171621.8-251095.6) | 5224.5 (4264.4-6239.2) | 166261.1 (137236.9-200837.7) | 4120 (3400.8-4976.8) | -0.99 (-1.1--0.87) |
| Rwanda | 221979.1 (172588.3-276276.2) | 7062.9 (5491.4-8790.5) | 389143.1 (307474.2-478965) | 6015.9 (4753.4-7404.5) | -1.01 (-1.17--0.85) |
| Belize | 3683.9 (2951.2-4531.3) | 4324.4 (3464.3-5319.1) | 10326 (8302.6-12668.3) | 4601 (3699.5-5644.7) | 0.22 (0.15-0.29) |
| Finland | 206454.5 (172723.6-250724.1) | 7995.7 (6689.3-9710.2) | 141795 (117780.4-169858.8) | 6073.8 (5045.2-7275.9) | -1.17 (-1.33--1.01) |
| Egypt | 1430563.9 (1128075.1-1780139.9) | 5330.3 (4203.2-6632.8) | 2853629.6 (2250711.4-3570502.4) | 5548.8 (4376.5-6942.8) | 0.07 (-0.05-0.19) |
| Vanuatu | 2725.9 (2181.9-3408.2) | 3885.3 (3109.9-4857.8) | 5184.4 (4138.2-6404) | 3567.6 (2847.7-4406.9) | -0.36 (-0.38--0.34) |
| Thailand | 978867.4 (785785.3-1196496.4) | 3095.5 (2484.9-3783.7) | 1065778.3 (867249.7-1289608.4) | 2986.8 (2430.4-3614) | -0.15 (-0.22--0.08) |
| Togo | 82678 (64890.6-102247) | 5142.1 (4035.8-6359.2) | 209277.9 (167095.3-257065.1) | 5325.3 (4252-6541.4) | 0.12 (0.09-0.14) |
| Spain | 1039290.9 (918595.8-1169547.3) | 5351 (4729.6-6021.7) | 1406610.7 (1216407.9-1611910.7) | 6807.5 (5887-7801.1) | 1.18 (0.84-1.53) |
| Peru | 300404.3 (240637.9-370848.9) | 2811.1 (2251.8-3470.3) | 438482.7 (355996.2-531518) | 2459.8 (1997.1-2981.7) | -0.65 (-0.74--0.55) |
| Niue | 39.1 (29.5-51.3) | 3798.4 (2867.4-4989) | 29.3 (22.2-38.6) | 3804.1 (2884.9-5013.8) | -0.01 (-0.02-0.01) |
| Turkey | 1810518 (1570754.6-2075782.2) | 6046.7 (5246-6932.7) | 2655120.4 (2149768.2-3262523.9) | 5814.9 (4708.2-7145.2) | -0.06 (-0.22-0.11) |
| Tonga | 1312.5 (1049.6-1629.8) | 2966.3 (2372.1-3683.3) | 1410.6 (1137.9-1734.5) | 2885.7 (2327.8-3548.4) | -0.17 (-0.21--0.14) |
| Gambia | 31445.1 (24663.8-39385.7) | 7086.2 (5558-8875.6) | 75913.5 (60087.5-95100.4) | 6812.1 (5392-8533.8) | -0.34 (-0.43--0.25) |
| Sweden | 273008.5 (231382.9-320609.5) | 6497.4 (5506.7-7630.2) | 278033.3 (237517.3-326410.9) | 6209.3 (5304.5-7289.8) | -0.29 (-0.35--0.22) |
| Ukraine | 1405662.7 (1140187.7-1699638.6) | 5635.9 (4571.5-6814.6) | 1104741.7 (899804.4-1343040) | 5315.4 (4329.4-6462) | -0.54 (-0.71--0.37) |
| Estonia | 48530.6 (39321.6-60224.5) | 6391 (5178.3-7931) | 26484.2 (21341.2-32894.5) | 4552.5 (3668.4-5654.4) | -1.6 (-1.77--1.44) |
| Cyprus | 15989.6 (12800.6-19646.7) | 3962.8 (3172.5-4869.2) | 27652.9 (21885.8-33744.5) | 4010 (3173.7-4893.4) | -0.06 (-0.14-0.01) |
| Saint Kitts and Nevis | 1076.5 (794.6-1428) | 5412.2 (3995-7179.4) | 1788.2 (1359.6-2362.6) | 5689.2 (4325.4-7516.5) | 0.16 (0.13-0.19) |
| Palau | 327.8 (244.2-431.6) | 3816.4 (2843.7-5024.8) | 346.9 (263.3-452.3) | 3739.8 (2838.4-4876.3) | -0.08 (-0.11--0.06) |
| Azerbaijan | 100553.4 (79357.1-123784.4) | 2737.6 (2160.6-3370.1) | 158239.1 (125901.8-192858.2) | 2844 (2262.8-3466.2) | 0.17 (0.11-0.23) |
| United Arab Emirates | 68679.6 (54991.5-84359.1) | 5814.9 (4655.9-7142.4) | 384345 (298882.1-488462) | 5517.3 (4290.5-7012) | -0.43 (-0.56--0.3) |
| Equatorial Guinea | 17059.4 (13356.8-21308.8) | 9505.1 (7442.1-11872.8) | 63628.8 (50301.9-80244.2) | 8502.2 (6721.4-10722.3) | -0.45 (-0.51--0.39) |
| Maldives | 4437.9 (3548-5483.8) | 4659.5 (3725.2-5757.6) | 9977.6 (8169.5-12165) | 3132.4 (2564.7-3819.1) | -1.58 (-1.65--1.52) |
| Canada | 720713.3 (615217.7-844194.4) | 4887.2 (4171.9-5724.6) | 812573.3 (681770.4-963337.2) | 5025 (4216.1-5957.3) | -0.23 (-0.4--0.06) |
| Montenegro | 10229.5 (8273.4-12479.6) | 3211.5 (2597.4-3918) | 9481.3 (7688.4-11460.4) | 3204.3 (2598.3-3873.1) | -0.01 (-0.04-0.02) |
| C么te d'Ivoire | 247330.8 (194057.6-309237.6) | 4443.9 (3486.7-5556.3) | 578770.3 (465871.6-710211) | 4431.4 (3566.9-5437.7) | -0.08 (-0.14--0.01) |
| United Republic of Tanzania | 631749.8 (502538.8-776102.4) | 5588.4 (4445.4-6865.3) | 1425908.5 (1127137.3-1759837.3) | 5347.9 (4227.3-6600.3) | -0.26 (-0.3--0.21) |
| Somalia | 180295.6 (143320.5-221701.9) | 5804.4 (4614-7137.4) | 531897.5 (418777.7-651774.9) | 5619.1 (4424-6885.5) | -0.14 (-0.17--0.11) |
| Croatia | 102348.1 (84708.9-123487) | 4192.9 (3470.3-5058.9) | 63793.5 (52109.9-77420.7) | 3423 (2796.1-4154.2) | -0.91 (-1--0.83) |
| Bahrain | 24792.1 (19851.3-30423.8) | 8202.1 (6567.5-10065.3) | 66740.9 (53607.5-82781.4) | 7221.6 (5800.5-8957.2) | -0.67 (-0.78--0.56) |
| Puerto Rico | 73816.4 (60397.9-87928.1) | 4002.7 (3275.1-4767.9) | 61081.4 (48987.3-74263.5) | 3854.1 (3091-4685.9) | -0.32 (-0.39--0.24) |
| Jordan | 119491.3 (94299.7-148584.6) | 6680.3 (5272-8306.8) | 389633.2 (307016.6-482492.1) | 6149.5 (4845.6-7615.1) | -0.51 (-0.59--0.44) |
| Ghana | 356611.6 (283117.7-439280.6) | 5208.8 (4135.3-6416.3) | 853845.5 (677112.6-1049239.8) | 5140.9 (4076.8-6317.3) | -0.13 (-0.17--0.09) |
| Greece | 366674.6 (292406-449618) | 7261.5 (5790.7-8904.1) | 343344.4 (274602.5-421283.8) | 7679.2 (6141.8-9422.4) | 0.04 (-0.35-0.44) |
| Yemen | 424897 (336064.7-540878.9) | 7795.6 (6165.8-9923.6) | 1212140.5 (949928.9-1500832) | 7749.6 (6073.2-9595.3) | -0.06 (-0.07--0.04) |
| Guatemala | 177019.3 (141381.2-217678.6) | 5101.7 (4074.6-6273.6) | 470309.6 (368264.3-578621.4) | 4914.5 (3848.2-6046.3) | -0.33 (-0.48--0.19) |
| Seychelles | 1097.8 (887.8-1346.8) | 2963.2 (2396.3-3635.5) | 1448.6 (1173.3-1745.3) | 2673.5 (2165.5-3221.1) | -0.45 (-0.52--0.38) |
| Guinea-Bissau | 22956.5 (18167.3-28366.4) | 5189.8 (4107.1-6412.8) | 50356.7 (39398.1-61914.5) | 5321.7 (4163.6-6543.1) | 0.1 (0.08-0.11) |
| Costa Rica | 63559.1 (51502.7-78181.8) | 4125 (3342.5-5074) | 112959.9 (91649-138488.1) | 4526.4 (3672.4-5549.3) | 0.36 (0.33-0.38) |
| Zambia | 164407.5 (130675.4-203490.9) | 4654.7 (3699.7-5761.2) | 420633.4 (333844.8-523847.9) | 4659.4 (3698-5802.7) | -0.1 (-0.15--0.05) |
| United Kingdom | 2022344 (1655901.1-2417609.6) | 7101.3 (5814.6-8489.2) | 1914885.6 (1571876.5-2292565.6) | 6282.1 (5156.8-7521.2) | -0.39 (-0.56--0.22) |
| Denmark | 178340.9 (146465.6-214059.4) | 6673.4 (5480.6-8009.9) | 120676.5 (99371.4-144728) | 4707.6 (3876.5-5645.8) | -1.38 (-1.53--1.22) |
| Switzerland | 242432 (202877.8-288194.7) | 6682.2 (5592-7943.6) | 206034.1 (175033.7-239833.9) | 5158.6 (4382.5-6004.9) | -1.08 (-1.46--0.69) |
| Republic of Moldova | 99633.4 (79922.2-122577.3) | 4531.5 (3635-5575) | 70399.8 (57236.7-86725.8) | 3832.1 (3115.6-4720.7) | -0.7 (-0.79--0.61) |
| France | 1866086.4 (1672525.2-2069027.5) | 6396.8 (5733.2-7092.4) | 1592675.6 (1287272-1929608.2) | 5601.4 (4527.3-6786.4) | -0.48 (-0.59--0.37) |
| Gabon | 38751.1 (30617.5-48721.5) | 8708.7 (6880.8-10949.4) | 76933 (61119.5-95992.6) | 8328.8 (6616.8-10392.2) | -0.22 (-0.27--0.17) |
| Djibouti | 11623.7 (9208.5-14336.5) | 4974.4 (3940.8-6135.4) | 34025.2 (26956-42163.7) | 5298.5 (4197.7-6565.8) | 0.18 (0.16-0.19) |
| Brunei Darussalam | 2837.9 (2283.6-3490.6) | 1940.2 (1561.2-2386.3) | 5115.9 (4111.5-6245.7) | 1922.7 (1545.2-2347.3) | -0.13 (-0.22--0.05) |
| American Samoa | 712.4 (572.6-886.6) | 2924.1 (2350.1-3639.1) | 770.6 (614.4-949.4) | 2806.7 (2237.9-3457.8) | -0.21 (-0.27--0.16) |
| Sri Lanka | 417790.3 (345980.9-503871.5) | 4545.3 (3764.1-5481.9) | 362298.7 (300046.4-431272) | 3322.7 (2751.8-3955.3) | -1.39 (-1.6--1.18) |
| Burundi | 166878.9 (131022.5-206084.9) | 6885.4 (5406-8503.1) | 299995.7 (237538.7-368961.9) | 5473.5 (4334-6731.8) | -1.05 (-1.15--0.95) |
| Iraq | 456179.6 (365625.2-557983) | 5832.7 (4674.8-7134.3) | 1365044 (1091628.7-1658339.8) | 5905.3 (4722.5-7174.1) | -0.04 (-0.48-0.4) |
| Dominican Republic | 190149.4 (149721.3-237300.5) | 5226.6 (4115.3-6522.6) | 304608 (242246.8-370179.3) | 5279.3 (4198.5-6415.8) | -0.04 (-0.09-0.02) |
| Guinea | 133432.6 (105981.5-163657.6) | 5083.4 (4037.6-6234.9) | 294914.8 (236166.9-364001.6) | 5154.1 (4127.4-6361.5) | 0.06 (0.06-0.07) |
| Afghanistan | 374680.6 (296517.6-462384.8) | 7864.4 (6223.8-9705.3) | 1407145 (1123458.9-1736250.5) | 7732 (6173.2-9540.3) | -0.02 (-0.06-0.01) |
| North Macedonia | 30304.5 (24522.4-36641.3) | 2895.4 (2343-3500.9) | 29553.6 (23856.1-35632.6) | 2726.4 (2200.8-3287.2) | -0.34 (-0.4--0.29) |
| Honduras | 76046 (60343.2-93774.5) | 3714.5 (2947.5-4580.5) | 199647.9 (160712.1-247276.7) | 3858.1 (3105.7-4778.5) | 0.18 (0.16-0.2) |
| Bangladesh | 3428047.7 (2763782.1-4181953.7) | 6795 (5478.3-8289.3) | 5527861.2 (4448126.7-6734285.7) | 6415.4 (5162.3-7815.6) | -0.21 (-0.27--0.16) |
| Tokelau | 26.6 (19.8-35.5) | 3835.4 (2853.9-5110.7) | 24.6 (18.6-32) | 3806.3 (2877.6-4963.3) | -0.04 (-0.06--0.02) |
| Lesotho | 59838 (47060.6-74443.5) | 7246.5 (5699.1-9015.2) | 84356.7 (67684.9-103114.4) | 7353.8 (5900.4-8989) | 0.32 (0.22-0.42) |
| Uganda | 643544.9 (506718.7-802335.5) | 8656 (6815.6-10791.8) | 1550348.2 (1233244.2-1920998.6) | 8211.3 (6531.8-10174.4) | -0.69 (-1.09--0.29) |
| Argentina | 600699.9 (485886.7-729702.9) | 3777.6 (3055.6-4588.9) | 877185.3 (752561.6-1009207.3) | 3782.5 (3245.1-4351.7) | -0.04 (-0.1-0.03) |
| Tuvalu | 174.1 (130.3-228) | 3860.4 (2890-5055.7) | 229.5 (171.2-302.7) | 3803.8 (2837.7-5015.8) | -0.06 (-0.08--0.05) |
| Barbados | 5629.4 (4526.8-6968.6) | 4178.3 (3359.9-5172.3) | 6129 (4931.9-7499.4) | 4372.1 (3518.1-5349.7) | 0.19 (0.15-0.23) |
| San Marino | 703.8 (529.8-910.5) | 5861.6 (4412.1-7583.1) | 919.1 (692.4-1210.8) | 6072.1 (4574.6-7999.6) | 0.15 (0.13-0.16) |
| Comoros | 10108.6 (8115.5-12392.3) | 4937.8 (3964.2-6053.3) | 17798.9 (14089.7-21997.3) | 4764.3 (3771.5-5888.2) | -0.22 (-0.28--0.16) |
| Botswana | 29861.1 (23792.6-37260.5) | 4956.2 (3949-6184.3) | 70591.1 (55684-88975.9) | 5336.5 (4209.6-6726.3) | 0.14 (0.06-0.21) |
| Sudan | 647806.3 (511408-807348.6) | 7115.4 (5617.2-8867.8) | 1425624.1 (1138523.8-1764803.5) | 6840.1 (5462.6-8467.4) | -0.2 (-0.23--0.17) |

| **TableS5. DALYs of depression in working-age individuals between 1990 and 2019 in 204 countries** | | | | | |
| --- | --- | --- | --- | --- | --- |
| **Location** | **1990** | | **2019** | |  |
|  | **Number** | **ASDR** | **Number** | **ASDR** | **EAPC** |
| Mexico | 1648247.9 (1374588.7-1939203.6) | 3877.2 (3233.5-4561.6) | 3520979.3 (2948672.5-4151559.5) | 5311.7 (4448.4-6263) | 1.42 (1.26-1.58) |
| Haiti | 161449.3 (126981.4-201465.8) | 5510.2 (4333.9-6876) | 344782.5 (272057-430272.3) | 5261.6 (4151.8-6566.2) | -0.18 (-0.2--0.15) |
| Viet Nam | 846730 (683785.5-1043545.9) | 2580.8 (2084.1-3180.7) | 1202194 (977007.3-1448868.7) | 2267.3 (1842.6-2732.6) | -0.48 (-0.51--0.44) |
| Bhutan | 14944.3 (12075.7-18399.5) | 4930 (3983.7-6069.9) | 21450.1 (16970.3-26668) | 4923.9 (3895.6-6121.7) | -0.21 (-0.3--0.11) |
| Jamaica | 45905.8 (35816.8-58112.9) | 3943.9 (3077.1-4992.6) | 62907.3 (49454.5-77770.1) | 4108.7 (3230.1-5079.5) | 0.08 (0-0.16) |
| Nicaragua | 78979.4 (62470.9-97696.3) | 4581.3 (3623.7-5667) | 160936.9 (128993.8-196878.5) | 4592.5 (3681-5618.2) | -0.08 (-0.17-0.02) |
| Kyrgyzstan | 85506.7 (68142-106048.1) | 4080.7 (3252-5061.1) | 131572.3 (106957.3-160325.1) | 3917.2 (3184.3-4773.2) | -0.17 (-0.19--0.14) |
| Georgia | 93073.9 (75142.5-114742.4) | 3470.1 (2801.6-4278) | 60626.8 (49102.1-73777.5) | 3665.3 (2968.6-4460.4) | 0.2 (0.17-0.24) |
| Lebanon | 99990.8 (79988.1-122018.9) | 6635.3 (5308-8097.1) | 192545.1 (152451.9-237351.8) | 7205.6 (5705.2-8882.5) | 0.31 (0.14-0.47) |
| Kazakhstan | 330368.4 (269374.5-401738.7) | 4017.9 (3276.1-4885.9) | 394692.5 (324398.6-479902.2) | 4252 (3494.7-5169.9) | 0.37 (0.28-0.46) |
| Namibia | 27466.5 (21971.8-34052.3) | 4173.1 (3338.3-5173.7) | 52164.3 (41855.8-64274.2) | 4155.8 (3334.5-5120.6) | -0.19 (-0.32--0.06) |
| Republic of Korea | 685368.6 (578191.4-806114.3) | 2648.2 (2234-3114.7) | 723887.9 (619465.7-837362.8) | 2779.2 (2378.3-3214.9) | 0.27 (0.12-0.42) |
| Timor-Leste | 14017.8 (11273.3-17108.2) | 3663.4 (2946.2-4471.1) | 19463.2 (15450.1-24122.5) | 2982 (2367.1-3695.8) | -0.87 (-0.98--0.76) |
| China | 23206347.5 (19507637.1-27341422.8) | 3471.6 (2918.3-4090.2) | 17943053.6 (15225607.2-20896343) | 2489.6 (2112.5-2899.3) | -1.57 (-1.77--1.37) |
| Eritrea | 80247.7 (63926.4-99379.8) | 5886.8 (4689.5-7290.3) | 194633.6 (153271.8-239203.4) | 5634.3 (4436.9-6924.5) | -0.17 (-0.2--0.13) |
| Iceland | 5800.2 (4796.3-6998.1) | 4379.3 (3621.3-5283.8) | 6103.9 (5014.7-7383) | 3739.7 (3072.4-4523.5) | -0.69 (-0.78--0.61) |
| Panama | 47199.7 (37630.6-58041.7) | 3834.3 (3057-4715.1) | 81919.2 (65666-99574.7) | 3897.7 (3124.4-4737.8) | 0.06 (0.03-0.1) |
| Serbia | 166801.9 (137206.6-200844.4) | 3631.3 (2987-4372.4) | 125228.2 (101713.6-151657.1) | 3128.7 (2541.2-3789) | -0.65 (-0.71--0.59) |
| India | 22274565.2 (18539395.9-26486767.3) | 5302.7 (4413.5-6305.5) | 33277196.5 (27928639.3-39352621.6) | 4380.9 (3676.8-5180.8) | -1.52 (-1.92--1.13) |
| Libya | 122333.3 (96957.4-152320.3) | 6322 (5010.6-7871.7) | 281612.2 (225034.6-345039.9) | 6855.2 (5477.9-8399.2) | 0.26 (0.25-0.28) |
| South Africa | 1054804.3 (886551-1250511.9) | 5539.4 (4655.8-6567.2) | 1724868.3 (1451632.9-2032643.6) | 5664.3 (4767-6675) | 0.26 (0.09-0.43) |
| Democratic People's Republic of Korea | 297356.1 (239218.1-358660) | 2786.9 (2242-3361.4) | 338918.4 (276901.5-416312.8) | 2435.6 (1989.9-2991.7) | -0.56 (-0.61--0.52) |
| Uruguay | 56239.4 (46188.1-68030) | 3785.1 (3108.7-4578.7) | 72810.7 (59629.8-88284.8) | 4417.6 (3617.9-5356.5) | 0.84 (0.73-0.94) |
| Japan | 1935676.9 (1666823.6-2231041.3) | 2979.8 (2565.9-3434.5) | 1675454.5 (1439994-1945709.2) | 3237.2 (2782.3-3759.4) | 0.56 (0.35-0.77) |
| Poland | 472227.3 (387345-572232) | 2488.3 (2041-3015.3) | 456409.9 (375685-551741.9) | 2507.8 (2064.2-3031.6) | -0.55 (-0.72--0.38) |
| Saint Vincent and the Grenadines | 2327.1 (1838.6-2858.9) | 4362.6 (3446.9-5359.6) | 2742.5 (2207.2-3337.2) | 4795.9 (3859.8-5835.9) | 0.37 (0.33-0.42) |
| Australia | 656521.7 (556442.4-767712.4) | 7305.5 (6191.9-8542.8) | 872971.6 (716992.9-1061979.9) | 7565.7 (6213.9-9203.8) | 0.14 (-0.03-0.32) |
| Cook Islands | 363.5 (267.8-479.4) | 3840.2 (2828.9-5064.4) | 315.6 (236.5-410.4) | 3865.4 (2896.4-5026.6) | -0.01 (-0.03-0.01) |
| Liberia | 47381.2 (37370.9-58924.6) | 5797.9 (4573-7210.5) | 137311.7 (109354.2-168127.2) | 5551.5 (4421.2-6797.4) | 0.52 (0.31-0.74) |
| Greenland | 4327.9 (3532.2-5322.1) | 12939.1 (10560.5-15911.5) | 2986.5 (2411.9-3624.4) | 11234.1 (9072.7-13633.6) | -0.48 (-0.52--0.44) |
| Tajikistan | 75785.9 (60359.6-93799.2) | 3142.5 (2502.9-3889.5) | 145401.8 (116418.2-179558.8) | 2926.2 (2342.9-3613.6) | -0.33 (-0.39--0.26) |
| Fiji | 13253.9 (10714.2-16207.3) | 3352.5 (2710.1-4099.5) | 15591.7 (12578.5-19132.5) | 3344.7 (2698.3-4104.2) | -0.07 (-0.1--0.05) |
| Bermuda | 1751.8 (1409.2-2143.6) | 5176.1 (4163.9-6333.8) | 1214.1 (972.4-1488.9) | 4376.7 (3505.6-5367.4) | -0.69 (-0.74--0.63) |
| Israel | 148329.2 (120889.7-180571.3) | 6109.2 (4979.1-7437.2) | 252203.5 (204015.1-302118.2) | 5762.7 (4661.6-6903.2) | -0.56 (-0.79--0.34) |
| United States Virgin Islands | 2485.5 (1991.8-3053) | 4529.2 (3629.6-5563.4) | 2054 (1636.2-2522.1) | 4729.2 (3767.1-5806.7) | 0.18 (0.15-0.22) |
| Pakistan | 2434784.3 (1954415.4-2973928.2) | 4908.2 (3939.9-5995.1) | 5394557.8 (4289510.4-6589997.5) | 4826.3 (3837.7-5895.9) | -0.13 (-0.19--0.06) |
| Guam | 3163.9 (2556.4-3879.7) | 4093.8 (3307.8-5019.9) | 3317.9 (2710.1-4032) | 4090.6 (3341.2-4971) | 0.05 (-0.03-0.13) |
| Mauritania | 37968.8 (30277.9-46309.8) | 4163.1 (3319.8-5077.6) | 75840.8 (60230.6-93770.4) | 3914.9 (3109.1-4840.5) | -0.31 (-0.4--0.22) |
| Cambodia | 181623.4 (143487.2-226697.6) | 3953.9 (3123.7-4935.2) | 284295.6 (231247.7-350160.9) | 3235.4 (2631.7-3985) | -0.86 (-0.92--0.79) |
| Singapore | 83810.6 (72097.7-98028.9) | 4407.6 (3791.7-5155.4) | 86032.3 (71927.8-101856.1) | 2797.1 (2338.5-3311.5) | -1.97 (-2.19--1.75) |
| Nepal | 521660.6 (422004.8-633101.1) | 5864 (4743.8-7116.7) | 1001479.6 (813559-1206443.1) | 6188.5 (5027.3-7455.1) | 0.33 (0.19-0.47) |
| South Sudan | 147732.1 (116662.9-182626.9) | 5402 (4265.9-6678) | 236174.5 (188867.7-290014.8) | 5601.3 (4479.4-6878.2) | 0.11 (0.09-0.13) |
| Slovakia | 88698.5 (72658.9-107814.2) | 3319.4 (2719.2-4034.8) | 78257.6 (62561.2-94429.9) | 2974.2 (2377.7-3588.8) | -0.56 (-0.73--0.39) |
| Mongolia | 53909.1 (42954.6-67232.3) | 5254.2 (4186.5-6552.8) | 95382.7 (76952.1-116262.6) | 5276.6 (4257-6431.6) | 0 (-0.03-0.03) |
| Italy | 1478109.3 (1204067.6-1782867.7) | 5146.1 (4192-6207.1) | 1270912.5 (1039757.2-1522854.3) | 5011.6 (4100.1-6005.1) | -0.66 (-0.86--0.46) |
| Kiribati | 1433.4 (1130.4-1811.6) | 3909 (3082.6-4940.1) | 2094.8 (1684.5-2597.2) | 3466.7 (2787.8-4298.1) | -0.43 (-0.49--0.37) |
| Ireland | 106231 (89350.1-127001.6) | 5962.9 (5015.4-7128.8) | 148689.1 (123407.1-177329.9) | 6376.9 (5292.6-7605.2) | 0.05 (-0.05-0.14) |
| Andorra | 1670.1 (1357.4-2054) | 5152.5 (4187.7-6336.6) | 2118.9 (1700.3-2627.1) | 5173.3 (4151.4-6414) | -0.02 (-0.06-0.01) |
| Kenya | 585894.4 (485626.7-694289.3) | 5772.5 (4784.6-6840.5) | 1431559.6 (1194491-1687576.3) | 5495.9 (4585.8-6478.8) | -0.26 (-0.36--0.17) |
| Mali | 145812.2 (116944.1-178015) | 4035 (3236.1-4926.1) | 366525 (290336.9-453603.5) | 3795.7 (3006.7-4697.5) | -0.32 (-0.43--0.2) |
| Morocco | 990057.4 (783766.6-1223312) | 7952.5 (6295.5-9826.1) | 1516919.8 (1214171-1878873) | 7913.1 (6333.8-9801.2) | -0.04 (-0.06--0.03) |
| Romania | 337352.3 (273406.4-405009.6) | 2974.4 (2410.6-3570.9) | 255215.9 (207693.2-310217.3) | 2947.6 (2398.7-3582.8) | -0.04 (-0.08--0.01) |
| Zimbabwe | 147588.7 (116656.2-182780.4) | 3205.6 (2533.8-3970) | 252946.1 (201593-312115.1) | 3362.7 (2680-4149.3) | 0.15 (0.05-0.25) |
| Eswatini | 17517.5 (13820.6-21816) | 4920.4 (3882-6127.8) | 33497.6 (27000.8-41185.6) | 5530.7 (4458.1-6800.1) | 0.43 (0.38-0.48) |
| United States of America | 7236205.9 (6213595.3-8490644.6) | 5406 (4642-6343.2) | 10867823.4 (9331179.9-12667233.1) | 7218.5 (6197.8-8413.7) | 0.69 (0.33-1.05) |
| Turkmenistan | 62843.3 (50560.5-77804.9) | 3569.7 (2872-4419.5) | 87677.2 (70881-107139.1) | 3327.3 (2689.9-4065.9) | -0.28 (-0.33--0.23) |
| Venezuela (Bolivarian Republic of) | 396129.6 (316351.6-491569.6) | 4128.1 (3296.7-5122.7) | 638513.4 (512009.6-775488.1) | 4436.2 (3557.3-5387.9) | 0.17 (0.13-0.2) |
| Marshall Islands | 717.4 (566.5-899.3) | 3564 (2814.3-4467.7) | 1007.4 (802.5-1244.8) | 3326.9 (2650.3-4110.8) | -0.27 (-0.3--0.25) |
| Trinidad and Tobago | 38412.7 (31135.8-48367.9) | 6205.6 (5030-7813.9) | 40604 (32800-49082.7) | 5845.6 (4722.1-7066.3) | -0.27 (-0.32--0.22) |
| Taiwan (Province of China) | 237798.7 (194245.3-285402.3) | 2112.7 (1725.8-2535.6) | 284584.8 (235592.8-342472.2) | 2435.3 (2016.1-2930.7) | 0.57 (0.51-0.64) |
| Angola | 434859.8 (342011.2-544221.7) | 9299.2 (7313.7-11637.8) | 1177137.5 (911676.2-1482837.4) | 8700.7 (6738.6-10960.3) | -0.29 (-0.32--0.26) |
| Palestine | 87297.9 (69432.8-109011.8) | 9885.4 (7862.4-12344.2) | 251431.7 (199174.1-315641.6) | 9912.6 (7852.3-12444) | 0.15 (-0.12-0.43) |
| Suriname | 13688.6 (11139.3-16890.1) | 6962.1 (5665.5-8590.4) | 21562.1 (17435.8-26251.4) | 7481.4 (6049.7-9108.4) | 0.35 (0.28-0.42) |
| Saint Lucia | 2959.7 (2351-3627.8) | 4448 (3533.2-5452.1) | 4398.9 (3541.8-5384.4) | 4699 (3783.4-5751.7) | 0.19 (0.15-0.23) |
| Niger | 164356.7 (131255.6-203358.5) | 4913.6 (3924-6079.6) | 453350.6 (362955.6-555208.4) | 4784.4 (3830.4-5859.3) | -0.11 (-0.13--0.09) |
| Bahamas | 5821.7 (4646.9-7169.1) | 4087.7 (3262.8-5033.8) | 8476.3 (6721.6-10423.5) | 4140.5 (3283.3-5091.6) | -0.02 (-0.08-0.04) |
| Ethiopia | 1382137.3 (1118927.9-1686353.9) | 6247.7 (5057.9-7622.8) | 2807674.8 (2261317.7-3422371.9) | 5347.7 (4307.1-6518.5) | -0.71 (-0.86--0.56) |
| Micronesia (Federated States of) | 1722.2 (1375.7-2148.4) | 3639.6 (2907.3-4540.3) | 1785 (1444.1-2208.7) | 3332.7 (2696.2-4123.6) | -0.38 (-0.41--0.34) |
| Lao People's Democratic Republic | 64072.7 (51293.1-79312.7) | 3447 (2759.5-4266.9) | 117197.7 (95527.1-143444) | 3007.9 (2451.7-3681.5) | -0.6 (-0.73--0.46) |
| Belarus | 254993.4 (205474.1-310142.8) | 5047.8 (4067.5-6139.5) | 225550.3 (180875.2-275101.4) | 5176.5 (4151.2-6313.8) | 0.03 (-0.09-0.15) |
| Malta | 7859.5 (6333-9605.9) | 4104 (3306.9-5015.9) | 7979.3 (6422.2-9615.1) | 4050.3 (3259.9-4880.7) | -0.19 (-0.28--0.11) |
| Samoa | 2793 (2203.2-3499.9) | 3575.5 (2820.4-4480.3) | 3271.4 (2649.4-4052.9) | 3146.3 (2548.1-3897.9) | -0.56 (-0.62--0.5) |
| Brazil | 4634712.3 (3887561.4-5504629.1) | 6046.9 (5072.1-7181.9) | 6287672.6 (5413601.8-7242406.4) | 5444.4 (4687.6-6271.1) | -0.57 (-0.96--0.17) |
| Dominica | 1394.4 (1105.9-1712.9) | 3950.6 (3133.2-4853.2) | 1424.8 (1133.4-1757.5) | 4194.9 (3336.9-5174.7) | 0.22 (0.17-0.27) |
| Latvia | 74412.8 (60379.3-92638) | 5788.6 (4697-7206.4) | 40810.4 (33936.4-48714.7) | 4981.9 (4142.7-5946.8) | -0.68 (-0.76--0.6) |
| Uzbekistan | 360906.9 (290754.6-445587.5) | 3688.3 (2971.4-4553.7) | 648497.7 (526559.3-797485.3) | 3586.8 (2912.4-4410.9) | -0.12 (-0.15--0.09) |
| Philippines | 1103767.3 (890610-1341112.5) | 3542.1 (2858.1-4303.8) | 1714352.7 (1392123.2-2082902.3) | 2932.5 (2381.3-3563) | -0.72 (-0.96--0.47) |
| Luxembourg | 11128.5 (9200.7-13552.7) | 5593.8 (4624.8-6812.4) | 13204.9 (11216.9-15469) | 4303.1 (3655.3-5040.9) | -1.09 (-1.17--1.02) |
| Mauritius | 35377.6 (28809.2-44146.3) | 5817.4 (4737.3-7259.3) | 32523.6 (26053.8-39536.9) | 5003.1 (4007.8-6081.9) | -0.62 (-0.74--0.5) |
| Paraguay | 94119 (74694.6-117580.2) | 4980.2 (3952.4-6221.6) | 201066.1 (160721.4-245978.4) | 5407.8 (4322.7-6615.7) | 0.3 (0.19-0.41) |
| Benin | 103240.5 (82178.9-127516.8) | 5156.1 (4104.2-6368.5) | 302170.7 (241877.6-370998.2) | 5188.5 (4153.2-6370.3) | 0.01 (0-0.03) |
| Malaysia | 324655.6 (258886.1-396737.3) | 3605.3 (2875-4405.8) | 770633.6 (637034.3-922577.6) | 4414.6 (3649.3-5285) | 0.99 (0.76-1.23) |
| Ecuador | 226154.1 (183078.3-280733.3) | 4568.1 (3698-5670.5) | 413340.8 (331156.8-505773.6) | 4496.4 (3602.4-5501.9) | -0.08 (-0.12--0.05) |
| Monaco | 831.6 (630.7-1095) | 6038.5 (4579.9-7951.4) | 857.1 (648.3-1113.4) | 6013.2 (4548.7-7811.8) | 0.01 (-0.01-0.02) |
| Qatar | 19502.9 (15648-24200) | 6679.4 (5359.1-8288) | 132376.5 (104526.3-165900.5) | 6130.6 (4840.8-7683.1) | -0.55 (-0.67--0.42) |
| El Salvador | 143725.6 (116454-179048.4) | 5752.9 (4661.3-7166.8) | 164493 (131868.3-203324.8) | 5013.7 (4019.3-6197.2) | -0.58 (-0.63--0.53) |
| Armenia | 48862 (39129-59900) | 2861.1 (2291.2-3507.4) | 47312.5 (38063.7-58317.4) | 3201.8 (2575.9-3946.6) | 0.52 (0.47-0.57) |
| Iran (Islamic Republic of) | 2025126.6 (1581705.1-2528988.2) | 7660.1 (5982.8-9565.9) | 3892758.1 (3092144.3-4828898.8) | 8234.5 (6540.9-10214.7) | 0.65 (0.46-0.83) |
| Cuba | 471246 (384523.2-578113.2) | 7673.9 (6261.7-9414.2) | 280241.4 (229076.7-338119.4) | 5280.3 (4316.3-6370.9) | -1.53 (-1.68--1.38) |
| Nigeria | 2076702.3 (1669979.3-2530752.4) | 5067.9 (4075.4-6176) | 4088184.8 (3316722-4960889.4) | 4052.6 (3287.8-4917.7) | -0.91 (-1.43--0.39) |
| Myanmar | 358605.8 (289489.1-436495.8) | 1777.9 (1435.2-2164.1) | 510152.9 (410796.3-620963.4) | 1764.6 (1420.9-2147.9) | -0.11 (-0.25-0.04) |
| Malawi | 205065.8 (164167.2-251495.5) | 4802.7 (3844.9-5890.1) | 398520.8 (319904.1-490384.1) | 4428.3 (3554.7-5449) | -0.42 (-0.5--0.34) |
| Oman | 55196 (43878.2-68553.9) | 5753.1 (4573.4-7145.4) | 180904.7 (141730.5-226805.5) | 5780.8 (4529-7247.5) | -0.05 (-0.08--0.02) |
| Congo | 100540.9 (78986.4-127179.9) | 9086.2 (7138.3-11493.7) | 224661.8 (180004.7-279156.6) | 8395.9 (6727-10432.4) | -0.35 (-0.45--0.24) |
| Madagascar | 302390 (239038.7-378372.9) | 5706.4 (4510.9-7140.3) | 727396.5 (576179.6-890956.2) | 5508.2 (4363.1-6746.8) | -0.16 (-0.18--0.14) |
| Papua New Guinea | 82092.6 (65416-102735.3) | 4130.7 (3291.5-5169.3) | 194870.8 (156258.3-243180.3) | 3832.5 (3073.1-4782.6) | -0.3 (-0.32--0.29) |
| Indonesia | 2191392.9 (1780202.1-2654999.1) | 2317.8 (1882.9-2808.2) | 3309970.5 (2714693.4-3987976.5) | 2318 (1901.1-2792.8) | -0.08 (-0.14--0.02) |
| New Zealand | 106994.6 (84779.5-131678.6) | 5928.2 (4697.3-7295.8) | 122933.2 (101859.2-146160.7) | 6192.6 (5131.1-7362.7) | 0.1 (0.06-0.14) |
| Bolivia (Plurinational State of) | 148931.2 (118717.8-183531.4) | 4988.1 (3976.2-6147) | 270788.2 (217099.1-332122.8) | 4433.1 (3554.1-5437.2) | -0.48 (-0.52--0.43) |
| Sao Tome and Principe | 2123.4 (1650.8-2650.5) | 4244.5 (3299.7-5297.9) | 4640 (3653-5779.5) | 4334.8 (3412.7-5399.3) | 0.04 (0.01-0.08) |
| Antigua and Barbuda | 1237.4 (955.3-1552) | 3937.7 (3040.1-4938.8) | 1947.8 (1521.7-2408.9) | 4078.7 (3186.5-5044.4) | 0.03 (-0.05-0.11) |
| Belgium | 224155.5 (199955.8-251038.1) | 4520.3 (4032.3-5062.5) | 265025.7 (216964.1-317584.8) | 5290.3 (4331-6339.5) | 0.85 (0.69-1.02) |
| Nauru | 189.2 (140.2-252.1) | 3808.2 (2821.6-5075.2) | 214.6 (159.9-283.5) | 3817.5 (2844.8-5043.4) | 0 (-0.01-0.02) |
| Burkina Faso | 199573.6 (159591.8-243388.8) | 5194.5 (4153.8-6334.9) | 511732.5 (407715.8-624485.1) | 4997.4 (3981.6-6098.5) | 0.35 (0.21-0.5) |
| Bosnia and Herzegovina | 100976.1 (82723.5-122344.6) | 4158.7 (3407-5038.8) | 46850 (37379.9-57593.4) | 3086.3 (2462.4-3794) | -1.42 (-1.56--1.28) |
| Bulgaria | 140595 (116262-169638.2) | 3381.9 (2796.6-4080.5) | 87968.2 (72008-108235.8) | 2903.1 (2376.4-3571.9) | -0.77 (-0.85--0.68) |
| Democratic Republic of the Congo | 1414677.2 (1118698.6-1783168.4) | 8444.1 (6677.4-10643.6) | 3362854.2 (2667181.1-4236857.1) | 8058.7 (6391.6-10153.2) | -0.21 (-0.24--0.18) |
| Norway | 87400.7 (71716.3-105086.7) | 4034.2 (3310.3-4850.6) | 117204.8 (95445.1-141286.9) | 4705 (3831.5-5671.7) | 0.36 (0.19-0.52) |
| Algeria | 730522.6 (573283.7-912887.5) | 6200.6 (4866-7748.5) | 1382862.3 (1090100.1-1711710.4) | 6166.6 (4861.1-7633.1) | -0.09 (-0.14--0.04) |
| Slovenia | 49567.6 (40777.5-60310.6) | 4887.9 (4021.1-5947.2) | 32859.4 (26653.5-39547.4) | 3666.9 (2974.4-4413.3) | -1.25 (-1.35--1.15) |
| Portugal | 362266.4 (295913-443268.4) | 7245.2 (5918.2-8865.2) | 297457 (238610.3-365547) | 6341.1 (5086.6-7792.6) | -0.57 (-0.75--0.39) |
| Chile | 509259.5 (449853.5-578230.3) | 7166.8 (6330.8-8137.5) | 557070.9 (454173.3-680929.2) | 6057.7 (4938.8-7404.5) | -0.68 (-0.76--0.6) |
| Solomon Islands | 5865.4 (4642.9-7323.3) | 3868.2 (3062-4829.7) | 11787.2 (9381.5-14609.2) | 3590.4 (2857.6-4450) | -0.33 (-0.35--0.3) |
| Cabo Verde | 8103.6 (6453.6-9938.8) | 5506.6 (4385.4-6753.7) | 19111.5 (15383-23528.7) | 6209.7 (4998.2-7644.9) | 0.4 (0.28-0.51) |
| Czechia | 208703.2 (174280.6-249931.3) | 4002.4 (3342.3-4793.1) | 164312.6 (133069.8-199142.7) | 3421.4 (2770.9-4146.7) | -0.74 (-0.79--0.68) |
| Netherlands | 428444.3 (382946.2-479242.2) | 5285.6 (4724.3-5912.3) | 380203.1 (310232.7-457692.6) | 5065.4 (4133.2-6097.8) | -0.24 (-0.33--0.16) |
| Senegal | 144758 (116041.2-177284.9) | 4435.2 (3555.4-5431.8) | 324757.1 (255783.9-396025.3) | 4423.2 (3483.8-5393.9) | -0.07 (-0.16-0.03) |
| Northern Mariana Islands | 858.2 (698.2-1057.4) | 2955 (2403.8-3640.5) | 637.5 (516.8-774.4) | 3112.8 (2523.4-3781) | 0.19 (0.13-0.25) |
| Tunisia | 304840.6 (241882.4-378928.2) | 7430 (5895.5-9235.8) | 455662 (361952.7-568087) | 7544.7 (5993.1-9406.2) | -0.01 (-0.05-0.04) |
| Hungary | 224409.4 (186058.3-272200.5) | 4407.9 (3654.6-5346.6) | 142219.6 (116278-171046.1) | 3203.3 (2619-3852.5) | -1.28 (-1.38--1.18) |
| Sierra Leone | 81408.1 (64397.6-100800.4) | 4953.2 (3918.2-6133.1) | 215545.8 (171148.6-266318.1) | 5188.3 (4119.6-6410.4) | 0.14 (0.08-0.2) |
| Guyana | 29172 (23590.8-36335.9) | 7243.7 (5857.9-9022.6) | 33308 (26796.9-40841.1) | 8034.2 (6463.6-9851.2) | 0.48 (0.35-0.62) |
| Central African Republic | 117458.4 (90898.5-147096.2) | 9393.2 (7269.2-11763.4) | 234558.6 (184815.9-290412.9) | 9155.7 (7214-11335.9) | -0.14 (-0.16--0.11) |
| Germany | 1663524.3 (1445037.7-1933289.9) | 4171 (3623.2-4847.4) | 1702358 (1397670.6-2039825.4) | 4742.7 (3893.8-5682.8) | 0.93 (0.7-1.17) |
| Kuwait | 62157.2 (49487.1-77438.4) | 5899.9 (4697.3-7350.4) | 183153.8 (145436.7-228030.1) | 6291.2 (4995.7-7832.7) | 0.15 (0.12-0.18) |
| Mozambique | 322524.9 (257411.1-394373.9) | 5758.2 (4595.7-7040.9) | 780269.1 (617524.1-957369.8) | 5856.3 (4634.8-7185.5) | 0.05 (0.03-0.07) |
| Grenada | 1687.6 (1351.6-2086.4) | 4316.9 (3457.3-5337) | 2431.9 (1964.1-2951) | 4489.8 (3626.1-5448.1) | 0.14 (0.09-0.19) |
| Saudi Arabia | 472514.3 (374308.8-585091.3) | 5844.2 (4629.6-7236.6) | 1549107.7 (1233924.6-1905781.8) | 6359.7 (5065.8-7824) | 0.32 (0.3-0.35) |
| Colombia | 512242 (409986.7-631169.3) | 3025.6 (2421.6-3728.1) | 669601.9 (560028-788162.1) | 2689 (2249-3165.1) | -0.75 (-1.07--0.42) |
| Russian Federation | 3021304.8 (2455057.9-3695686.1) | 4068.9 (3306.3-4977.1) | 2776064.6 (2262726.1-3371956.4) | 4054.3 (3304.6-4924.6) | -0.19 (-0.33--0.06) |
| Cameroon | 242881.1 (192164.7-301219.4) | 5365.5 (4245.1-6654.2) | 785725.7 (625434.8-964537.8) | 5465.1 (4350.2-6708.8) | 0.05 (0.02-0.08) |
| Syrian Arab Republic | 338321.9 (264978.8-421619.1) | 6017.7 (4713.1-7499.3) | 456935.1 (358980.2-565017.6) | 6153.3 (4834.2-7608.8) | 0 (-0.09-0.08) |
| Lithuania | 106362.4 (87369.1-127918.2) | 5810.2 (4772.7-6987.7) | 66105.6 (54012.6-80892.3) | 5456.6 (4458.4-6677.1) | -0.37 (-0.48--0.25) |
| Albania | 42682.6 (34370.4-52604.4) | 2497.4 (2011-3077.9) | 34413.5 (28049.8-41765.7) | 2660.8 (2168.8-3229.3) | 0.2 (0.12-0.27) |
| Chad | 155168.8 (123121.2-193594.8) | 6228.9 (4942.4-7771.5) | 430990.7 (343306.9-532429.3) | 6303.1 (5020.7-7786.5) | 0.05 (-0.01-0.12) |
| Austria | 210259.1 (171621.8-251095.6) | 5224.5 (4264.4-6239.2) | 166261.1 (137236.9-200837.7) | 4120 (3400.8-4976.8) | -0.99 (-1.1--0.87) |
| Rwanda | 221979.1 (172588.3-276276.2) | 7062.9 (5491.4-8790.5) | 389143.1 (307474.2-478965) | 6015.9 (4753.4-7404.5) | -1.01 (-1.17--0.85) |
| Belize | 3683.9 (2951.2-4531.3) | 4324.4 (3464.3-5319.1) | 10326 (8302.6-12668.3) | 4601 (3699.5-5644.7) | 0.22 (0.15-0.29) |
| Finland | 206454.5 (172723.6-250724.1) | 7995.7 (6689.3-9710.2) | 141795 (117780.4-169858.8) | 6073.8 (5045.2-7275.9) | -1.17 (-1.33--1.01) |
| Egypt | 1430563.9 (1128075.1-1780139.9) | 5330.3 (4203.2-6632.8) | 2853629.6 (2250711.4-3570502.4) | 5548.8 (4376.5-6942.8) | 0.07 (-0.05-0.19) |
| Vanuatu | 2725.9 (2181.9-3408.2) | 3885.3 (3109.9-4857.8) | 5184.4 (4138.2-6404) | 3567.6 (2847.7-4406.9) | -0.36 (-0.38--0.34) |
| Thailand | 978867.4 (785785.3-1196496.4) | 3095.5 (2484.9-3783.7) | 1065778.3 (867249.7-1289608.4) | 2986.8 (2430.4-3614) | -0.15 (-0.22--0.08) |
| Togo | 82678 (64890.6-102247) | 5142.1 (4035.8-6359.2) | 209277.9 (167095.3-257065.1) | 5325.3 (4252-6541.4) | 0.12 (0.09-0.14) |
| Spain | 1039290.9 (918595.8-1169547.3) | 5351 (4729.6-6021.7) | 1406610.7 (1216407.9-1611910.7) | 6807.5 (5887-7801.1) | 1.18 (0.84-1.53) |
| Peru | 300404.3 (240637.9-370848.9) | 2811.1 (2251.8-3470.3) | 438482.7 (355996.2-531518) | 2459.8 (1997.1-2981.7) | -0.65 (-0.74--0.55) |
| Niue | 39.1 (29.5-51.3) | 3798.4 (2867.4-4989) | 29.3 (22.2-38.6) | 3804.1 (2884.9-5013.8) | -0.01 (-0.02-0.01) |
| Turkey | 1810518 (1570754.6-2075782.2) | 6046.7 (5246-6932.7) | 2655120.4 (2149768.2-3262523.9) | 5814.9 (4708.2-7145.2) | -0.06 (-0.22-0.11) |
| Tonga | 1312.5 (1049.6-1629.8) | 2966.3 (2372.1-3683.3) | 1410.6 (1137.9-1734.5) | 2885.7 (2327.8-3548.4) | -0.17 (-0.21--0.14) |
| Gambia | 31445.1 (24663.8-39385.7) | 7086.2 (5558-8875.6) | 75913.5 (60087.5-95100.4) | 6812.1 (5392-8533.8) | -0.34 (-0.43--0.25) |
| Sweden | 273008.5 (231382.9-320609.5) | 6497.4 (5506.7-7630.2) | 278033.3 (237517.3-326410.9) | 6209.3 (5304.5-7289.8) | -0.29 (-0.35--0.22) |
| Ukraine | 1405662.7 (1140187.7-1699638.6) | 5635.9 (4571.5-6814.6) | 1104741.7 (899804.4-1343040) | 5315.4 (4329.4-6462) | -0.54 (-0.71--0.37) |
| Estonia | 48530.6 (39321.6-60224.5) | 6391 (5178.3-7931) | 26484.2 (21341.2-32894.5) | 4552.5 (3668.4-5654.4) | -1.6 (-1.77--1.44) |
| Cyprus | 15989.6 (12800.6-19646.7) | 3962.8 (3172.5-4869.2) | 27652.9 (21885.8-33744.5) | 4010 (3173.7-4893.4) | -0.06 (-0.14-0.01) |
| Saint Kitts and Nevis | 1076.5 (794.6-1428) | 5412.2 (3995-7179.4) | 1788.2 (1359.6-2362.6) | 5689.2 (4325.4-7516.5) | 0.16 (0.13-0.19) |
| Palau | 327.8 (244.2-431.6) | 3816.4 (2843.7-5024.8) | 346.9 (263.3-452.3) | 3739.8 (2838.4-4876.3) | -0.08 (-0.11--0.06) |
| Azerbaijan | 100553.4 (79357.1-123784.4) | 2737.6 (2160.6-3370.1) | 158239.1 (125901.8-192858.2) | 2844 (2262.8-3466.2) | 0.17 (0.11-0.23) |
| United Arab Emirates | 68679.6 (54991.5-84359.1) | 5814.9 (4655.9-7142.4) | 384345 (298882.1-488462) | 5517.3 (4290.5-7012) | -0.43 (-0.56--0.3) |
| Equatorial Guinea | 17059.4 (13356.8-21308.8) | 9505.1 (7442.1-11872.8) | 63628.8 (50301.9-80244.2) | 8502.2 (6721.4-10722.3) | -0.45 (-0.51--0.39) |
| Maldives | 4437.9 (3548-5483.8) | 4659.5 (3725.2-5757.6) | 9977.6 (8169.5-12165) | 3132.4 (2564.7-3819.1) | -1.58 (-1.65--1.52) |
| Canada | 720713.3 (615217.7-844194.4) | 4887.2 (4171.9-5724.6) | 812573.3 (681770.4-963337.2) | 5025 (4216.1-5957.3) | -0.23 (-0.4--0.06) |
| Montenegro | 10229.5 (8273.4-12479.6) | 3211.5 (2597.4-3918) | 9481.3 (7688.4-11460.4) | 3204.3 (2598.3-3873.1) | -0.01 (-0.04-0.02) |
| C么te d'Ivoire | 247330.8 (194057.6-309237.6) | 4443.9 (3486.7-5556.3) | 578770.3 (465871.6-710211) | 4431.4 (3566.9-5437.7) | -0.08 (-0.14--0.01) |
| United Republic of Tanzania | 631749.8 (502538.8-776102.4) | 5588.4 (4445.4-6865.3) | 1425908.5 (1127137.3-1759837.3) | 5347.9 (4227.3-6600.3) | -0.26 (-0.3--0.21) |
| Somalia | 180295.6 (143320.5-221701.9) | 5804.4 (4614-7137.4) | 531897.5 (418777.7-651774.9) | 5619.1 (4424-6885.5) | -0.14 (-0.17--0.11) |
| Croatia | 102348.1 (84708.9-123487) | 4192.9 (3470.3-5058.9) | 63793.5 (52109.9-77420.7) | 3423 (2796.1-4154.2) | -0.91 (-1--0.83) |
| Bahrain | 24792.1 (19851.3-30423.8) | 8202.1 (6567.5-10065.3) | 66740.9 (53607.5-82781.4) | 7221.6 (5800.5-8957.2) | -0.67 (-0.78--0.56) |
| Puerto Rico | 73816.4 (60397.9-87928.1) | 4002.7 (3275.1-4767.9) | 61081.4 (48987.3-74263.5) | 3854.1 (3091-4685.9) | -0.32 (-0.39--0.24) |
| Jordan | 119491.3 (94299.7-148584.6) | 6680.3 (5272-8306.8) | 389633.2 (307016.6-482492.1) | 6149.5 (4845.6-7615.1) | -0.51 (-0.59--0.44) |
| Ghana | 356611.6 (283117.7-439280.6) | 5208.8 (4135.3-6416.3) | 853845.5 (677112.6-1049239.8) | 5140.9 (4076.8-6317.3) | -0.13 (-0.17--0.09) |
| Greece | 366674.6 (292406-449618) | 7261.5 (5790.7-8904.1) | 343344.4 (274602.5-421283.8) | 7679.2 (6141.8-9422.4) | 0.04 (-0.35-0.44) |
| Yemen | 424897 (336064.7-540878.9) | 7795.6 (6165.8-9923.6) | 1212140.5 (949928.9-1500832) | 7749.6 (6073.2-9595.3) | -0.06 (-0.07--0.04) |
| Guatemala | 177019.3 (141381.2-217678.6) | 5101.7 (4074.6-6273.6) | 470309.6 (368264.3-578621.4) | 4914.5 (3848.2-6046.3) | -0.33 (-0.48--0.19) |
| Seychelles | 1097.8 (887.8-1346.8) | 2963.2 (2396.3-3635.5) | 1448.6 (1173.3-1745.3) | 2673.5 (2165.5-3221.1) | -0.45 (-0.52--0.38) |
| Guinea-Bissau | 22956.5 (18167.3-28366.4) | 5189.8 (4107.1-6412.8) | 50356.7 (39398.1-61914.5) | 5321.7 (4163.6-6543.1) | 0.1 (0.08-0.11) |
| Costa Rica | 63559.1 (51502.7-78181.8) | 4125 (3342.5-5074) | 112959.9 (91649-138488.1) | 4526.4 (3672.4-5549.3) | 0.36 (0.33-0.38) |
| Zambia | 164407.5 (130675.4-203490.9) | 4654.7 (3699.7-5761.2) | 420633.4 (333844.8-523847.9) | 4659.4 (3698-5802.7) | -0.1 (-0.15--0.05) |
| United Kingdom | 2022344 (1655901.1-2417609.6) | 7101.3 (5814.6-8489.2) | 1914885.6 (1571876.5-2292565.6) | 6282.1 (5156.8-7521.2) | -0.39 (-0.56--0.22) |
| Denmark | 178340.9 (146465.6-214059.4) | 6673.4 (5480.6-8009.9) | 120676.5 (99371.4-144728) | 4707.6 (3876.5-5645.8) | -1.38 (-1.53--1.22) |
| Switzerland | 242432 (202877.8-288194.7) | 6682.2 (5592-7943.6) | 206034.1 (175033.7-239833.9) | 5158.6 (4382.5-6004.9) | -1.08 (-1.46--0.69) |
| Republic of Moldova | 99633.4 (79922.2-122577.3) | 4531.5 (3635-5575) | 70399.8 (57236.7-86725.8) | 3832.1 (3115.6-4720.7) | -0.7 (-0.79--0.61) |
| France | 1866086.4 (1672525.2-2069027.5) | 6396.8 (5733.2-7092.4) | 1592675.6 (1287272-1929608.2) | 5601.4 (4527.3-6786.4) | -0.48 (-0.59--0.37) |
| Gabon | 38751.1 (30617.5-48721.5) | 8708.7 (6880.8-10949.4) | 76933 (61119.5-95992.6) | 8328.8 (6616.8-10392.2) | -0.22 (-0.27--0.17) |
| Djibouti | 11623.7 (9208.5-14336.5) | 4974.4 (3940.8-6135.4) | 34025.2 (26956-42163.7) | 5298.5 (4197.7-6565.8) | 0.18 (0.16-0.19) |
| Brunei Darussalam | 2837.9 (2283.6-3490.6) | 1940.2 (1561.2-2386.3) | 5115.9 (4111.5-6245.7) | 1922.7 (1545.2-2347.3) | -0.13 (-0.22--0.05) |
| American Samoa | 712.4 (572.6-886.6) | 2924.1 (2350.1-3639.1) | 770.6 (614.4-949.4) | 2806.7 (2237.9-3457.8) | -0.21 (-0.27--0.16) |
| Sri Lanka | 417790.3 (345980.9-503871.5) | 4545.3 (3764.1-5481.9) | 362298.7 (300046.4-431272) | 3322.7 (2751.8-3955.3) | -1.39 (-1.6--1.18) |
| Burundi | 166878.9 (131022.5-206084.9) | 6885.4 (5406-8503.1) | 299995.7 (237538.7-368961.9) | 5473.5 (4334-6731.8) | -1.05 (-1.15--0.95) |
| Iraq | 456179.6 (365625.2-557983) | 5832.7 (4674.8-7134.3) | 1365044 (1091628.7-1658339.8) | 5905.3 (4722.5-7174.1) | -0.04 (-0.48-0.4) |
| Dominican Republic | 190149.4 (149721.3-237300.5) | 5226.6 (4115.3-6522.6) | 304608 (242246.8-370179.3) | 5279.3 (4198.5-6415.8) | -0.04 (-0.09-0.02) |
| Guinea | 133432.6 (105981.5-163657.6) | 5083.4 (4037.6-6234.9) | 294914.8 (236166.9-364001.6) | 5154.1 (4127.4-6361.5) | 0.06 (0.06-0.07) |
| Afghanistan | 374680.6 (296517.6-462384.8) | 7864.4 (6223.8-9705.3) | 1407145 (1123458.9-1736250.5) | 7732 (6173.2-9540.3) | -0.02 (-0.06-0.01) |
| North Macedonia | 30304.5 (24522.4-36641.3) | 2895.4 (2343-3500.9) | 29553.6 (23856.1-35632.6) | 2726.4 (2200.8-3287.2) | -0.34 (-0.4--0.29) |
| Honduras | 76046 (60343.2-93774.5) | 3714.5 (2947.5-4580.5) | 199647.9 (160712.1-247276.7) | 3858.1 (3105.7-4778.5) | 0.18 (0.16-0.2) |
| Bangladesh | 3428047.7 (2763782.1-4181953.7) | 6795 (5478.3-8289.3) | 5527861.2 (4448126.7-6734285.7) | 6415.4 (5162.3-7815.6) | -0.21 (-0.27--0.16) |
| Tokelau | 26.6 (19.8-35.5) | 3835.4 (2853.9-5110.7) | 24.6 (18.6-32) | 3806.3 (2877.6-4963.3) | -0.04 (-0.06--0.02) |
| Lesotho | 59838 (47060.6-74443.5) | 7246.5 (5699.1-9015.2) | 84356.7 (67684.9-103114.4) | 7353.8 (5900.4-8989) | 0.32 (0.22-0.42) |
| Uganda | 643544.9 (506718.7-802335.5) | 8656 (6815.6-10791.8) | 1550348.2 (1233244.2-1920998.6) | 8211.3 (6531.8-10174.4) | -0.69 (-1.09--0.29) |
| Argentina | 600699.9 (485886.7-729702.9) | 3777.6 (3055.6-4588.9) | 877185.3 (752561.6-1009207.3) | 3782.5 (3245.1-4351.7) | -0.04 (-0.1-0.03) |
| Tuvalu | 174.1 (130.3-228) | 3860.4 (2890-5055.7) | 229.5 (171.2-302.7) | 3803.8 (2837.7-5015.8) | -0.06 (-0.08--0.05) |
| Barbados | 5629.4 (4526.8-6968.6) | 4178.3 (3359.9-5172.3) | 6129 (4931.9-7499.4) | 4372.1 (3518.1-5349.7) | 0.19 (0.15-0.23) |
| San Marino | 703.8 (529.8-910.5) | 5861.6 (4412.1-7583.1) | 919.1 (692.4-1210.8) | 6072.1 (4574.6-7999.6) | 0.15 (0.13-0.16) |
| Comoros | 10108.6 (8115.5-12392.3) | 4937.8 (3964.2-6053.3) | 17798.9 (14089.7-21997.3) | 4764.3 (3771.5-5888.2) | -0.22 (-0.28--0.16) |
| Botswana | 29861.1 (23792.6-37260.5) | 4956.2 (3949-6184.3) | 70591.1 (55684-88975.9) | 5336.5 (4209.6-6726.3) | 0.14 (0.06-0.21) |
| Sudan | 647806.3 (511408-807348.6) | 7115.4 (5617.2-8867.8) | 1425624.1 (1138523.8-1764803.5) | 6840.1 (5462.6-8467.4) | -0.2 (-0.23--0.17) |

| **TableS6: Annual percentage change for depression prevalence overall (net drift) by sex from 1990 to 2019** | | | | |
| --- | --- | --- | --- | --- |
| Net Drift (%/year) | 95%CI_low | 95%CI_high | location | sex |
| -0.190844325 | -0.207896965 | -0.173788771 | Andean Latin America | Male |
| -0.250807748 | -0.269059397 | -0.232552758 | Eastern Europe | Male |
| -0.253441488 | -0.287313248 | -0.219558223 | Global | Male |
| -0.194820339 | -0.204421911 | -0.185217844 | Central Sub-Saharan Africa | Male |
| -0.525633115 | -0.572553439 | -0.478690649 | Low-middle SDI | Male |
| -0.218833328 | -0.245487096 | -0.192172439 | Caribbean | Male |
| -0.249500001 | -0.272826653 | -0.226167892 | Eastern Sub-Saharan Africa | Male |
| -1.129325956 | -1.219444421 | -1.039125276 | East Asia | Male |
| 0.129881629 | 0.049038496 | 0.210790087 | High-income North America | Male |
| -0.767520816 | -0.834358767 | -0.700637815 | South Asia | Male |
| -0.28284346 | -0.299985796 | -0.265698176 | Low SDI | Male |
| 0.208166135 | 0.139723397 | 0.276655652 | High-income Asia Pacific | Male |
| -0.476451801 | -0.529387289 | -0.423488142 | High-middle SDI | Male |
| -0.115599148 | -0.149683046 | -0.081503615 | Oceania | Male |
| 0.179890729 | 0.153827475 | 0.205960765 | Central Latin America | Male |
| -0.284167714 | -0.306613415 | -0.261716959 | Central Europe | Male |
| 0.282325256 | 0.267377502 | 0.297275238 | Southern Sub-Saharan Africa | Male |
| -0.110147846 | -0.232338985 | 0.012192947 | Australasia | Male |
| -0.140507114 | -0.166836974 | -0.11417031 | Southeast Asia | Male |
| -0.240536401 | -0.267032854 | -0.214032908 | Southern Latin America | Male |
| -0.374440769 | -0.485179336 | -0.263578975 | Tropical Latin America | Male |
| 0.048188261 | 0.035828935 | 0.060549114 | Central Asia | Male |
| 0.125665268 | 0.111901921 | 0.139430507 | North Africa and Middle East | Male |
| -0.222717475 | -0.274185629 | -0.17122276 | Western Sub-Saharan Africa | Male |
| 0.113141481 | 0.07244277 | 0.153856744 | High SDI | Male |
| -0.296501296 | -0.332395008 | -0.260594658 | Middle SDI | Male |
| -0.174287244 | -0.19650357 | -0.152065973 | Western Europe | Male |
| -0.304076641 | -0.327411067 | -0.280736753 | Andean Latin America | Female |
| -0.312183097 | -0.345840656 | -0.278514169 | Eastern Europe | Female |
| -0.362960274 | -0.408128907 | -0.317771155 | Global | Female |
| -0.156885899 | -0.174965453 | -0.138803071 | Central Sub-Saharan Africa | Female |
| -0.774164348 | -0.829461126 | -0.718836738 | Low-middle SDI | Female |
| -0.681569997 | -0.725167756 | -0.637953091 | Caribbean | Female |
| -0.366728865 | -0.390300611 | -0.34315154 | Eastern Sub-Saharan Africa | Female |
| -1.231310191 | -1.342536299 | -1.119958688 | East Asia | Female |
| 0.273671124 | 0.152514319 | 0.394974496 | High-income North America | Female |
| -1.06725675 | -1.137451629 | -0.997012032 | South Asia | Female |
| -0.407217926 | -0.42822277 | -0.38620865 | Low SDI | Female |
| 0.180090226 | 0.104718352 | 0.255518851 | High-income Asia Pacific | Female |
| -0.503253304 | -0.567168394 | -0.439297129 | High-middle SDI | Female |
| -0.125346387 | -0.156046493 | -0.094636841 | Oceania | Female |
| 0.559530242 | 0.503763984 | 0.615327443 | Central Latin America | Female |
| -0.54572238 | -0.571005644 | -0.520432687 | Central Europe | Female |
| 0.000582559 | -0.019489591 | 0.020658738 | Southern Sub-Saharan Africa | Female |
| 0.278129074 | 0.218139484 | 0.338154573 | Australasia | Female |
| -0.169943662 | -0.201812128 | -0.138065019 | Southeast Asia | Female |
| -0.413012733 | -0.442330549 | -0.383686283 | Southern Latin America | Female |
| -0.508876366 | -0.62157662 | -0.396048304 | Tropical Latin America | Female |
| -0.082442076 | -0.101787013 | -0.063093394 | Central Asia | Female |
| -0.025294725 | -0.050687484 | 0.000104486 | North Africa and Middle East | Female |
| -0.348146704 | -0.426143761 | -0.270088552 | Western Sub-Saharan Africa | Female |
| 0.231710034 | 0.163529768 | 0.299936709 | High SDI | Female |
| -0.407354307 | -0.453220325 | -0.361467156 | Middle SDI | Female |
| 0.00439786 | -0.014756099 | 0.023555489 | Western Europe | Female |
| -0.274203471 | -0.293523554 | -0.254879644 | Andean Latin America | Both |
| -0.28704026 | -0.309899754 | -0.264175524 | Eastern Europe | Both |
| -0.316068039 | -0.355565703 | -0.276554719 | Global | Both |
| -0.180505406 | -0.194459608 | -0.166549254 | Central Sub-Saharan Africa | Both |
| -0.665197708 | -0.716802746 | -0.613565846 | Low-middle SDI | Both |
| -0.532017947 | -0.566454816 | -0.497569153 | Caribbean | Both |
| -0.325780238 | -0.347740116 | -0.30381552 | Eastern Sub-Saharan Africa | Both |
| -1.179883001 | -1.275217711 | -1.084456229 | East Asia | Both |
| 0.219822462 | 0.112393899 | 0.327366304 | High-income North America | Both |
| -0.92842208 | -0.997102375 | -0.859694141 | South Asia | Both |
| -0.355068606 | -0.375309835 | -0.334823264 | Low SDI | Both |
| 0.184457865 | 0.118858765 | 0.250099948 | High-income Asia Pacific | Both |
| -0.501872004 | -0.559439772 | -0.44427091 | High-middle SDI | Both |
| -0.116384577 | -0.139191352 | -0.093572593 | Oceania | Both |
| 0.421327111 | 0.380673108 | 0.461997578 | Central Latin America | Both |
| -0.452558644 | -0.47422621 | -0.43088636 | Central Europe | Both |
| 0.085391301 | 0.071188014 | 0.099596604 | Southern Sub-Saharan Africa | Both |
| 0.130628597 | 0.052003507 | 0.209315473 | Australasia | Both |
| -0.170063561 | -0.199091936 | -0.141026743 | Southeast Asia | Both |
| -0.355845023 | -0.382834272 | -0.328848462 | Southern Latin America | Both |
| -0.471650721 | -0.58228314 | -0.36089519 | Tropical Latin America | Both |
| -0.037527043 | -0.051641218 | -0.023410874 | Central Asia | Both |
| 0.015592859 | -0.000481081 | 0.031669384 | North Africa and Middle East | Both |
| -0.265733987 | -0.333806265 | -0.197615215 | Western Sub-Saharan Africa | Both |
| 0.171655123 | 0.113544253 | 0.229799723 | High SDI | Both |
| -0.352399516 | -0.390910322 | -0.31387382 | Middle SDI | Both |
| -0.064445492 | -0.078990859 | -0.049898007 | Western Europe | Both |
